# Quantifying the Characteristic Turnover Frequency of an Individual Redox Enzyme via Real-Time Electrical Transduction of Catalytic Events in a Label-Free Single-Protein Junction

**DOI:** 10.1021/jacs.5c23035

**Published:** 2026-07-31

**Authors:** Tracy Quynh Ha, Albert C. Aragonès, Qiankun Wang, Desmond Koomson, Nashili Kibria, Sofia Margiola, Yongchan Jeong, Jhanelle White, Kavita Garg, Jessica Peate, Alex P.S. Brogan, Leigh Aldous, Sarah M. Barry, Ismael Díez-Pérez

**Affiliations:** † Department of Chemistry, Faculty of Natural, Mathematical & Engineering Sciences, 4616 King’s College London, Britannia House, 7 Trinity Street, London SE1 1DB, U.K.; ‡ Departament de Ciència de Materials i Química Física & Institut de Química Teòrica i Computacional (IQTC), Universitat de Barcelona, Martí I Franquès 1, Barcelona 08028, Spain; § Department of Chemistry, School of Physical Sciences, DIT University, Dehradun 248001, India; ∥ Department of Chemical Engineering, National Taiwan University, Roosevelt Road, Taipei 106, Taiwan

## Abstract

Understanding redox enzymatic reactions remains a fundamental challenge in biochemistry, as these processes involve intricate dynamics that are inherently difficult to dissect using conventional bulk techniques. Single-enzyme catalysis offers a promising approach for unraveling the dynamic behavior of individual enzymes as they undergo a reaction, revealing the complex heterogeneity that is lost in the averaged ensemble. Here, we demonstrate real-time, label-free monitoring of the electrical transduction of single-protein enzymatic activity for two redox enzymes, cytochrome P450_cam_and glutathione reductase, trapped in an electrochemically controlled nanoscale tunneling junction immersed in the aqueous enzymatic mixture. The conductance switching signal observed in individual transients of the electrical current flowing through the single-protein junction shows that the tunneling conductance is modulated by the enzymatic reaction; subtle changes in the enzyme redox state occurring during the chemical catalysis process result in fluctuations of the enzyme junction conductivity, which are captured as a switching signal. At the applied electrochemical reducing potential for electrocatalysis, the transient oxidation of the trapped enzyme in every catalytic cycle opens an additional redox-mediated electron tunneling channel in the single-protein junction that results in a temporary tunneling current jump, contributing to the observed conductance switching feature. The latter is experimentally corroborated via electrochemically controlled conductance measurements of the single-protein junction. The statistical analysis of the switching events captured over long time periods results in average frequencies that correlate well with the reported catalytic turnover values of both enzymes obtained in standard bulk assays. The single-enzyme experiments reveal the acute heterogeneous behavior of enzymatic catalysis and the quantification of single-enzyme turnover frequencies. This work demonstrates a new nanoscale platform for nonlabeled electrical detection of single-enzyme activity in a redox enzymatic process.

## Graphical abstract

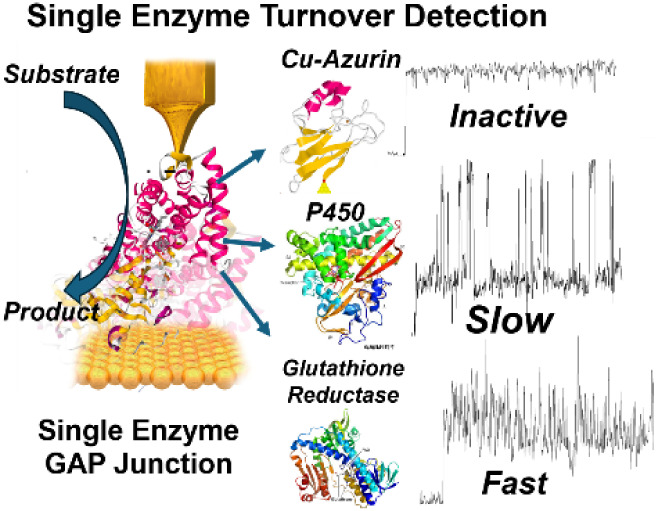

## Introduction

Redox enzymes employ both specialised organic (FAD, SAM), organometallic (heme, cobalamin) or metal ion (e.g. Fe, Ni, Cu) cofactors to effect a vast array of kinetically demanding chemical transformations often without equivalent in synthetic chemistry.^1^ There have significant advances in understanding redox enzyme structure and catalytic mechanisms through a range of techniques including X-ray crystallography, EPR amd Crio-EM, however we still have very little understanding about the main forces driving the enzyme’s outstanding ability to accelerate chemical reactions, in some instances, million times faster than we can achieve by synthetic means, and with remarkable chemo-, stereo- and regio-specificity. The enzyme's catalytic power has led to extensive efforts to utilize enzymes as biocatalysts in the chemical synthesis of chiral building blocks and high-value products such as pharmaceuticals.^2,3^ Understanding the fundamental mechanism of enzymatic catalysis is also key to inform protein engineering toward improved enzymes for practical biotechnology. Traditional ensemble methods investigating enzymatic activity generally provide averaged results, which do not reflect intracellular environments that exhibit high heterogeneity.^4^ Single-protein junctions have brought new prospects into single-enzyme studies,^5,6,7,8^ enabling high-resolution and high-throughput single-protein electrical characterization to access complex enzymatic reaction mechanisms.^6^ Scanning tunneling microscopy (STM)-based molecular tunneling junctions have proven to be very sensitive to the oxidation state of the molecule. Early experiments by Tao demonstrated single-molecule visualization of redox-dependent current enhancement on a redox *heme* cofactor using an electrochemical STM (EC-STM).^9^ Ulstrup and Kuznetsov later demonstrated the same effect on a redox Cu-azurin protein, showing that the tunneling current flowing through an individual redox protein is strongly regulated by the redox state of the protein’s metal cofactor.^10,11^ Following this pioneering work, we demonstrated *in situ* real-time dynamic detection of transient redox changes in an engineered Cu-azurin trapped in an EC-STM-based nanogap.^7^ The latter opens up a whole new vista in single-protein detection of redox enzymatic catalysis, as the redox enzymatic process of a single trapped enzyme in a tunneling junction can be followed via dynamical changes in the redox state occurring during the catalytic cycle. This behavior has been long predicted by Albrecht et al., who proposed that the tunneling conductance of an active trapped enzyme in a nanogap would produce a discrete conductance switching signal generated by individual catalytic turnover events.^12^ Similar switching behavior has been observed in previous single-protein fluorescence microscopy studies. For example, Lu et al. investigated the catalytic process of a FAD-dependent redox enzyme, cholesterol oxidase (COx).^13^ They observe that the COx fluorescence intensity fluctuates in the presence of its substrate and describe the phenomenon as on–off behavior resulting from enzymatic turnover when FAD toggles between oxidized (on state) and reduced (off state).^13^ While fluorescence detection has been shown to reach single-enzyme detection levels,^14,15,16,17^ its broad applicability is challenging as (1) it requires fluorescent labels that necessitate enzyme modification, which limits the scope of enzymes that can be studied, and (2) it is prone to photobleaching and pH dependencies,^18^ limiting long-term studies and throughput. Very recent pioneering work in the area of single-protein electronics has also shown that tunneling current noise produced by a single nonredox Φ29 polymerase trapped in a nanogap can be ascribed to enzymatic activity.^5^ Zhang et al. also exploited single-protein junctions to capture intermediate states of the catalytic cycle in the enzyme formate dehydrogenase, proving that different catalytic states can be ascribed to different protein conductance states.^6^

Herein, we combine all the above pioneering efforts in redox single-protein junctions to develop a new methodology for real-time, label-free, single-protein electrical transduction of redox enzymatic catalysis. We demonstrate the methodology by studying two very different unmodified redox enzymes, namely, cytochrome P450_cam_ and glutathione reductase (GR), with contrasting active redox cofactors *heme* and flavin adenine dinucleuotide (FAD), respectively, catalyzing very different chemistries at very disparate catalytic rates (see Results section). Under catalytic conditions, individual trapped enzymes in an electrochemically controlled nanogap at a fixed potential generate distinct two-level tunneling conductance fluctuations matching the ones experimentally observed for the same single-enzyme junctions under varying electrochemical potentials and nonactive catalytic conditions. The characteristic frequency of the observed conductance switching is directly compared against the reported enzymes’ catalytic rates by collecting thousands of single-protein catalytic events and averaging them over the total residence time of the trapped proteins in the nanogap. The analysis shows an exquisite agreement between the observed single-enzyme electrical fluctuations collected in long time periods and the bulk enzyme catalytic rates of the two enzymes. The latter validation suggest a new methodology for real-time, label-free, electrical detection of catalytic events in an individual redox enzyme, enabling (1) the quantification of catalytic heterogeneity (ON-OFF periods) in redox enzymatic processes, (2) the quantification of the characteristic single-enzyme catalytic frequencies, and (3) an electronic sensing platforms for high-resolution analytical quantification of enzymatic activity.

## Results

### Preparation of Enzymes/Electrode Interfaces for Single Enzyme Entrapment

Cytochrome P450 enzymes are a ubiquitous superfamily of *heme*-dependent enzymes with vital roles in many biochemical pathways, such as hormone biosynthesis, and have long been important in drug development due to their role in xenobiotic metabolism.^2,3,19^ As we attempt to transition to more sustainable chemical processes, P450s have gained importance as biocatalysts.^19,20^ This is due to their ability to catalyze a wide variety of synthetically challenging reactions such as hydroxylation, epoxidation, and C–C bond formation via regio- and stereoselective activation of inert C–H bonds, using molecular oxygen as a cosubstrate.^21^ In this work, we focus on the well-characterized cytochrome P450_cam_, which catalyzes the kinetically difficult hydroxylation of D-camphor to 5-*exo*-hydroxycamphor at modest catalytic rates (*k*_cat_) of 124–3960 min^–1^,^22,23,24^ and compare it to GR, which catalyzes the less complex reduction of oxidized glutathione (GSSG) to reduced glutathione (GSH) at a much higher *k*_cat_ of 12600–17500 min^–1^ ^25,26,27^ (see comprehensive catalytic cycles in Supplementary Figure S24-25).

Recombinant cytochrome P450cam was produced as an N-terminal His6-tagged fusion protein and demonstrated to be catalytically active with substrate camphor to produce 5-exo-hydroxycamphor in biochemical assays analysed by GC-MS (Supplementary Figure S2-S8). Glutathione reductase was purchased (Aldrich) and its activity in solution was confirmed by spectophotometric NADPH consumption assay (Supplementary Table S1). Unmodified recombinant P450_cam_ or GR enzymes were adsorbed via nonspecific van der Waals interactions on an atomically flat Au(111) electrode surface functionalized with a 1-octanethiol self-assembled monolayer (SAM), providing a soft electrode/protein contact. Under our preparation conditions (see Methods), we do not produce a compact SAM but rather a defective molecular layer as suggested by the low layer thickness measured in AFM scratching experiments (Supplementary Figure S17). As such, significant penetration of the proteins into the SAM upon absorption via defective sites will be expected, thus maximizing both electrode/protein interaction and electrical coupling. The interfaces are denoted as P450_cam_-C8-Au (Figure 1a) and GR-C8-Au (Figure 1b), respectively, for the two enzymes. The two interfaces were imaged at high resolution using an EC-STM (Figure 1c and d, respectively) under potentiostatic conditions in 0.1 M phosphate (pH 7) and 0.1 M Tris-KCl buffers (pH 7) for P450_cam_ and GR, respectively. The dark pits observed in the image background are indicative of the formation of the octanethiol SAM.^28^ Embedded in the alkane SAM, the enzymes appear as brighter spots, which are stable upon successive STM image scans, showing successful enzyme adsorption. Extensive protein STM imaging has been previously reported,^11,29,30a^ including on alkane-modified electrode surfaces.^30b^ Under electrochemical conditions, substantially larger STM gaps can be established thanks to the much lower tunneling current attenuations in the highly conductive working electrolyte (Supplementary Figure S13),^30c^ enabling protein imaging. The average apparent diameters of both enzymes in the STM images (Supplementary Figure S16) result in average values of 5.4 and 10 nm for P450_cam_ and GR, respectively, consistent with the differences in the crystallographic size between the two proteins (Supplementary Figure S14). The slight enzyme-to-enzyme contrast variability of the proteins in the STM images denotes small variations in their orientations on the electrode surface together with the conductivity mapping nature of the STM images which tends to enhance the more conductive areas around the redox cofactor.^9^ The latter is also responsible for the observed anomalously small apparent protein heights in the STM image, typically of only several Å, which reflect their lower conductance as compared to the electrode substrate underneath.^9,11,30a^ The limited spatial resolution of the topographical images in tapping mode atomic force microscopy (AFM) allowed imaging the large GR only (Supplementary Figure S15), measuring average protein heights of 6.5 nm and suggesting the enzymes are generally ”lying down” on the electrode surface (Supplementary Figure S14). At pH 7, the surface electrostatics of both proteins display large hydrophobic patches (Supplementary Figure S27) able to sustain van der Waals interactions with the functionalized electrode surface. To further corroborate the STM images of individual proteins, we have conducted electrochemical potential-dependent STM imaging of the P450_cam_-C8-Au. The STM imaging contrast in redox molecules depends on the energy alignment of the redox state with the Fermi energy of both STM electrodes, which enhances tunneling probability when in resonance and results in an increase in the apparent heights. This has been demonstrated in several redox proteins,^7,11,31^ including enzymes.^32^ We observe an average increase in the apparent STM height of P450_cam_ at the enzyme redox midpoint potential −0.35 V (Figure 1e), in accordance with the cyclic voltammetry (CV) results (Figure 3a), which reinforces the assignment of the bright spots in STM images to individual redox enzymes.

**Figure 1:**
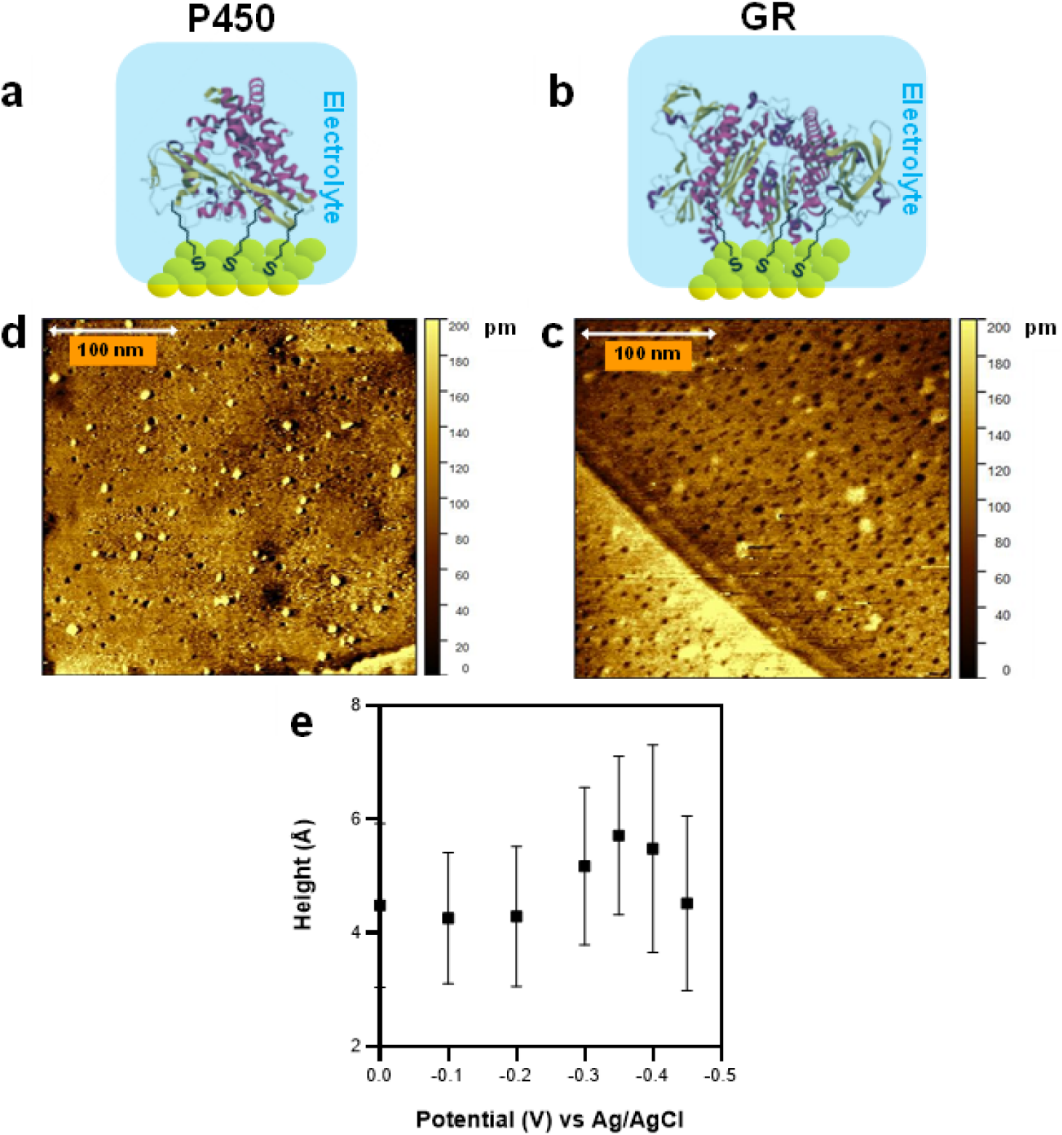
EC-STM imaging of P450_cam_-C8-Au and GR-C8-Au interfaces. Schematic representation of **a:** P450_cam_-C8-Au and **b:** GR-C8-Au interfaces, respectively. EC-STM images of c: P450_cam_ in 0.1 M phosphate buffer (pH 7) and **d:** GR in 0.1 M Tris-KCl (pH 7), respectively, immobilized on 1-octanethiol-functionalized Au(111), with the Au(111) subjected to an electrochemical potential of 0.1 V vs AgCl reference electrode. **e:** Average STM apparent height of individual P450_cam_ as a function of the applied electrochemical potential. All images were collected with Au(111) working electrode, Pt counter electrode, and Ag/AgCl (3 M KCl) reference electrode. V_bias_ = +0.1 V, current set point = 0.05 nA.

Next, we trap individual enzymes in a static, electrochemically controlled nanoscale gap (Figure 2a-b) as follows: the STM tip electrode is brought into proximity to the thiol-modified Au(111) substrate, creating gap distances ranging from 4.0 to 5.7 nm for GR and 3.8–5.2 nm for P450_cam_ (gap distance calibration details in Supplementary Figure S13). The largest distances were achieved by setting a small tunneling current set point (a few tens of pico-Ampers) at an applied bias voltage difference (V_bias_= U_sample_ – U_tip_) of +0.1 V between the two junction electrodes. Once the STM tip-to-substrate gap is mechanically stable, the current feedback loop is deactivated, and the tunneling current continuously monitored during the lifetime of stability of the tunneling nanogap (typically <1 s). During this time, spontaneous enzyme trapping events between the two Au electrodes, namely the STM probe and Au substrate, lead to an abrupt increase in the measured tunneling current (Figure 2c–d top panels), which manifests as distinct “*jumps”* or “*blinks*” of short duration (∼10–100 ms). Thousands of these blinks were accumulated without data selection in 1D conductance histograms (Figure 2c-d, lower panel respectively for P450 and GR), and using Gaussian distribution functions, we yield average conductance values for the two proteins: 2.2 × 10^–5^ G_0_ and 2.9 × 10^–5^ G_0_ for P450_cam_ and GR, respectively (G_0_ = 2e^2^/h ≈ 77.4 μS). These conductance values are in good agreement with the conductance previously measured for redox proteins of comparable size.^7,33^

**Figure 2:**
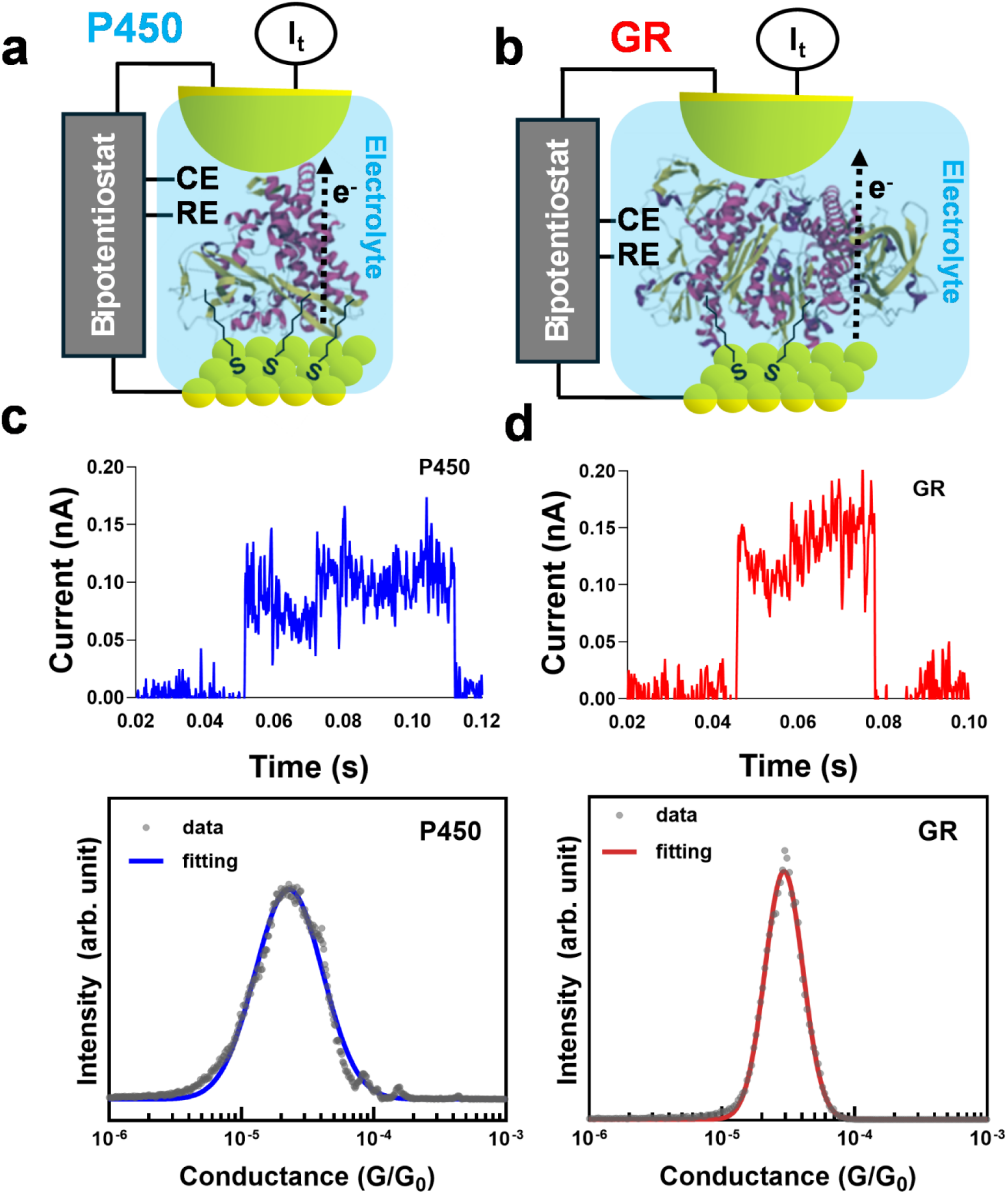
Single-protein conductance measurements. Illustrative schemes of the the trapped enzyme junctions for a: P450_cam_ and b: GR. Representative current–time traces for **a:** P450_cam_-C8-Au and **b:** GR-C8-Au. Corresponding 1D conductance histograms built by accomulating ∼3200–4000 of the current–time traces are represented in the lower c and d panels., yieldingaverage conductance values of 2.2 × 10^–5^ G_0_ and 2.9 × 10^–5^ G_0_, respectively for P450_cam_ and GR. EC-STM current–time measurements were obtained at an electrochemical potential = +0.1 V, V_bias_ = +0.1 V, low current set points = 40 pA with Au(111) and STM probe as the working electrodes, Pt as the counter electrode, and Ag/AgCl (3 M KCl) as the reference electrode.

### Electrocatalytic Activity of the P450_cam_-C8-Au and GR-C8-Au Interfaces

The electrocatalysis of both the P450-C8-Au and GR-C8-Au interfaces was probed electrochemically, and the electrocatalytic activity was confirmed by analyzing the extracted reaction buffer using gas chromatography-mass spectrometry (GC-MS) and UV–visible absorption, respectively, to analyze product formation. The electrocatalysis was first probed with all the reaction components in solution. In the absence of the natural electron source, NADPH or additional reedox partner proteins (Supplementary Figure S9a), the electrocatalytic reaction is driven by the electrode and requires either the diffusion of the enzyme to the electrode surface or an electron mediator shuttling the electrons from the electrode surface to the enzyme in solution (Supplementary Figure S9b). Figure 3a and b displays the electrocatalysis of P450_cam_ and GR, respectively, studied by CV. The two traces in each CV correspond to the redox (noncatalytic) and catalytic conditions, namely, the absence (P450_cam_) and presence of the D-camphor substrate (P450_cam_/camphor) in Figure 3a, and the absence (MV/GSSG) and presence of the enzyme (MV/GSSG/GR) in Figure 3b, where MV refers to the redox mediator methyl viologen dichloride. The voltametric signals obtained are concordant with previous literature: (1) P450_cam_ presents a redox peak between −0.4 V and −0.6 V^34,35,36^ with observed electrochemical current enhancement at −0.27 V in the presence of D-camphor. The redox midpoint shifts by ∼0.17 V as the enzyme goes from its substrate-free to substrate-bound form,^37,38,39^ as observed by the slightly more positive electrocatalytic signal, indicating catalytic transformation of camphor to 5-*exo*-hydroxycamphor (Figure 3a, top panel).^36^ Additionally, the shift in P450_cam_ redox potential accompanies changes in the optical spectrum of the enzyme upon camphor binding due to the Fe­(III) in the *heme* cofactor going from a low to high spin state (Supplementary Figure S4).^40^ The increase in cathodic (negative) current coincides with the observed increase in the enzyme’s apparent height in the STM image for the surface-absorbed P450_cam_ (Figure 1e). (2) The electrocatalytic reduction of GSSG to GSH by GR is mediated by MV as the electron mediator between the enzyme and the electrode surface (Figure 3b, top panel).^41,42,43^ MV provides a means to record the electrocatalytic activity of GR via the MV electrochemistry, since GR displays no redox signal within the aqueous electrochemical potential range. MV is the only species that displays redox chemistry at −0.68 V, while GSSG and GR displayed no redox chemistry in the working potential window (Supplementary Figure S10b).^44^ The electrocatalysis of GR is a much faster reaction than the P450_cam_ resulting in higher catalytic currents, which is supported by the much larger *k*_cat_ turnover rate for GR.^22,25^

**Figure 3:**
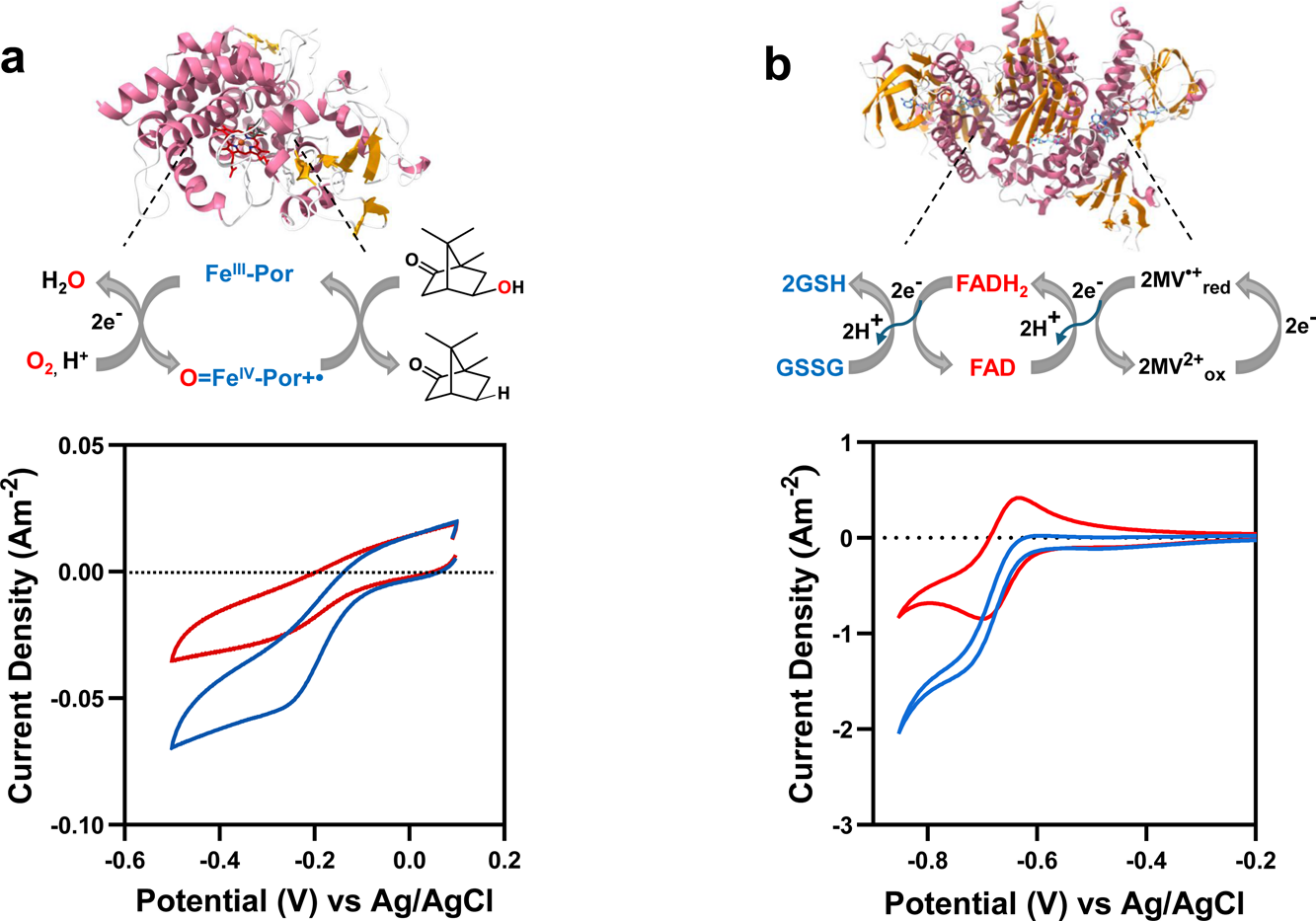
Solution-phase electrocatalytic cyclic voltammetry (CV). **a:** CVs of P450 in the absence (red) and presence of camphor (blue), using 0.1 M phosphate buffer (pH 7). **b:** CVs of MV in the presence of GSSG only (red) and in the presence of GSSG and GR (blue) using 0.1 M Tris-KCl (pH 7). The corresponding electrocatalytic reaction schemes are represented in the top panels. CVs were obtained using octanethiol-functionalized Au working electrode, Pt counter electrode, and Ag/AgCl (3 M KCl) reference electrode. Scan rates for P450 and GR electrocatalysis were 5 mV s^–1^ and 10 mV s^–1^, respectively.

The same electrocatalytic experiments were also conducted by preadsorbing the enzyme on the C8-Au electrode surface (Supplementary Figure S9c) to confirm enzymatic activity under conditions similar to those used in the single-enzyme experiments. We observed very limited enzyme coverage of the electrode surface (Figure 1c-d) resulting in insufficient enzyme to produce detectable concentrations of reaction product in a short CV-based electrocatalytic assays. To overcome this limitation, chronoamperometric experiments were carried out to drive the electrocatalytic reaction over long time periods of 3 h for GR and up to 7 h for P450_cam_. For P450cam, electrocatalytic assays were also analysed via GC-MS and compared the results to those from biochemical assays to confirm product formation (Supplementary Figures S11-S12). For GR, spectrophotometric assays were used to calculate the amount of substrate remaining after electrocatalysis (Supplementary Figure S11). These experiments confirm that the surface adsorbed enzymes maintain catalytic activity.^45^

### Electrochemically Controlled Single-Enzyme Catalytic Junctions

Transient electrochemically controlled nanogaps were formed using an EC-STM configuration as described above for both the P450_cam_-C8-Au and GR-C8-Au interfaces containing a low coverage of proteins scattered on the electrode surface (Figure 1c–d, respectively). Sudden current jumps, resulting from individual enzyme trapping in the tunneling nanojunction (Figure 2a–b), were recorded at both oxidizing and reducing applied potentials in the presence and absence of their corresponding enzymatic substrates and/or redox mediator. A reducing potential of −0.4 V was applied to the P450_cam_C8-Au interface in oxygenated aqueous buffer containing the substrate camphor to induce the electrocatalytic enzymatic process. Molecular oxygen is a cosubstrate in the catalyzed oxidation of camphor by P450_cam_. For GR–C8-Au, the substrate GSSG and electron mediator MV are added to the buffer at a reducing applied potential between −0.5 and −0.6 V. We define these as the *active conditions,* whereby the enzymatic interfaces have been shown to undergo electrocatalysis (Figure 3). In contrast, we define various *inactive conditions* as (1) when both interfaces are in the presence of all chemical components for electrocatalysis but subjected to applied oxidizing potentials of +0.1 V, far beyond where electron injection for electrocatalysis occurs, and (2) in the absence of their catalytic components, namely, substrate and/or redox mediator, at either reducing or oxidizing potentials. These inactive conditions are control experiments where bulk electrocatalysis is suppressed (Figure 3). Two different types of protein trapping events (*blinks*) are observed during the lifetime of the tunneling nanogaps, which we denote as *silent* (Figure 4a) and *switching* (Figure 4b). An algorithm in python was coded to automate the classification of all the collected blinks under the different conditions (1000–1500 traces per condition) into the two *silent* and *switching* categories (Supplementary Figure S21). The *silent* blinks typically display a single conductance level with small fluctuations around the average protein conductance (Figure 2), in line with values obtained in previous single-protein junction work.^7^ In contrast, the *switching* blinks display a characteristic two-level fluctuation of the conductance superimposed on top of the blink during the protein residence time in the nanojunction (Figure 4b). A first visual inspection shows that the frequencies of the two-level fluctuations across the two enzymes are strikingly different, with much higher values for GR. The latter shows a first correlation between the frequency of the observed 2-level fluctuation signal and the characteristic *k*_cat_ of the enzyme. Similar fluctuating signals to those observed for GR were recently reported by Zhang et al. in a nonredox, catalytically fast (*k*_cat_ = 7200 min^–1^)^46^ Φ29-polymerase.^5^ The silent blinks observed in the active conditions were compiled in 1D histograms and compared to previous analyses of the inactive conditions (Supplementary Figure S19). With the exception of the GSSG presence, similar conductance values around (2–3) × 10^–5^ G_0_ are observed for both P450_cam_ and GR in the presence of substrate and/or mediator, indicating that substrate/mediator binding to the enzymes does not significantly impact the protein conductance measured during the single protein trapping. All switching signals recorded under the active conditions for both P450_cam_ and GR were accumulated in 1D histograms with no selection (Figure 4c–d, respectively). A bimodal distribution is observed in both cases, where G1 (Figure 4c-d, red curve) and G2 (Figure 4c–d, blue curve) correspond to the averaged two levels of conductance arising from the conductance fluctuation in the switching blink. The conductance values for G1 and G2 are 1.7 × 10^–5^ G_0_ and 4.6 × 10^–5^ G_0_ for P450_cam_ and 3.5 × 10^–5^ G_0_ and 1.5 × 10^–4^ G_0_ for GR, respectively, indicating a factor of 2.7 and 3.3 in conductance change between G1 and G2 for P450_cam_ and GR, respectively. A comparison of G1 with previous conductance values (Figure 2c–d) shows that this conductance level corresponds to the intrinsic protein conductance, while G2 is ascribed to an active enzyme state.

**Figure 4:**
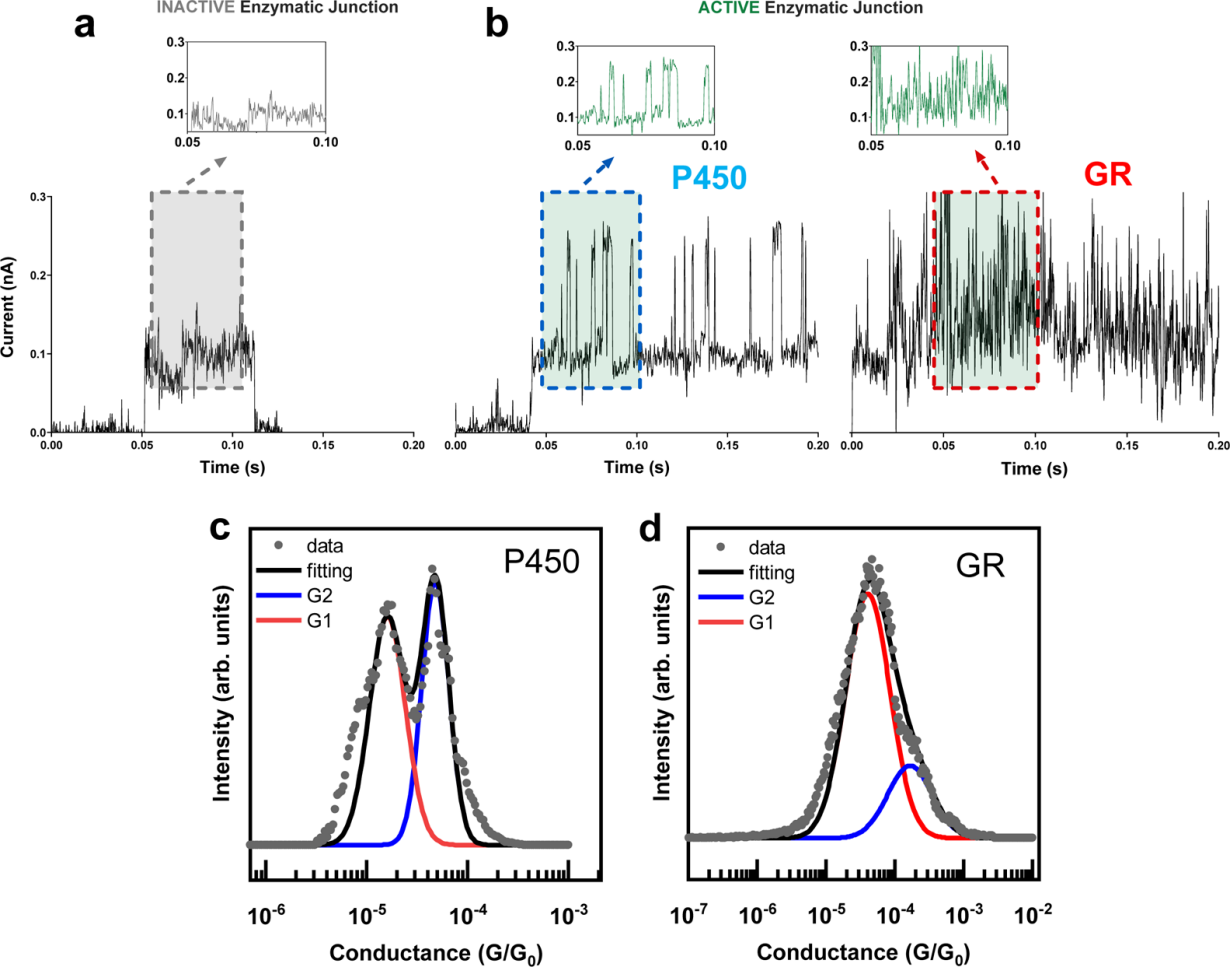
Single-protein conductance measurements under electrocatalytic conditions. **a**: Current transients of an inactive single enzymatic junction obtained in the absence of substrates. **b**: Current transients of active P450–C8–Au (left) and GR–C8-Au (right) junctions displaying switching events in the presence of their substrates. 1D conductance histograms generated by accumulating switching traces of **c:** P450 and **d:** GR in their active conditions: **c**, P450 in the presence of camphor at reducing potentials of −0.4 V and **b**: GR in the presence of GSSG and MV at reducing potentials of −0.5 V. Both experimental data (open circles) and fitting (lines) are represented, where red corresponds to the fitting of the first level (G1) and blue to the second level (G2) of conductance resulting from the switching events. Conductance values for G1 and G2 are 1.7 × 10^–5^ G_0_ and 4.6 × 10^–5^ G_0_ for P450 and 3.5 × 10^–5^ G_0_ and 1.5 × 10^–4^ G_0_ for GR, respectively. Each histogram is built from a total of ∼800–1000 current–time traces.

The previous classification also enables analysis of the percentages of observed switching blinks out of the total number of blinks (silent + switching) collected at each experimental condition for both P450_cam_ and GR (Figure 5a-b, respectively). An abrupt increase in the number of observed switching signals is consistently observed under the active conditions for both enzymes, reaching ∼30% of the total number of traces. A residual 5–7% of switching blinks is still recorded under inactive conditions, suggesting the occurrence of enzymatic events that do not lead to chemical conversion. According to previously characterized redox-active single-protein junctions,^52^ at well-cathodic overpotentials where the bulk population of the redox protein is in its reduced state, a low frequency of conductance-dependent switching is still observed, indicating the low probability for a trapped redox protein to momentarily be oxidized at the applied cathodic potential. The latter reflects the single-molecule nature of the measurement, which captures the oxidized/reduced populations at a specific applied potential. We conclude that the observed 5–7% conductance switching background under the inactive conditions mainly corresponds to electrochemically induced conductance switching resulting from the low probability of the *heme* cofactor to momentarily undergo oxidation at the cathodic electrocatalytic applied potential.

**Figure 5:**
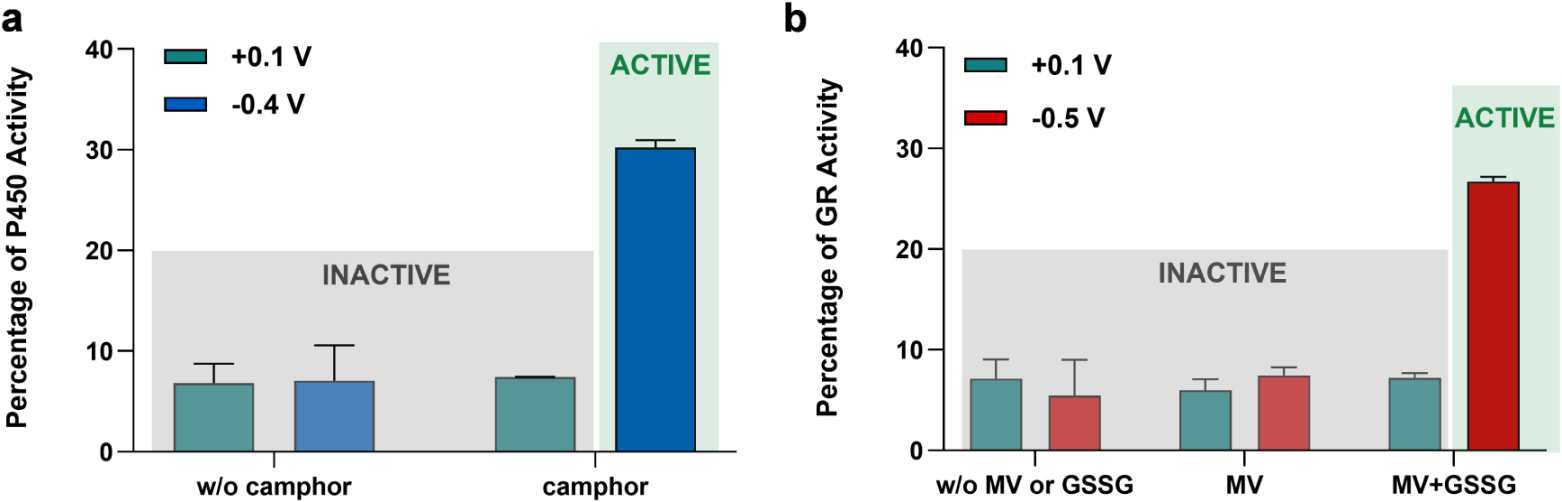
Percentage of activity in the current transient traces. Percentage of individual traces displaying conductance switching events under controlled electrochemical potentials for **a:** P450_cam_-C8-Au at +0.1 V (green) and −0.4 V (blue) in the absence and presence of camphor, and for **b**: GR–C8-Au at +0.1 V (green) and −0.5 V (red) in the absence of MV and GSSG, in the presence of MV only, and in the presence of MV and GSSG. The current transients were classified using a Python algorithm.

To seek a correlation between observed conductance switching and enzymatic activity, we first conducted an exhaustive frequency analysis of the conductance switching events using both a machine learning-based Gaussian Mixture Model (GMM)^47^ and a Hidden Markov Model (HMM)^48^ (see Supplementary section 3.2 for details). Both were able to successfully separate the two conductance levels (see Supplementary Figures S22-23). Two different characteristic frequencies were extracted for each of the two enzymes under the active conditions, which we denote as the *average single-enzyme* and the *single-enzyme* frequencies (eqs 1–2 and Table 1). The average single-enzyme frequency considers the number of times a switching event occurs in every switching trace averaged over the total protein time residence considering all signals (silent + switching) accumulated in long (>30 min) single-protein measurements (eq 1). In contrast, the single-enzyme frequency considers only the occurrence of a switching event in the active (switching) traces, ruling out time averaging over the silent (nonactive) traces (eq 2). Note that the nature of the observed conductance fluctuations is stochastic and that the frequency values here refer to the conductance switching events expressed as turnover number per minute without implying periodicity. Typically, single-molecule enzymatic experiments measure the probability density or frequency of the stochastic occurrence of individual turnover events.^49^ This has been shown in single-protein fluorescent experiments on a cholesterol oxidase enzyme, where each stochastic fluorescent blink is ascribed to an individual enzymatic turnover event, and the frequency was then calculated by considering the number of switching events per unit time.^13,50^ Table 1 summarizes the extracted frequencies from our single-protein junction experiments. The average single-enzyme frequencies of the conductance fluctuation events align well with the reported bulk kinetic *k*_cat_ for P450_cam_ ^22,23,24^ and GR (Table 1).^25,26,27^ A few considerations on the obtained frequency numbers in Table 1 are: (1) previous single-molecule fluorescence microscopy experiments suggested that each switching event may not indicate a successful enzymatic turnover, but a conformational change that leads to an inactive state.^4,13^ The conductance switching events in the single-protein experiments reflect the electrical transduction of all possible dynamics in the enzyme, whether they lead to product formation or not. This might likely result in overestimated bulk frequency values when compared to the intrinsic enzyme *k*_cat_, which are solely based on successful product formation. The latter could be partially alleviated by considering the average (5–7%) switching dynamics observed in the single-protein experiments during the inactive conditions (Figure 5) as an estimation of the average background in the single-protein dynamics leading to unsuccessful product formation. Subtracting this background from the bulk frequencies in the active conditions leads to a corrected value for the P450_cam_ and GR average single-enzyme frequencies of 1790 and 12871 min^–1^, respectively, which both lie well within the reported enzymatic turnover values for these enzymes (Table 1 ^a,e^). The large range of reported *k*_*cat*_ values, in particular for P450_cam_, stems from the enormous variability in the calculated enzymatic activity when determined using different methodologies and experimental conditions (Table 1 ^e^). To provide a more accurate comparison, we have conducted our own enzymatic assays at different concentrations of substrate to extract bulk *k*_*cat*_ values (see Supplementary sections 1.5.1 and 5) for both enzymes under equivalent electrolyte and substrate concentration conditions as those used in the single-protein assays, yielding values of ∼2700 and ∼14600 min^–1^ for P450_cam_ and GR, respectively, which aligns with the recorded average single-protein frequency values (Table 1 ^a,e^). (2) Kinetic constraints imposed by the nanogap are expected to occasionally lead to enzyme conformations with poor or no ability for the substrate to get in and/or out of the enzyme’s active site, resulting in a level of underestimation of the extracted single-enzyme frequencies. Overall, these initial proof-of-concept studies on single-enzyme frequencies indicate that technical artifacts appear to have a minimal impact on the single-protein measurements. However, widening the methodology across more enzyme families will enable the quantification of their impact on the final results and the introduction of mitigation measures via experimental design of the junctions, e.g., using single-protein junctions with controlled orientation via chemical design of the protein surface.^7^

1 $$\mathrm{Average single enzyme freq.}=\frac{\mathrm{Number of switching events}}{\mathrm{Total protein‐trapping time}}$$

2 $$\mathrm{Single enzyme freq.}=\frac{\mathrm{Number of switching events}}{\mathrm{Total time of switching traces}}$$

**Table 1:** Single-Enzyme Turnover Frequencies

	Average Single-Enzyme Frequency^a^ (Background Corrected) (min^–1^)	Single-Enzyme Frequency^b^ (Electrocatalytic Turnover)^c^ (min^–1^)	Reported^d^ (This Work)^e^ Bulk *k*_*cat*_ (min^–1^)
**P450**_**cam**_	2573 (1790)	6908 (7460)	124–3960 ^22,23,24^ (2689)
**GR**	16292 (12871)	42943 (86000)	12600–17500 ^25,26,27^ (14576)

a Average frequencies of the conductance switching events over all single-protein traces.

b Frequencies of the conductance switching events in the active single-protein traces only.

c Electrochemical turnover frequencies extracted from the net catalytic current at −0.4 and −0.7 V for P450 and GR, respectively.

d Literature *k*_cat_ values for P450_cam_ CYP101 from *P. Putida* using different methodologies via quantifying: (i) product formation,^24^ (ii) ADH oxidation,^22^ and (iii) O_2_ consumption.^23^ Literature *k*_cat_ values for yeast GR quantifying NADPH oxidation.^25^

e *k*_cat_ determined in this work from steady-state HPLC-based assays under the same solution conditions as the single-protein experiments (see SI Section 5).

The analysis of bulk frequencies (eq 1) provides a good foundation supporting the correlation between the observed single-protein conductance fluctuations and the real-time monitoring of the protein enzymatic activity. eq 2 above describes the “local” or intrinsic single-enzyme frequency, which characterizes the enzymatic activity of a single enzyme within a population where dynamic activity disorder exists. We observe a ∼3-fold increase for both enzymes when going from the average to the single-enzyme frequencies (Table 1 ^a‑b^), which reflects the significantly faster intrinsic enzyme activity not captured in the ensemble experiments. These single-enzyme catalytic rates have been previously reported for a horseradish peroxidase enzyme using fluorescence labels, showing a factor of 10 over the bulk characteristic turnover values.^51^ We have also benchmarked the single-enzyme frequencies (Table 1 ^b^) against the electrocatalytic currents generated in the cyclic voltammetry assays (Figure 3a), yielding frequency values of ∼7500 and ∼85000 min^–1^ (Table 1 ^c^), respectively, in agreement with the single-enzyme frequency values (Table 1 ^b^ and details in SI section 1.5.1). Our nonlabeled, electrically transduced single-protein enzymatic activity also correlates with other single-molecule enzymatic turnover experiments in demonstrating that the overall frequency of an enzymatic reaction is a manifestation of dynamic disorder, where there is a fluctuation in rate constants due to a continuous, stochastic transition between on–off states of enzyme’s activity.^49^ This demonstrates the highly heterogeneous nature of enzymatic catalysis and opens to a detailed study of the complex dynamics in redox enzymatic processes.

### Proposed Mechanisms for the Electrical Transduction of Redox Enzymatic Events

A mechanism has long been hypothesized whereby conductance switching in a single-enzyme junction is used as a means to transduce and *in situ* monitor enzymatic catalysis in real-time.^12^ There is substantial work reporting on conductance switching in single redox-active proteins using both high-resolution EC-STM imaging^11^ and single-protein junction measurements.^7,52^ The telegraph-like fluctuations in the single-molecule current traces contain information about the dynamic changes in the protein redox state. This has been described in detail in previous single-molecule electrical characterization on a model redox Cu Azurin^52^ as well as other synthetic redox molecules,^60^ where the fluctuations between conductance values for the oxidized and reduced states of the molecule were electrochemically driven, and the reduced-to-oxidized population ratio fit a Lorentzian function that accurately predicts the protein redox midpoint potential at the population ratio 1:1^52^. The subtle change in protein conductance occurs when the protein redox state is energetically brought into resonance with the Fermi energies of the two voltage-biased junction metal electrodes via the applied electrochemical “gating” potential. This process has been modeled at length by Kutnesov and Ulstrup,^10,53^ which describes the phenomenon as the opening of an additional two-step sequential electron tunneling channel where the charge transport is mediated by the redox center (Figure 6b). The manifestation of such a charge transport mechanism in P450_cam_ is shown in the potential dependence of the STM image apparent height (Figure 1e), in line with the same analysis in other model redox proteins and redox cofactors.^9,11^ When the potential applied coincides with the protein redox midpoint, the sequential tunneling channel opens, and the protein conductance is enhanced, resulting in a higher apparent height of the protein STM image.^9,11,54^ How does the sequential tunneling scenario relate to the catalytic cycle? In the above examples, the redox resonance conditions leading to sequential tunneling are driven by electrochemically bringing the redox state between the Fermi energy of the two nanojunction electrodes.^7,11,53,54^ In analogy to that, the redox enzyme chemically undergoes subtle redox state changes of the active site cofactor during each catalytic cycle. In the single-protein electrocatalytic experiments, the conductance switching occurs at applied negative overpotentials with respect to the P450_cam_ redox midpoint, which leaves the reduced Fe­(II) redox energy state off-resonance (Figure 6a and c­(i)) leading to a low protein conductance mediated by electron tunneling through the peptide structure of the protein.^55,56,57,58^ During P450_cam_’s catalytic cycle, upon substrate and oxygen binding, the enzyme’s *heme* cofactor momentarily oxidizes from a reduced, oxygen-bound Fe­(II)-Por intermediate at the cathodic applied potentials to an oxidized (high valent) Fe­(IV)/Fe­(III)-Por species that is the active oxidant in the catalytic cycle (Figure 3a and Supplementary Figure S25). This process brings the enzyme's redox state momentarily into resonance and opens the sequential electron tunneling channel that results in a high protein conductance (Figure 6b and c­(ii)). After the substrate enzymatic oxidation takes place, the protein goes back to the reduced state, recovering the low protein conductance value characteristic of off-resonance tunneling (Figure 6c­(iii)). It is important to highlight that the “off-resonant” term here refers purely to nonredox-mediated tunneling for the inactive state, and that the mechanistic details of this process are yet to be understood, since the measured tunneling currents for >2 nm proteins observed in many instances^30b,30c,52,56,57^ cannot be explained by a simple off-resonance quantum tunneling picture. Overall, the flipping between the off- and in-resonance with the protein redox state results in the observed conductance jumps that create the switching trace (Figure 6c­(ii)). The redox resonance condition caused by protein oxidation leads to the opening of an additional sequential tunneling path that is here driven by the enzymatic process, as opposed to the electrochemically induced protein oxidation (see Figure 1e and Supplementary Figure S20). It is important to highlight that our methodology cannot, in its current form, identify particular intermediate states of the catalytic cycle; we observe redox-dependent protein conductance changes when going from a reduced Fe­(II) to an oxidized Fe­(III)-peroxo (compound 0) andFe­(IV)-oxo (compound I) cataytic intermediates of the enzyme without differentiating among specific redox states the active cofactor undergoes during the enzymatic cycle (Supplementary Figure S25). Indeed, it is possible that while compound I needs to be formed for C-H bond activation to occur, the other intermediates formed from the resting state to compound I may differ between electrocatalysis and solution phase biochemical catalysis. We are currently working in chemical routes to allow fixing particular intermediate states to independently characterize their conductance values in the tunneling junction, which will enable their possible identification in the dynamic single-enzyme junction experiments.

**Figure 6:**
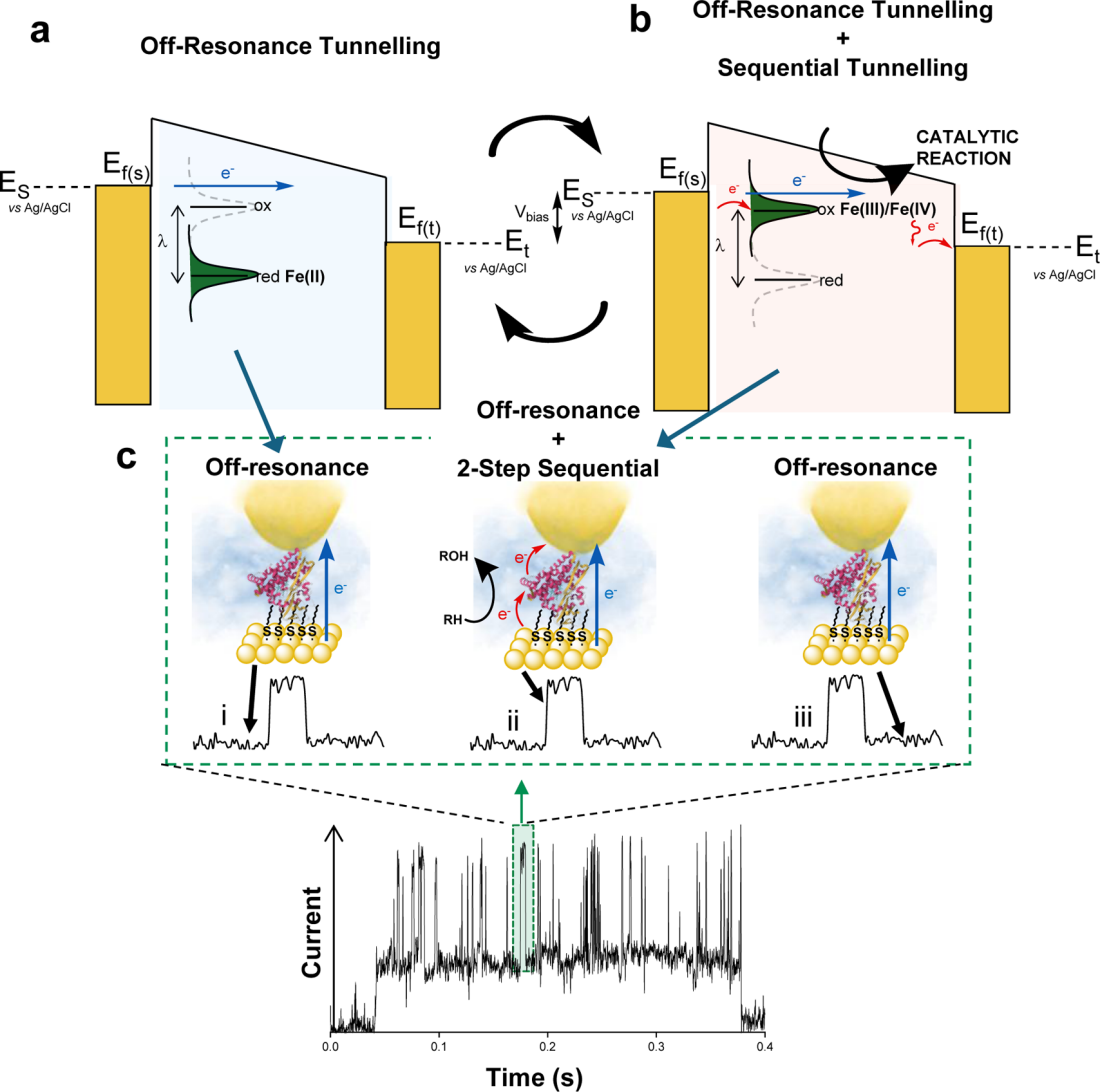
Proposed charge transport mechanism for the observed two-level conductance signal. At applied reducing potentials to the substrate, **a**: the *heme* center is reduced to Fe­(II) and the first level of conductance represents a noncatalytic off-redox resonant tunneling channel, where electrons tunnel through the peptide structure of the enzyme. **b**: During the catalytic reaction, the *heme* center momentarily oxidizes to a high valent Fe­(III)/Fe­(IV) compound I intermadiate state, bringing the redox state into resonance, which opens an additional 2-step sequential tunneling channel and results in the observed second level of conductance. After the reaction is complete, the *heme* center is reduced back to Fe­(II) and the conductance decreases back to the off-redox resonance tunneling channel. c: The sequence off-resonant (reduced) / sequential (oxidised) / off-resonant that makes up the conductance switching event is represented as states (i), (ii) and (iii). E_f (s)_ and E_f (t)_ represents the Fermi level of the substrate and tip, respectively, which are held at electrochemical potentials E_S_ of −0.4 V and −0.3 V, respectively, vs. Ag/AgCl. V_bias_ = E_S_-E_t_ = +0.1 V.

Figure 6 schematically illustrates the mechanism for the momentary, chemically driven opening of the sequential tunneling channel that accounts for the observed two-level conductance switching during the catalytic process. A more in-depth analysis of the potential-dependent STM images in Supplementary Figure S20 shows the same switching behavior when imaging the enzyme under active conditions.

This is a first attempt to monitor real-time single-protein redox enzymatic activity using a non-labeled, all-electrical detection method. Our results are in line with previous reports on single-enzyme activity achieved by exploiting optical detection methods using modified enzymes with various fluorescence labels.^13,14,16^ Our methodology opens to new routes to characterize single-molecule redox enzymatic processes via electrical transduction of individual unmodified enzymes trapped in nanoscale tunneling nanogaps that can be currently mass-produced using present technology.^59^

## Conclusions

Here, we demonstrate real-time electrical transduction of redox enzymatic catalysis on individual nonlabeled enzymes trapped in a nanoscale tunneling gap. We exploit controllable, electrochemical STM-based nanoscale junctions to transiently trap individual P450_cam_ and GR redox enzymes between two electrodes and characterize their charge transport characteristics while the electrocatalytic enzymatic reaction is undergoing. The transient tunneling current flowing across the single-enzyme junction shows a sharp occurrence increase of a characteristic 2-level conductance fluctuations (switching signal) when the enzyme is subjected to the active catalytic conditions, namely, under applied electrochemically reducing potentials and in the presence of the enzymatic substrates and/or redox mediator. The conductance switching appear superimposing the typical single tunneling conductance trace (silent signal) typically observed during the formation of a single-protein junction under noncatalytic conditions. We experimentally demonstrate that the nature of the observed conductance switching events is mainly driven by the dynamic change in the enzyme’s redox state as it undergoes catalytic reaction; during a catalytic cycle, the *heme* active cofactor is momentarily oxidized, thus aligning the enzyme’s redox energy level with the Fermi levels of the junction electrodes and opening an additional redox-mediated sequential tunneling channel that transiently enhances the protein junction conductance. While redox enzymatic catalysis does not involve particularly large conformational changes in the protein structure as compared to nonredox enzymatic processes, e.g., polymerases, we based our redox-dependent conductance fluctuations mechanism on two main observations: (1) the largest conformational changes for P450 occur upon substrate binding; however, we do not observe appreciable changes in conductance values for a P450 protein junction in the absence and presence of the camphor substrate (SI Figure S19). (2) The interpretation of the conductance fluctuations is based on previous work on nonenzymatic redox proteins where a comparable redox-dependent conductance switching induced electrochemically was demonstrated.^52^ We have used two different machine learning-based methods to perform an exhaustive classification of silent and switching signals and extracted the frequencies for the observed switching events. Two different catalytic rates have been extracted from the single-enzyme assays for both enzymes: an *average frequency*, which correlates well with the reported *k*_cat_ determined from standard biochemical assays, and a *single-enzyme frequency*, which quantifies the single-enzyme characteristic turnover and highlights the heterogeneous nature of redox enzymatic processes. These results demonstrate the feasibility of monitoring enzymatic reactions in real-time by electrically transducing catalytic events in a single-protein junction made of an unmodified enzyme, thus providing a new biophysical platform to study single-enzyme redox catalysis and opening to new avenues for label-free detection in biosensing.

## Methods

### Protein Expression and Purification

Recombinant P450cam was produced in *E. coli*Recombinant P450_cam_ was produced as an N-terminal His-Tag fusion protein from E. coli TunerTM (DE3) competent cells (Novogen) transformed with pP450_cam_ (Supplementary Figure S1). Briefly, LB (500 mL, kanamycin 50 μg mL^–1^ ) was innoculated with a fresh overnight culture and incubated to OD 0.8–1.0 (37 °C, 220 rpm). Cells were cooled (15 °C) and induced with IPTG (0.5 mM) following addition of 5-aminolvulinic acid (5 mM) and iron (II) sulphate heptahydrate (1 mM) then incubated for 16 hrs (15 °C, 220 rpm). Cells were harvested by centrifugation (4200 rpm, 20 min, 4 °C). The pellet was resuspended in a minimum volume of Buffer A (20 mM Tris buffer, 100 mM NaCl, 20 mM imidazole, 10% glycerol) with the addition of dithiothreitol (DTT) (final conc. 0.5 mM), Pepstatin A (final conc. 1 μg mL^–1^), DNase A (final conc. 0.2 mg mL^–1^) and Complete protease inhibitor cocktail (1 tablet per 2 L of culture, from Sigma). Cell lysis was carried out on a cell disruptor IXT4A (Constant System LTD) with a pressure of 25 kPa, 4 °C. The lysate was centrifuged (18,000 rpm) for 42 min at 4 °C. The lysate was applied to a 5 mL His-TrapTM Fast Flow nickel affinity column (GE) equilibrated with Buffer A and connected to ÄKTA Pure Chromatography System (GE). His-tagged proteins were eluted with Buffer B (20 mM Tris buffer, 100 mM NaCl, 200 mM imidazole, 10% glycerol). Fractions containing the protein of interest were concentrated to a volume less than 5 mL and injected onto a HiLoad^TM^ 16/600 Superdex 200pg size exclusion chromatography column (GE) equilibrated with Buffer C (20 mM Tris buffer, 100 mM NaCl, 10% glycerol). The purification was monitored at 280 and 420 nm (Figure S2). The proteins were eluted with Buffer C, and fractions with *heme*-incorporated protein were analyzed by SDS-PAGE gel (Figure S2) and were then concentrated and mixed with an equal volume of Buffer D (20 mM Tris buffer, 100 mM NaCl, 30% glycerol and stored in aliquotes at -80 °C). Glutathione reductase (GR) from *S. cerevisiae* was purchased from Sigma-Aldrich.

### Preparation of Au(111)/Enzyme Interfaces

Au­(111) (MaTecK) was placed in a piranha solution (30% w/w hydrogen peroxide to concentrated sulfuric acid in a 1:3 ratio) for 4 min then rinsed with Milli-Q water. Next, the electrode was electrochemically oxidized in 0.1 M sulfuric acid for ∼1.5 min and immersed in 0.1 M hydrochloric acid to dissolve the oxide layer for 20 min. Finally, the electrode was annealed in a hydrogen flame for 6 min and cooled under nitrogen gas for 2 min. Clean Au(111) substrates were placed in 1-octanethiol (1 mM) solutions using ethanol as a solvent for 18 h. Then the electrodes were thoroughly rinsed with ethanol, then Milli-Q water and dried under nitrogen gas. P450_cam_/GR (20 μM) was incubated on the 1-octanethiol-functionalized Au(111) for 2 h at 4 °C, then rinsed with Milli-Q water.

### Cyclic Voltammetry and Electrocatalysis

Measurements were carried out using a Metrohm-Autolab (The Netherlands) run on the NOVA software using a three-electrode configuration: 1.6 mm diameter polycrystalline Au working electrode, a long platinum wire counter electrode, and Ag/AgCl (3 M NaCl) reference electrode.

**P450**_**cam**_: The electrochemical/electrocatalytic measurements were conducted in an electrolyte containing P450_cam_ (20 μM) and D-camphor (1 mM) in phosphate buffer (0.1 M, pH 7) as the supporting electrolyte. The potential voltage applied to the cell was swept from +0.1 V to −0.50 at 0.005 V s^–1^. Control experiments were carried out in the absence of both P450_cam_ and camphor, and in the absence of camphor only.

**GR**: The electrochemical/electrocatalytic measurements were conducted in an electrolyte containing glutathione reductase (4.4 μM), methyl viologen dichloride (1 mM), and GSSG (10 mM) in Tris-KCl (0.1 M, pH 7) as the supporting electrolyte. The potential voltage applied to the cell was swept from 0 V to −0.85 V at 0.01 V s^–1^. Control experiments were carried out in the absence of GR and in the absence of both GR and GSSG.

### EC-STM Imaging and Nanogap Formation

All electrochemical STM experiments were conducted in a Scanning Tunnelling Microscope (Bruker, USA) controlled by a Nanoscope V electronics run by Nanoscope software v9.7. Current–time measurements were conducted using a LabVIEW interface controlled by homemade LabVIEW codes. Electrochemical STM cells were cleaned using piranha before use. Surface-absorbed enzymes on the thiol-functionalized Au(111) substrates were mounted into a clean Teflon cell. Phosphate buffer (0.1 M, pH 7) for P450_cam_ or Tris-KCl (0.1 M, pH 7) for GR was used as the supporting electrolytes. All Au tips were electrochemically etched in 50:50 ethanol:HCl, functionalized with 1-octanethiol (1 mM, 1 h), coated with Apiezon wax, and placed into the tip holder of the STM scanner equipped with a homemade 10 nA V^–1^ current amplifier. The scanner and cell were mounted onto the STM with Au(111), at Au STM probe, as the working electrode, Pt acts as the counter electrode and a miniaturized Ag/AgCl (3 M KCl) as the reference electrode.

Electrochemical STM images were obtained by scanning the tip in the XY plane of the substrates using a sample-to-tip bias voltage of +0.1 V, a current set point of 0.1–0.05 nA, and electrochemical potential of +0.1 V. P450–C8–Au was imaged in the presence and absence of camphor at different electrochemical potentials between −0.1 V and −0.45 V. The current-height of the individual P450_cam_ enzymes was analyzed from electrochemical STM images using Gwyddion software.

Electrochemical STM-based nanogap measurements were carried out using a sample-to-tip bias voltage of +0.1 V and a current set point of ∼40 pA. For P450_cam_ junctions, D-camphor (250 μM) was added to the electrochemical cell, and the sample potential was held at +0.1 V and −0.4 V. For GR junctions, methyl viologen dichloride (157 μM) and GSSG (1527 μM) were added to the cell, respectively, and the sample potential was held at +0.1 V and −0.5 V. In all cases, the STM probe potential was always +100 mV with respect to the sample potential. Thousands of blinks were collected for each experimental condition throughout a large number of independent single-protein experiments conducted on different days and on different, freshly prepared samples and electrochemical setups. Categorization of the blinks is described in the Supporting Information section 3.1.

## Supplementary Material



## Notes

Section 1: General methods. Section 2: Single-enzyme conductance analysis. Section 3: Categorization and analysis of blinking traces. Section 4: Enzyme catalytic traces and Protein Electrostatics. Section 5: Steady-State Kinetics Supplementary Figures S1–28 (PDF)
