# Supplementary material for: Quantifying the Characteristic Turnover Frequency of an Individual Redox Enzyme via Real-Time Electrical Transduction of Catalytic Events in a Label-Free Single-Protein Junction
Source: J Am Chem Soc. 2026 Jul 31;148(31):33004–16. doi: 10.1021/jacs.5c23035 (PMC13474935; doi:10.1021/jacs.5c23035)
Supplement: Supplementary file 1 [file ja5c23035_si_001.pdf]

Quantifying the Characteristic Turnover Frequency of an Individual Redox Enzyme via Real-Time Electrical Transduction of Catalytic Events in a Label-free Single-Protein Junction

Tracy Quynh Ha[1], Albert C. Aragonès[2*], Qiankun Wang[1], Desmond Koomson[1], Nashili Kibria[1], Sofia Margiola[1], Yongchan Jeong[1], Jhanelle White[1], Kavita Garg[1,3], Jessica Peate[1], Alex P.S. Brogan[1], Leigh Aldous[1,4], Sarah M. Barry[1*], Ismael Díez-Pérez[1*]

[1]Department of Chemistry, Faculty of Natural, Mathematical & Engineering Sciences, King's College London, Britannia House, 7 Trinity Street, London SE1 1DB, UK.

[2]Department of Physical Chemistry, Max Planck Institute Partner Group, University of Barcelona, Martí i Franquès 1, 08028, Barcelona, Spain.

[3]Department of Chemistry, School of Physical Sciences, DIT University, Dehradun-248001.

[4]Department of Chemical Engineering, National Taiwan University, Roosevelt Road, Taipei 106, Taiwan.

***e-mail**: ismael.diez_perez@kcl.ac.uk, sarah.barry@kcl.ac.uk, acortijos@ub.edu

Supplementary Information

Outline

TABLE OF CONTENTS

1. General Methods 3

1.1. P450cam production and purification 3

1.2. Spectrophotometric Characterisation 5

1.2.1 Determination of heme cofactor content in P450cam by pyridine hemochromagen 6

1.2.2 Determination of flavin cofactor content in GR by heat denaturation 6

1.3. Biochemical assay analysed by GC-MS to determine activity of P450cam 8

1.4. Substrate concentration-dependent biochemical assay 9

1.5. Spectrophotometric NADPH consumption assay for GR 15

1.5.1 Electrocatalysis of solution-phase GR and p450 by Cyclic voltammetry 17

1.5.2. UV-vis absorption spectroscopy to monitor NADPH consumption by GR after solution-phase electrocatalysis 18

1.5.3. UV-vis absorption spectroscopy to monitor NADPH consumption by GR after surface-adsorbed electrocatalysis 19

1.6. Chronoamperometric Electrocatalysis of solution phase $P450_{cam}$ and surface-adsorbed P450cam-C8-Au 21

1.7. Calibration of nanoscale gap distance for the P450-C8-Au and GR-C8-Au interfaces 24

1.8. AFM Imaging .... Error! Bookmark not defined.

1.9. Height and diameter analysis of STM/AFM images 25

1.10. AFM scratching of 1-octanethiol-Au 28

1.11. Electrochemical desorption of 1-octanethiol 29

2. Single enzyme conductance analysis 30

2.1 1D conductance histograms of surface-adsorbed $P450_{cam}$ and GR under different catalytic conditions 30

2.2 STM apparent heights of $p450_{cam}$ under the different catalytic conditions 31

3. Categorisation and analysis of blinking traces 32

3.1. Automatic data sorting 32

3.2. Frequency analysis 33

4. Enzyme catalytic cycles and Protein Electrostatics 355

5. Steady-State Kinetics 38

References: 39

1. General Methods

1.1. P450cam production and purification

The $P450_{cam}$ encoding plasmid (**Fig. S1**, pP450cam was a gift from Teruyuki Nagamune (Addgene plasmid #85085; http://n2t.net / addgene:85085; RRID:Addgene_85085)[1] was used to transform TunerTM (DE3) competent cells (Novogen) according to manufacturer's instructions. Single colonies were used to inoculate 10 mL of LB liquid media (50 µg mL^{-1} kanamycin) and incubated overnight at 37°C with shaking (220 rpm). 3 mL of the overnight culture was added to 500 mL of pre-warmed LB media (50 µg mL^{-1} kanamycin) and incubated further at 37 °C with shaking (220 rpm).

Cultures (OD_{600} 0.8-1) were cooled to 15°C and induced with isopropyl-β-D-thiogalactopyranoside (IPTG) (0.5 mM final conc.). Filter sterilised solutions of 5-aminolevulinic acid (final conc. 5 mM) and iron (**II**) sulphate heptahydrate (final conc. 1 mM) were added and the cultures were incubated overnight at 15°C with shaking (220 rpm).

Cells were harvested by centrifugation (4200 rpm, 20 min, 4°C). The pellet was resuspended in a minimum volume of Buffer A (20 mM Tris buffer, 100 mM NaCl, 20 mM imidazole, 10% glycerol) with the addition of dithiothreitol (DTT) (final conc. 0.5 mM), Pepstatin A (final conc. 1 µg mL^{-1} per), DNAse A (final conc. 0.2 mg mL^{-1}) and Complete™ protease inhibitor cocktail (1 tablet per 2 L of culture from Sigma). Cell lysis was carried out on a cell disruptor IXT4A (Constant System LTD) with a pressure of 25 kPa, 4 °C. The lysate was centrifuged (18,000 rpm) for 42 min at 4°C. The lysate was applied to a 5 mL His-Trap™ Fast Flow nickel affinity column (GE) equilibrated with Buffer A and connected to ÄKTA Pure Chromatography System (GE). His tagged proteins were eluted with Buffer B (20 mM Tris buffer, 100 mM NaCl, 200 mM imidazole, 10% glycerol). Fractions containing the protein of interest were concentrated to a volume less than 5 mL and injected onto a HiLoadTM 16/600 Superdex 200 pg size exclusion chromatography column (GE) equilibrated with Buffer C (20 mM Tris buffer, 100 mM NaCl, 10% glycerol). The purification was monitored at 280 nm and 420 nm (**Fig. S2**). The proteins were eluted with Buffer C and fractions with *heme* incorporated protein were analysed by SDS-PAGE gel (**Fig. S2**) and were then concentrated and mixed with an equal volume of Buffer D (20 mM Tris buffer, 100 mM NaCl, 30% glycerol) and divided into 10 - 40 µL aliquots, flash frozen and stored at -80°C.

a MGSSHHHHHHSSGLVPR/GSHMTTETIQSNANLAPLPPHVPEHLVFDFDMYNPSNLSAGVQEAWAVLQESNVP
DLVWTRCNGGHWIATRGQLIREAYEDYRHFSSECPFIPREAGEAYDFIPTSMDPPEQRQFRALANQVVGMPVVD
KLENRIQELACSLIESLRPQGQCNFTEDYAEPFPIRIFMLLAGLPEEDIPHLKYLTDQMTRPDGSMTFAEAKEALYDYL
IPIIEQRRQKPGTDAISIVANGQVNGRPITSDEAKRMCGLLLVGGLDTVVNFLSFSMEFLAKSPEHRQELIERPERIPA
ACEELLRRFSLVADGRILTSDYEFHGVQLKKGDQILLPQMLSGLDERENACPMHVDFSRQKVSHTTFGHGSHLCLG
QHLARREIIVTLKEWLTRIPDFSIAPGAQIQHKSGIVSGVQALPLVWDPATTKAV

His tag

Thrombin site

/ Cleavage site

P450cam

b

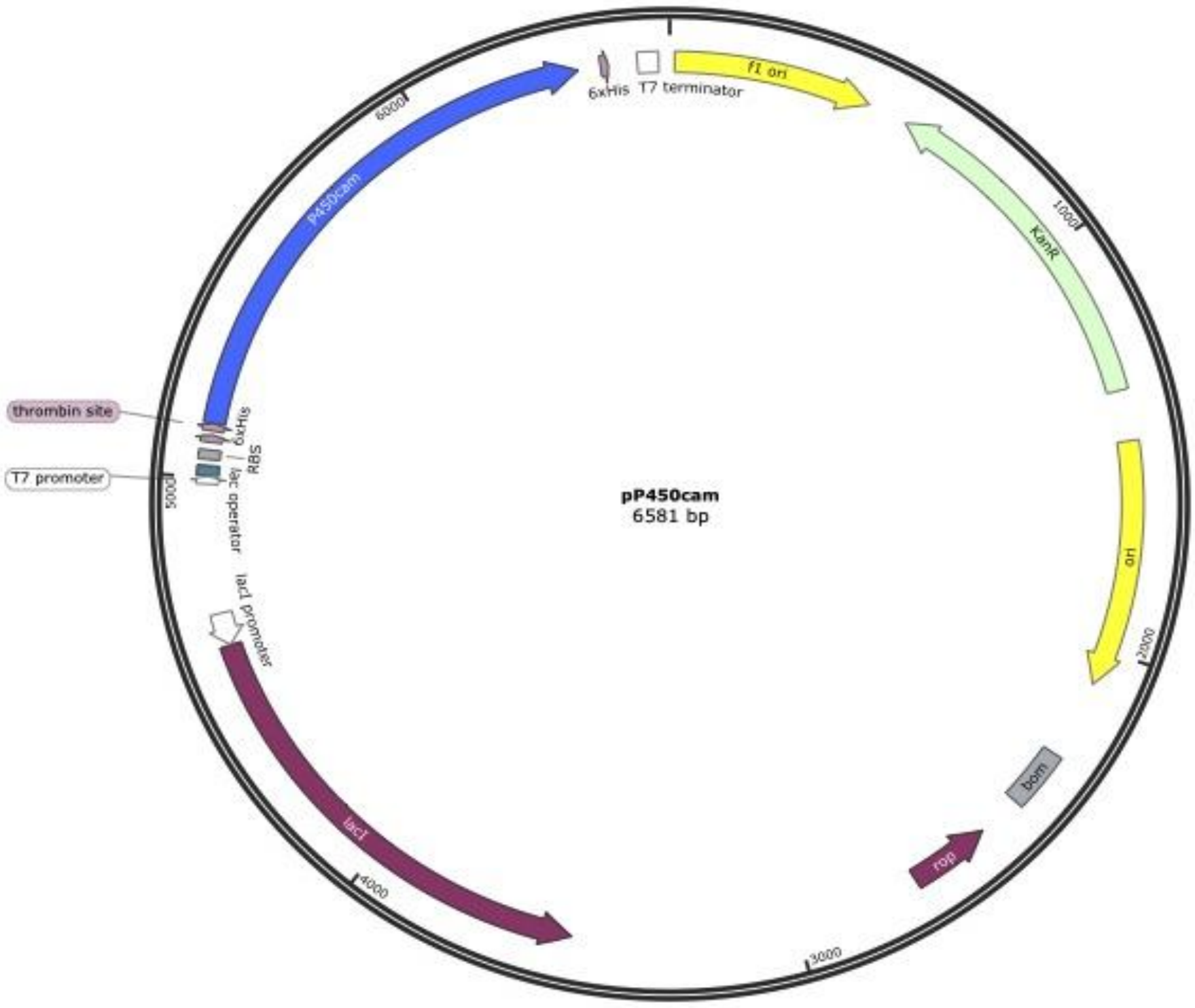

Supplementary Figure S1: a) recombinant His$_6$-P450cam protein sequence and b) plasmid map pET28b-His$_6$-P450cam. The map shows the relative location of the gene of interest encoding P450cam, and structural features, for example, a kanamycin resistance gene (KanR), used for selection. The plasmid incorporates a N-terminal 6x polyhistidine tag for protein purification by Nickel affinity chromatography, a thrombin cleavage site and T7 promoter and lac repressor protein (lacI) for IPTG-induced gene

1.2. Spectrophotometric Characterisation

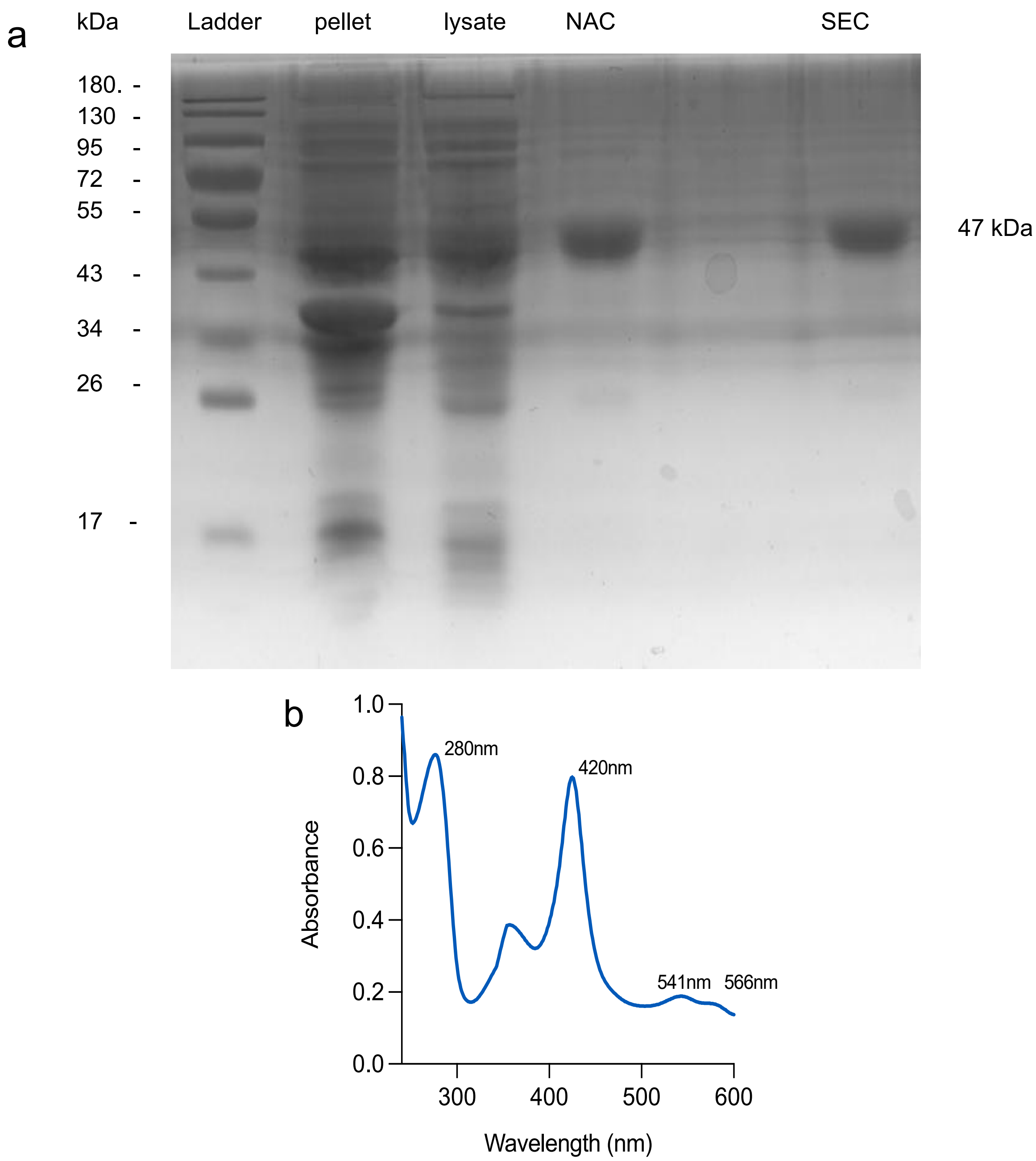

Supplementary Figure S2: Overproduction and purification of $P450_{cam}$. a) SDS PAGE (12%) analysis of His_6-$P450_{cam}$ (47 kDa) after centrifugation (pellet), cell lysis (lysate), nickel affinity (NAC) and size exclusion chromatography (SEC), b) UV-visible spectrum of purified $P450_{cam}$ (20µM, phosphate buffer, 0.1 M, pH 7).

1.2.1 Determination of heme cofactor content in P450cam by pyridine hemochromagen

The UV-visible spectrometer was blanked with a Solution I (0.2 M NaOH, 40% (v/v) pyridine, 500 μM potassium ferricyanide) and Buffer D in a 1:1 ratio. A sample of Solution I and purified P450cam (50 μM) with a 1:1 ratio was measured (oxidised). A spatula of sodium dithionite was stirred into the sample and immediately scanned (reduced). The absorbances were obtained to calculate the concentration of *heme* in the protein using Beer Lambert's Law: $A = \varepsilon c l$ (**Fig. S3a**). The difference between $\Delta_{557\text{-}575}$ of the reduced and oxidised state were used to calculate using the extinction molar coefficient ε = 32.4 $mM^{-1}\,cm^{-1}$ [2].

1.2.2 Determination of flavin cofactor content in GR by heat denaturation

The spectrometer was blanked with the buffer 10 mM Tris-HCl buffer (pH 8). The absorbance spectrum of GR as measured (before heating). The sample was heated at 100°C for 10 minutes, and then cooled. The denatured protein was removed by centrifugation (10 minutes, 13,000 rpm, 4°C). The supernatant was recovered, and the absorbance spectrum was recorded (after heating). The percentage of FAD was obtained using the difference in absorbance of the protein before and after heating at 280 nm to obtain the concentration of protein that denatured (**Fig. S3b**) using the extinction molar coefficient ε = 50.5 $mM^{-1}cm^{-1}$ (Expasy ID: P41921), and the absorbance at 455 nm after heat using the extinction molar coefficient ε =11.3 $mM^{-1}cm^{-1}$ [3].

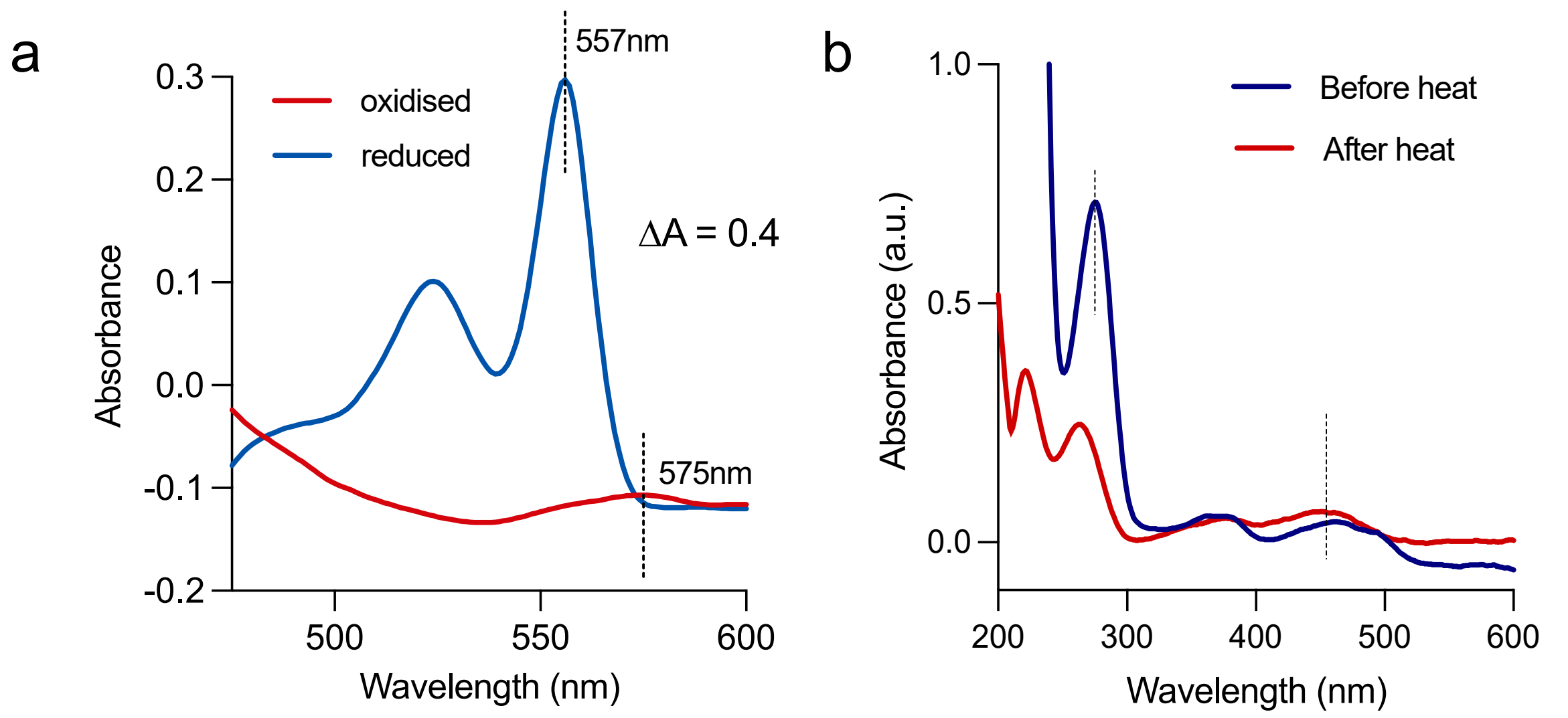

Supplementary Figure S3: Spectrophotometric determination of cofactor content in enzymes. a) *Heme* content of $P450_{cam}$ by pyridine hemochromogen [2]. The vertical dotted lines indicate 557nm and 575nm. There is a ΔA of 0.4 indicating a heme content of 24%. b) FAD content of GR by heat denaturation. Vertical lines indicate 280 nm and 455 nm. GR was purchased from Sigma Aldrich (G3664).

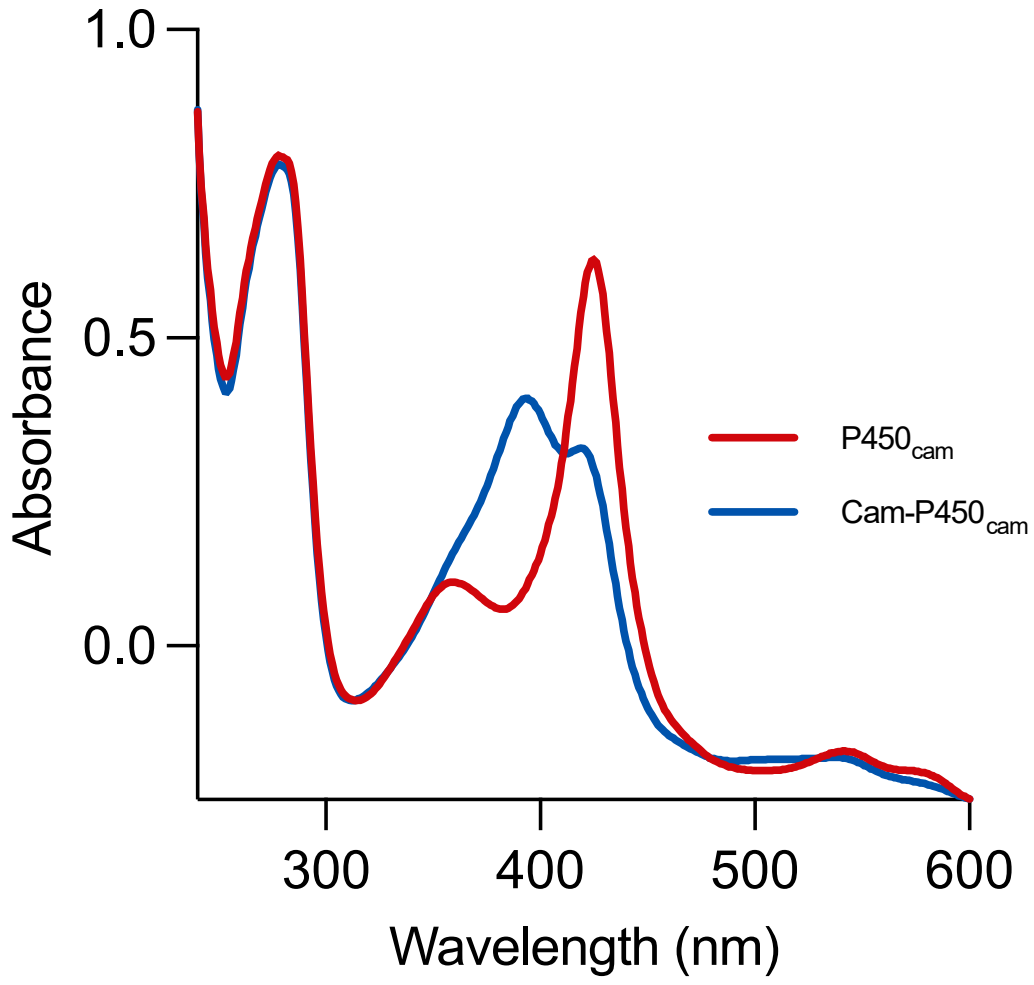

Supplementary Figure S4: Changes in UV-*vis* spectrum for substrate binding of $P450_{cam}$ where the substrate-free $P450_{cam}$ ($P450_{cam}$) shows a characteristic λ_{max} absorbance at 420 nm for the low spin iron in the *heme* cofactor (0.1 M phosphate buffer, pH 7, 20 μM), which shifts to 390nm for substrate-adsorbed high spin iron $P450_{cam}$ (cam-$P450_{cam}$) (addition of camphor dissolved in 2% DMSO was added to the protein solution, final concentration 50 μM)[2,3].

1.3. BIOCHEMICAL ASSAY ANALYSED BY GC-MS TO DETERMINE ACTIVITY OF P450CAM

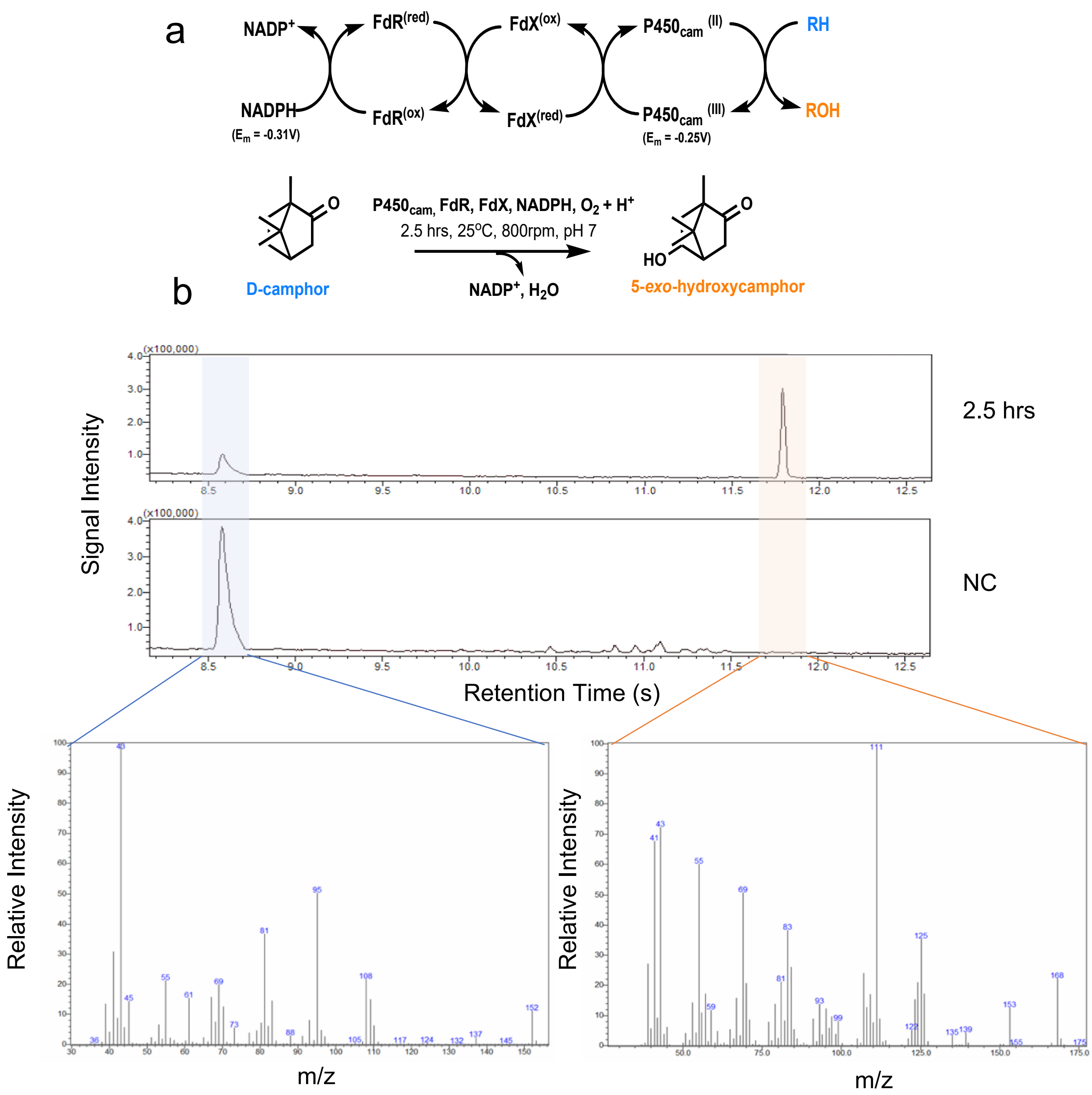

Supplementary Figure S5: GC-MS analysis of $P450_{cam}$ activity assay. a: catalytic reaction scheme for the conversion of camphor to 5-*exo*-hydroxycamphor. Middle: GC-MS traces of 20 µM $P450_{cam}$ incubated with 50 µM camphor (blue) for 2.5 hrs at 25°C [2.5hrs] and corresponding negative control [NC]. The product is highlighted in orange. b: corresponding mass spectra of camphor (m/z 152, RT 8.5) and 5-*exo*-hydroxycamphor (m/z 168, RT 11.8) ions. The fragmentation patterns for both molecules match those previously reported [4] [5].

$P450_{cam}$ (20 μM in 0.1 M phosphate buffer pH 7) was added to ferredoxin (final conc. 40 μM) and ferredoxin reductase (final conc. 0.08 U) from *Spinancia oleracea* (Sigma), D-camphor (final conc. 50 μM) from a stock (10 mM, 2% DMSO) was added to a final volume of 100 μL (0.1 M phosphate buffer, pH 7), with NADPH (final conc. 1 mM) added last to initiate the reaction. Reactions were incubated for 2.5 hours at 25°C with shaking (800 rpm) in a Thermomixer (Eppendorf). Negative controls were carried out as above but $P450_{cam}$ was heat inactivated (>100°C, 15 minutes) prior to addition. After incubation, reactions were extracted with ethyl acetate (2 x 100 μL) and centrifuged (Thermofischer) at 5000 rpm for 5 minutes between each extract. The extracts were combined and analysed by Gas Chromatography Mass Spectrometry (Shimadzu, GCMS -QP2020 NX) fitted with Shimadzu-5 MS column and compared to literature values (**Fig. S5**) [4] [5]. The oven temperature was held at 60°C for 2 min then increased to 240°C for over 18 min, and held at 240°C for 4 min. Helium was used as a carrier gas.

1.4. Substrate concentration-dependent biochemical assay

The biochemical assay outlined in **section 1.3** was repeated for varying concentrations of camphor and reaction times (**Fig. S6-8**):

	P450 (μM)	Ferredoxin (c)	Ferredoxin Reductase (U)	NADPH (mM)	D-camphor (mM)	Reaction time (hr)
1	20	40	0.08	1	0.1	2.5
2	20	40	0.08	1	0.1	4
3	20	40	0.08	1	0.1	7
3	20	40	0.08	1	0.1	16
3	20	40	0.08	1	1	2.5
3	20	40	0.08	1	1	4
3	20	40	0.08	1	1	7
3	20	40	0.08	1	1	16
3	20	40	0.08	1	10	2.5
3	20	40	0.08	1	10	4
3	20	40	0.08	1	10	7
3	20	40	0.08	1	10	16

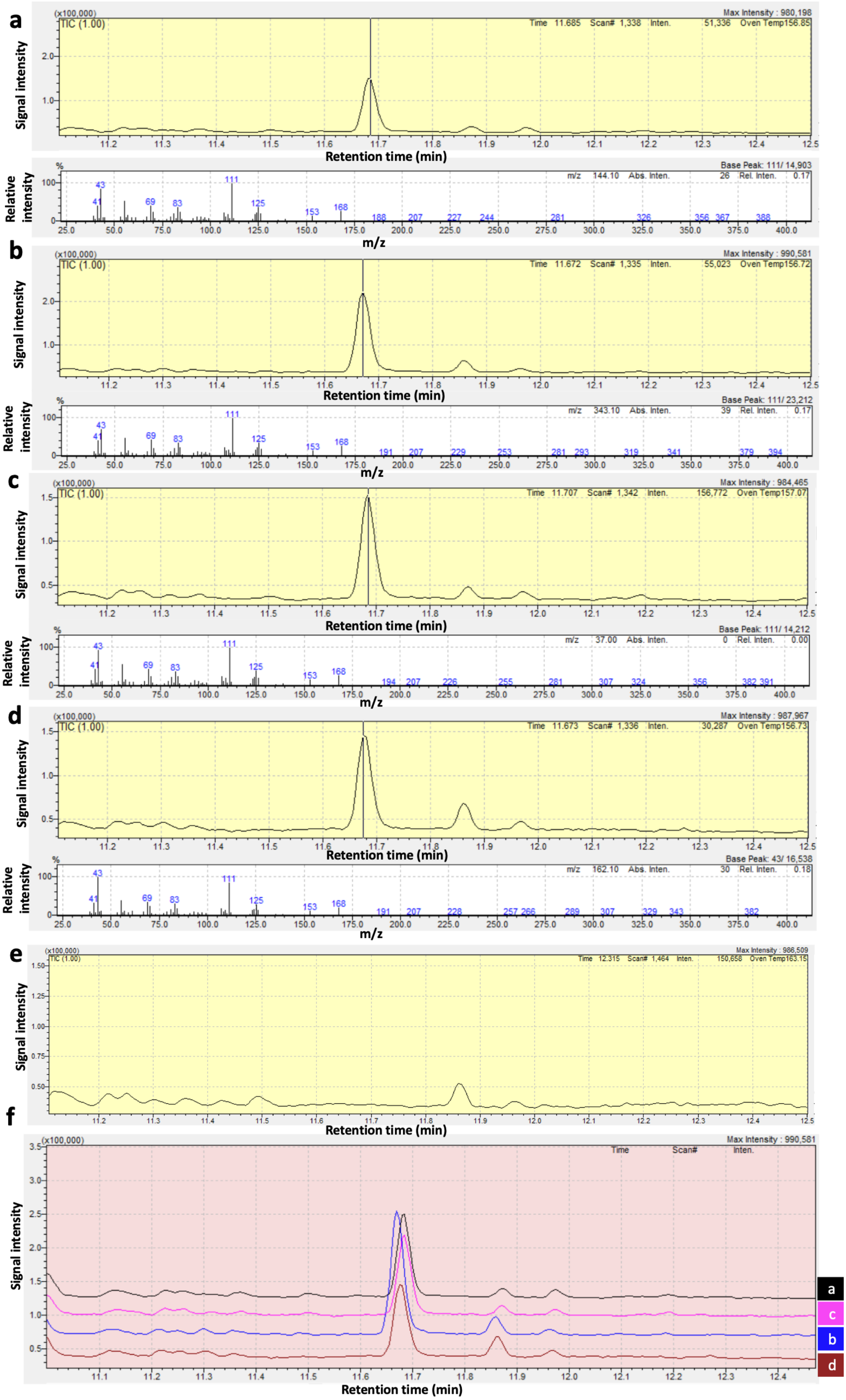
a
(x100,000)
TIC (1.00)
Max Intensity : 980,198
Time 11.685 Scan# 1,338 Inten. 51,336 Oven Temp156.85
Signal intensity
Retention time (min)
Base Peak: 111/ 14,903
m/z 144.10 Abs. Inten. 26 Rel. Inten. 0.17
Relative intensity
43 41 69 83 111 125 153 168 188 207 227 244 281 326 356 367 388
m/z
b
(x100,000)
TIC (1.00)
Max Intensity : 990,581
Time 11.672 Scan# 1,335 Inten. 55,023 Oven Temp156.72
Signal intensity
Retention time (min)
Base Peak: 111/ 23,212
m/z 343.10 Abs. Inten. 39 Rel. Inten. 0.17
Relative intensity
43 41 69 83 111 125 153 168 191 207 229 253 281 293 319 341 379 394
m/z
c
(x100,000)
TIC (1.00)
Max Intensity : 984,465
Time 11.707 Scan# 1,342 Inten. 156,772 Oven Temp157.07
Signal intensity
Retention time (min)
Base Peak: 111/ 14,212
m/z 37.00 Abs. Inten. 0 Rel. Inten. 0.00
Relative intensity
43 41 69 83 111 125 153 168 194 207 226 255 281 307 324 356 382 391
m/z
d
(x100,000)
TIC (1.00)
Max Intensity : 987,967
Time 11.673 Scan# 1,336 Inten. 30,287 Oven Temp156.73
Signal intensity
Retention time (min)
Base Peak: 43/ 16,538
m/z 162.10 Abs. Inten. 30 Rel. Inten. 0.18
Relative intensity
43 41 69 83 111 125 153 168 191 207 228 257 266 289 307 329 343 382
m/z
e
(x100,000)
TIC (1.00)
Max Intensity : 986,509
Time 12.315 Scan# 1,464 Inten. 150,658 Oven Temp163.15
Signal intensity
Retention time (min)
f
(x100,000)
Max Intensity : 990,581
Time Scan# Inten.
Signal intensity
Retention time (min)
a
c
b
d

Supplementary Figure S6: GC-MS analysis of the biochemical activity assay of P450cam with 100 μM camphor at different time points. Each panel shows the chromatogram and corresponding mass spectrum in the elution region of 5-exo-hydroxycamphor (~11.6 min) for the following time points: (a) 2.5 hours, (b) 4 hours, (c) 7 hours, (d) 16 hours, along with (e) a negative control, and (f) an overlay of all time points. The biochemical assay was conducted with P450cam (50 μM) incubated with camphor (100 μM), ferredoxin (FdX, 0.08 g/L), ferredoxin reductase (FdR, 0.08 U), and NADPH (1 mM) in phosphate buffer (0.1 M, pH 7) at 25°C, with reactions sampled at the indicated time points. Notably, two peaks at ~11.85 min and ~11.98 min are also present in the negative control.

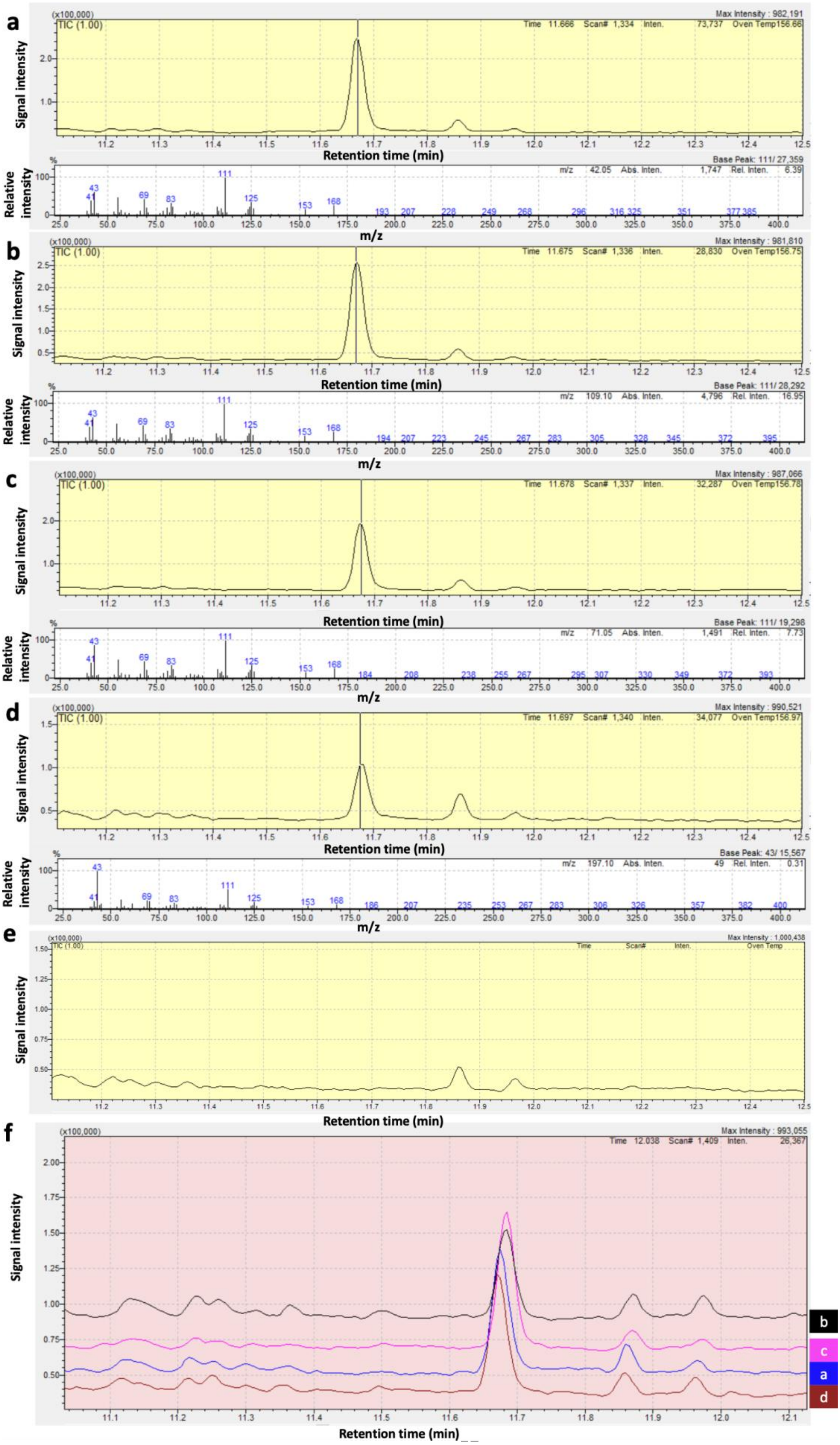
a
(x100,000)
TIC (1.00)
Max Intensity : 982,191
Time 11.666 Scan# 1,334 Inten. 73,737 Oven Temp156.66
Signal intensity
Retention time (min)
Base Peak: 111/ 27,359
m/z 42.05 Abs. Inten. 1,747 Rel. Inten. 6.39
Relative intensity
m/z
b
(x100,000)
TIC (1.00)
Max Intensity : 981,810
Time 11.675 Scan# 1,336 Inten. 28,830 Oven Temp156.75
Signal intensity
Retention time (min)
Base Peak: 111/ 28,292
m/z 109.10 Abs. Inten. 4,796 Rel. Inten. 16.95
Relative intensity
m/z
c
(x100,000)
TIC (1.00)
Max Intensity : 987,066
Time 11.678 Scan# 1,337 Inten. 32,287 Oven Temp156.78
Signal intensity
Retention time (min)
Base Peak: 111/ 19,298
m/z 71.05 Abs. Inten. 1,491 Rel. Inten. 7.73
Relative intensity
m/z
d
(x100,000)
TIC (1.00)
Max Intensity : 990,521
Time 11.697 Scan# 1,340 Inten. 34,077 Oven Temp156.97
Signal intensity
Retention time (min)
Base Peak: 43/ 15,567
m/z 197.10 Abs. Inten. 49 Rel. Inten. 0.31
Relative intensity
m/z
e
(x100,000)
TIC (1.00)
Max Intensity : 1,000,438
Signal intensity
Retention time (min)
f
(x100,000)
Max Intensity : 993,055
Time 12.038 Scan# 1,409 Inten. 26,367
Signal intensity
Retention time (min)
b
c
a
d

Supplementary Figure S7: GC-MS analysis of the biochemical activity assay of P450cam with 1 mM camphor at different time points. Each panel shows the chromatogram and corresponding mass spectrum in the elution region of 5-exo-hydroxycamphor (~11.6 min) for the following time points: (a) 2.5 hours, (b) 4 hours, (c) 7 hours, (d) 16 hours, along with (e) a negative control, and (f) an overlay of all time points. The biochemical assay was conducted with P450cam (50 μM) incubated with camphor (100 μM), ferredoxin (FdX, 0.08 g/L), ferredoxin reductase (FdR, 0.08 U), and NADPH (1 mM) in phosphate buffer (0.1 M, pH 7) at 25°C, with reactions sampled at the indicated time points. Notably, two peaks at ~11.85 min and ~11.98 min are also present in the negative control.

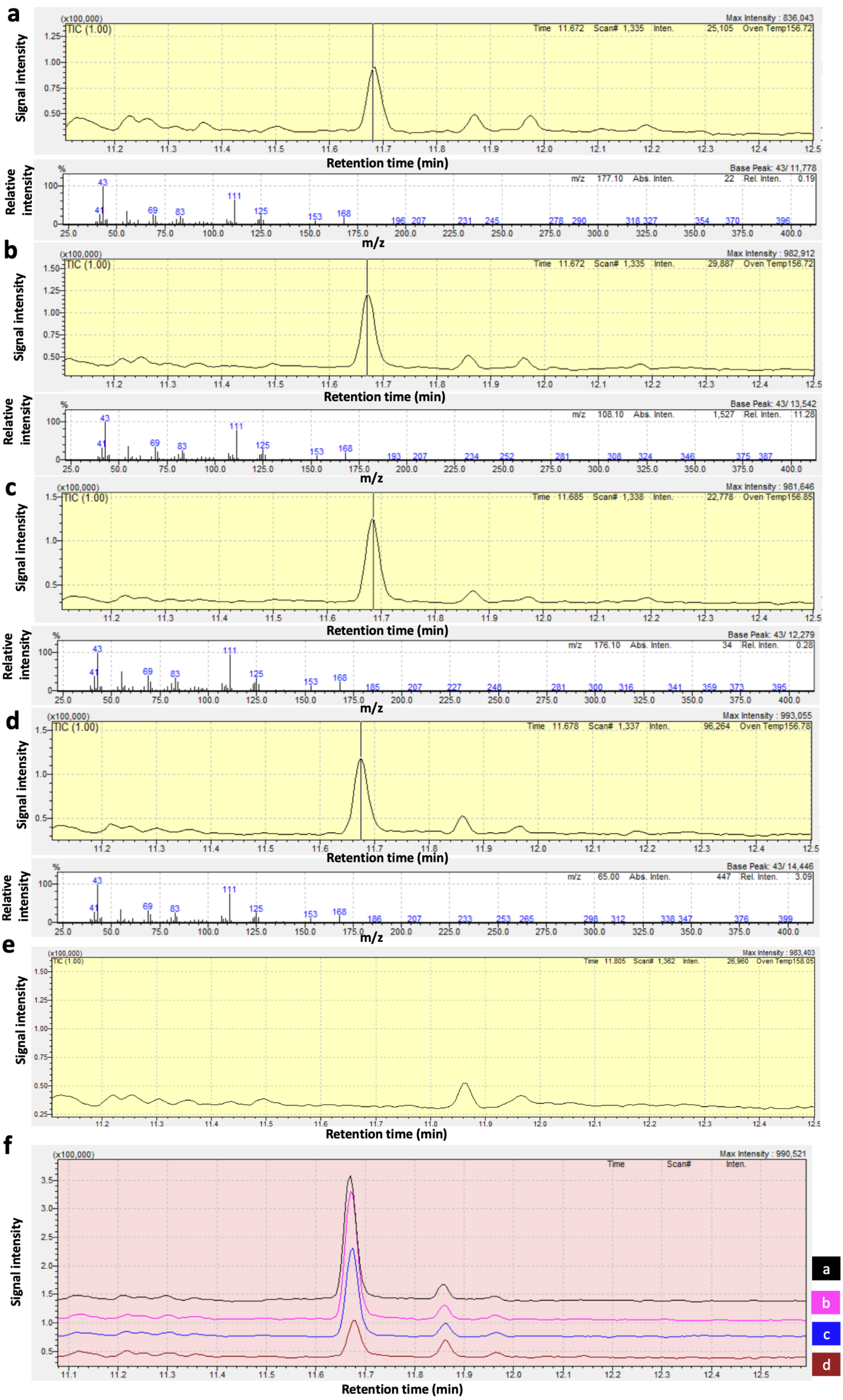

a
(x100,000)
TIC (1.00)
Time 11.672 Scan# 1,335 Inten. 25,105 Oven Temp156.72
Max Intensity : 836,043
Signal intensity
Retention time (min)
Relative intensity
Base Peak: 43/ 11,778
m/z 177.10 Abs. Inten. 22 Rel. Inten. 0.19
43
41
69
83
111
125
153
168
m/z
b
(x100,000)
TIC (1.00)
Time 11.672 Scan# 1,335 Inten. 29,887 Oven Temp156.72
Max Intensity : 982,912
Signal intensity
Retention time (min)
Relative intensity
Base Peak: 43/ 13,542
m/z 108.10 Abs. Inten. 1,527 Rel. Inten. 11.28
43
41
69
83
111
125
153
168
m/z
c
(x100,000)
TIC (1.00)
Time 11.685 Scan# 1,338 Inten. 22,778 Oven Temp156.85
Max Intensity : 981,646
Signal intensity
Retention time (min)
Relative intensity
Base Peak: 43/ 12,279
m/z 176.10 Abs. Inten. 34 Rel. Inten. 0.28
43
41
69
83
111
125
153
168
m/z
d
(x100,000)
TIC (1.00)
Time 11.678 Scan# 1,337 Inten. 96,264 Oven Temp156.78
Max Intensity : 993,055
Signal intensity
Retention time (min)
Relative intensity
Base Peak: 43/ 14,446
m/z 65.00 Abs. Inten. 447 Rel. Inten. 3.09
43
41
69
83
111
125
153
168
m/z
e
(x100,000)
TIC (1.00)
Time 11.805 Scan# 1,362 Inten. 26,960 Oven Temp158.05
Max Intensity : 983,403
Signal intensity
Retention time (min)
f
(x100,000)
Max Intensity : 990,521
Time
Scan#
Inten.
Signal intensity
Retention time (min)
a
b
c
d

Supplementary Figure S8: GC-MS analysis of the biochemical activity assay of P450cam with 10 mM camphor at different time points. Each panel shows the chromatogram and corresponding mass spectrum in the elution region of 5-exo-hydroxycamphor (~11.6 min) for the following time points: (a) 2.5 hours, (b) 4 hours, (c) 7 hours, (d) 16 hours, along with (e) a negative control, and (f) an overlay of all time points. The biochemical assay was conducted with P450cam (50 µM) incubated with camphor (100 µM), ferredoxin (FdX, 0.08 g/L), ferredoxin reductase (FdR, 0.08 U), and NADPH (1 mM) in phosphate buffer (0.1 M, pH 7) at 25°C, with reactions sampled at the indicated time points. Notably, two peaks at ~11.85 min and ~11.98 min are also present in the negative control.

1.5. Spectrophotometric NADPH consumption assay for GR

GR from *S. cerevisiae* was purchased from Sigma Aldrich (G3664). The electrocatalytic activity of solution-phase GR and surface-adsorbed GR-C8-Au (**Fig. S9**) was confirmed by extracting their reaction solution after conducting cyclic voltammetry and chronoamperometry, respectively, which is used to conduct a biochemical reaction using NADPH for the remaining GSSG. The NADPH consumption of the biochemical reaction is monitored by UV-vis spectroscopy (**Fig. S11**). To compare with the experimental conditions where GR was present, a control electrocatalytic reaction was carried out in the absence of GR. Subsequently, a portion of this reaction mixture for the biochemical reaction was analysed. **Table 1** displays the required components and reaction conditions in each electrocatalytic experiment (EC reaction), from which samples were taken and directly used in the biochemical experiment (biochemical reaction). The absorbance of NADPH is observed at 340 nm, and the difference between the experimental and control is the consumption of GSSG during the electrocatalysis, and therefore the concentration of GSH produced. The concentration of GSH was calculated in a 1:2 ratio using Beer Lambert's law and the molar extinction coefficient of NADPH of 6220 M^{-1} cm^{-1} [6]. The methodologies for the two echem methods, cyclic voltammetry and chronoamperometry, and their corresponding NADPH consumption assay is outlined in **section 1.5.1 - 1.5.2** and **section 1.5.3 -1.5.4,** respectively.

Echem method	Cell Potential (V)	Catalytic Experiments		Controls	
		EC reaction	Biochemical analysis (Fig. S11)	EC reaction	Biochemical analysis (Fig. S11)
Cyclic voltammetry	0 to -0.85	MV (1 mM), GSSG (10 mM) GR (4.4 μM)	NADPH (1 mM), EC reaction mixture (10%)	MV (1 mM) GSSG (10 mM)	NADPH (1 mM), GR (0.44 μM), EC reaction mixture (10%)
Chronoamperometry	-0.6	MV (1 mM), GSSG, GR-C8-Au	NADPH (1 mM), GR (0.44 μM), EC reaction mixture (10%)	MV (1 mM) GSSG (10 mM) C8-Au	NADPH (1 mM), GR (0.44 μM), EC reaction mixture (10%)
Chronoamperometry	0.1	MV (1 mM), GSSG (10 mM), GR-C8-Au	NADPH (1 mM), GR (0.44 μM), EC reaction mixture (10%)	MV (1 mM) GSSG (10 mM) C8-Au	NADPH (1 mM), GR (0.44 μM), EC reaction mixture (10%)

Table 1: Experimental conditions of each component in the electrocatalytic reduction of GSSG (EC reaction) by cyclic voltammetry and chronoamperometry, and the biochemical reduction of GSSG (biochemical reaction).

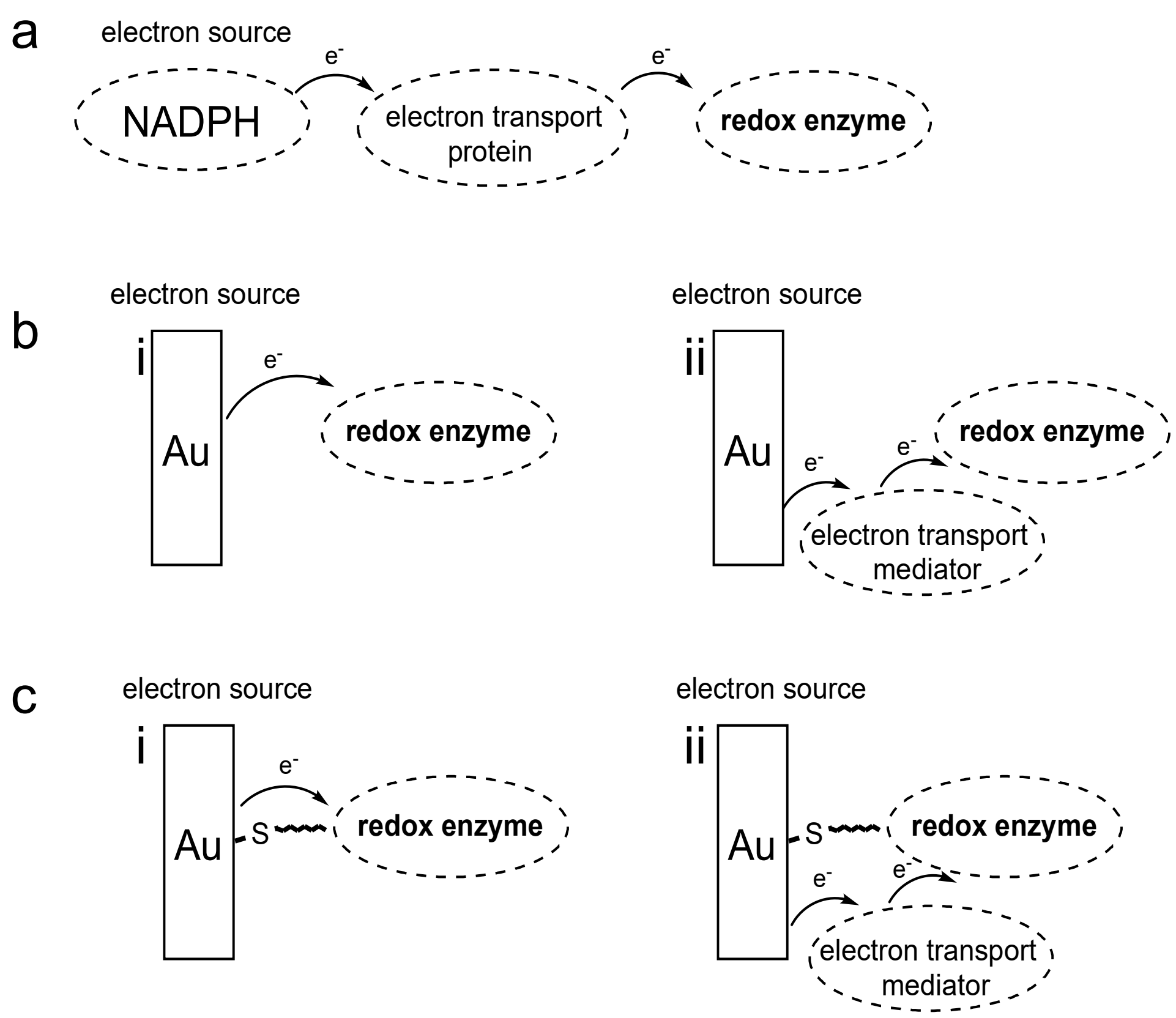

Supplementary Figure S9: Illustration of the different interfaces for assessments of activity. a) $P450_{cam}$ biochemical assay using electron transport proteins, FdX and FdR, where NADPH is the electron source, b) solution-phase electrocatalysis where Au is the electron source and requires the diffusion of i) $P450_{cam}$ without an electron mediator and ii) GR with an electron mediator to the Au electrode surface, and c) surface-adsorbed electrocatalysis where Au is the electron source i) for $P450_{cam}$ without an electron mediator and ii) GR with an electron mediator.

1.5.1 ELECTROCATALYSIS OF SOLUTION-PHASE GR AND P450 BY CYCLIC VOLTAMMETRY

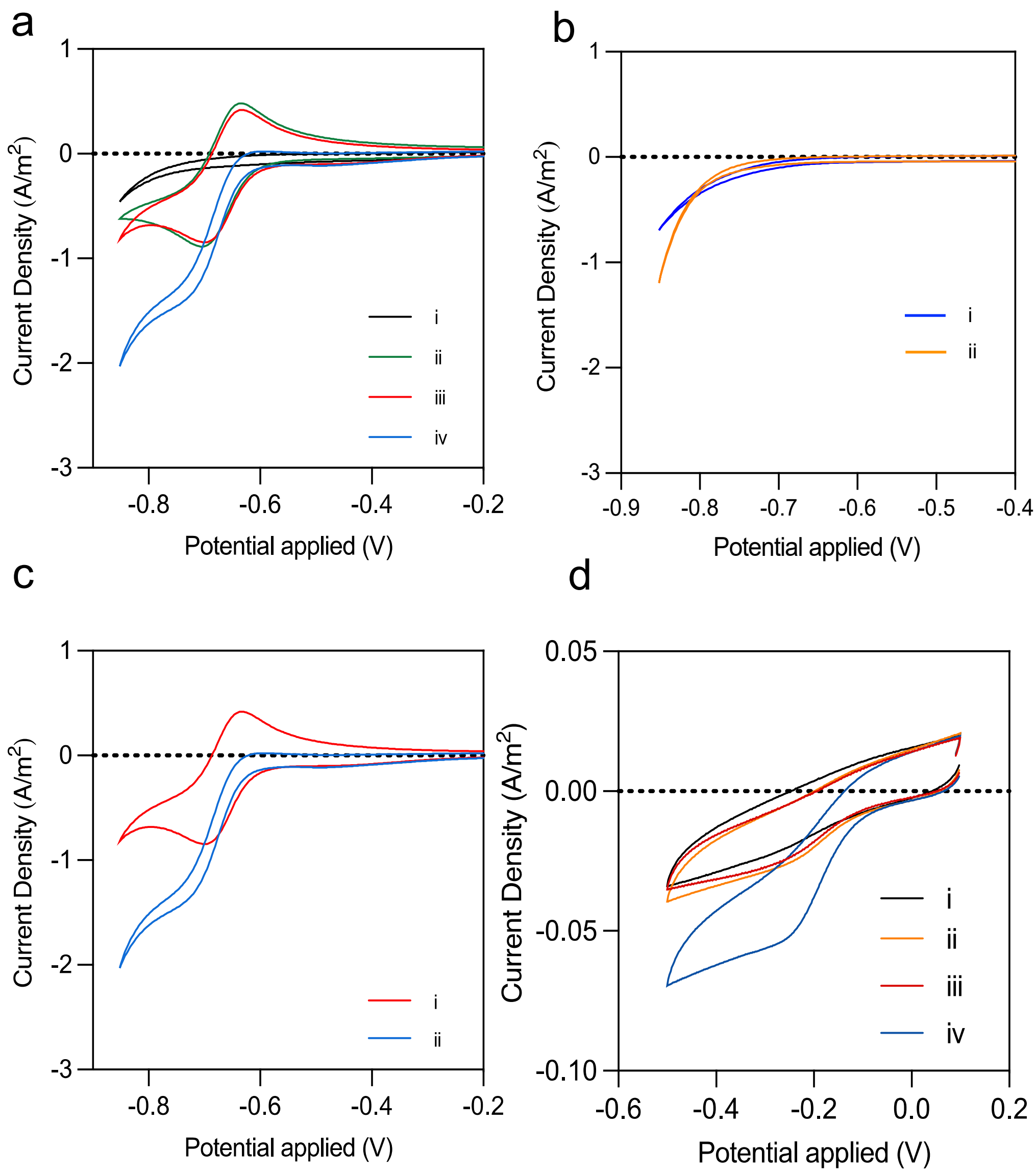

Supplementary Figure S10: Cyclic voltammograms for the solution phase electrocatalysis of GR and P450cam. All GR experiments (a-c) used Tris-KCl (0.2 M, pH 7) as the supporting electrolyte**.** a) Cyclic voltammograms of i) Tris-KCl buffer only, ii) MV (1 mM), iii) MV (1 mM) and GSSG (10 mM) and iv) MV (1 mM), GSSG (10 mM) and GR (4.4 μM). b) Electrochemical study of GSSG (10 mM) using GR as a variable in the absence of MV where i) does not have GR and ii) has GR (4.4 μM). This study shows how GR has no catalytic effect on GSSG, unless MV is present. c) Electrochemical study of GR (4.4 μM) and MV (1 mM) using GSSG (10 mM) as a variable where i) does not have GSSG ii) has GSSG (10 mM). This study shows how GR has no catalytic effects with MV, unless in the presence of GSSG. Cyclic voltammograms were obtained using Au as the working electrode, Pt as the counter electrode and Ag/AgCl (3 M KCl) as reference electrode, scan rate = 0.01 Vs^{-1}. [16] d) Electrochemical voltammograms using phosphate buffer (0.1 M, pH 7) as the supporting electrolyte for i) phosphate buffer only, ii) camphor only (1 mM) iii) $P450_{cam}$ only (20 μM) and iv) $P450_{cam}$ (20 μM) and camphor (1 mM) using Au working electrode, Pt counter electrode *vs* Ag/AgCl 3 M KCl reference electrode. Scan rate 0.005 Vs^{-1}. [17]

In addition to the comparison of the average single-protein frequency data with reported bulk k_{cat} values (Supplementary Section 5 below), we have also extracted a turnover frequency from the electrocatalytic current generated in the cyclic voltametric signal at -0.4 and -0.7 V for P450 and GR, respectively (Fig. S10d-c). ***P450***: Considering the electrode area of 1.6 mm, this yields a net electrocatalytic current of ~60 nA, which corresponds to a total enzymatic activity of $1.2{\cdot}10^{13}$ cycles/min. Such electrocatalytic activity is supplied by only a very small % of enzymes out of all enzymes present in the 20 uM solution (Fig. S10d) that establish an electrical connection to the electrode surface. To evaluate the latter, we use the STM images (Fig. 1c) as a map of the amount of enzymes electrically wired to the electrode surface when exposed to the same 20 uM solution of enzymes. Assuming an homogeneous distribution throughout the macroscopic 1.6 mm electrode surface, this yields a total of $1.5{\cdot}10^{9}$ enzymes wired to the electrode surface. Assigning the above enzymatic activity to the estimated number of electrically connected surface enzymes yields a frequency per enzyme around 7500 min^{-1} (Table 1[c]), in line with the obtained single-enzyme frequencies in Table 1[b]. Note that both electrocatalytic currents and Single-Enzyme Freq (Table 1[b,c]) report only on the active periods of enzyme's activity and omit the quiescent (silent) periods, resulting in a good agreement between the two frequency values. ***GR***: Following the same analysis above for GR, it yields electrocatalytic currents of 1800 nA, which results in frequencies per enzyme around 86000 min^{-1} (Table 1[c]). This value is also in agreement with the Single-Enzyme frequency (new Table 1b) within a factor of two difference. We believe that the latter discrepancy is in line with the fact that larger overpotentials (-0.7 V) have been used to determine the electrocatalytic turnover, which is needed to extract a net electrocatalytic current from the voltametric signal.

1.5.2. UV-VIS ABSORPTION SPECTROSCOPY TO MONITOR NADPH CONSUMPTION BY GR AFTER SOLUTION-PHASE ELECTROCATALYSIS

1.5.2.1 CYCLIC VOLTAMMETRY

For solution-phase electrocatalysis by cyclic voltammetry (**Fig. S10a (iv)**), the electrolyte contained glutathione reductase (4.4 μM), methyl viologen dichloride (1 mM) and GSSG (10 mM) in Tris-KCl (0.1 M, pH 7). The potential voltage applied to the cell was swept from 0 V to -0.85 V at 0.01 V s^{-1} for 20 cycles at room temperature (~22°C). This was repeated in the absence of GR **Fig. S10a (iii)**) as the control experiment.

1.5.2.2 UV-VIS SPECTROSCOPY

NADPH (1 mM) and the electrocatalytic reaction solution (diluted to 10% of its original concentration) in Tris buffer (0.1 M, pH 8) were incubated at room temperature ~22°C for 5 minutes. After 5 minutes, the UV-visible spectrum of the solution was measured between 220–600 nm (**Fig. S11a,** CV).

For the control experiment, NADPH (1 mM), the control electrocatalytic reaction solution (diluted to 10% of its original concentration) in Tris buffer (0.1 M, pH 8) and GR (0.44 μM) were incubated at room temperature ~22°C for 5 minutes. After 5 minutes, the UV-visible spectrum of the solution was measured between 220 – 600 nm (**Fig. S11a,** Control).

The absorbance of NADPH is observed at 340 nm, and the difference between the experimental and control is the consumption of GSSG during the electrocatalysis (**Fig. S11**), and therefore the concentration of GSH produced. The concentration of GSH was calculated in a 1:2 ratio using Beer Lambert's law and the molar extinction coefficient of NADPH of 6220 $M^{-1}\,cm^{-1}$ [6].

1.5.3. UV-VIS ABSORPTION SPECTROSCOPY TO MONITOR NADPH CONSUMPTION BY GR AFTER SURFACE-ADSORBED ELECTROCATALYSIS

1.5.3.1 CHRONOAMPEROMETRY

The electrolyte contained methyl viologen dichlorate (157 μM) and GSSG (1527 μM) in Tris-KCl buffer (0.1 M, pH 7) with GR-C8-Au as the working electrode. The electrochemical potential of the cell was ramped from 0 V to -0.75 V at 10 V s^{-1} and then held at -0.6 V (**Fig. S11b,** -600mV) and 0 V (**Fig. S11c,** 0 mV) for 3 hours at room temperature (~22°C). Control experiments were carried out using open circuit potential (**S11b-c,** Control).

1.5.3.2 UV-VIS SPECTROSCOPY

NADPH (1 mM) and the surface-adsorbed electrocatalytic reaction held at -0.6 V (diluted to 10% of its original concentration) and additional GR (0.44 μM) in Tris buffer (0.1 M, pH 8) were incubated at room temperature ~22°C for 5 minutes. After 5 minutes, the UV-visible spectrum of the solution was measured between 220–600 nm (**Fig. S11b,** -600mV). This was repeated for the surface-adsorbed electrocatalytic reaction held at 0 V (**Fig. S11c**, 0mV).

For the control experiment, NADPH (1 mM), the control electrocatalytic reaction (containing 1mM GSSG) in 0.1 M Tris (pH 8) and GR (0.44 μM) were incubated at room temperature ~22°C for 5 minutes. After 5 minutes, the UV-visible spectrum of the solution was measured between 220 – 600 nm (**Fig. S11a-c**, Control).

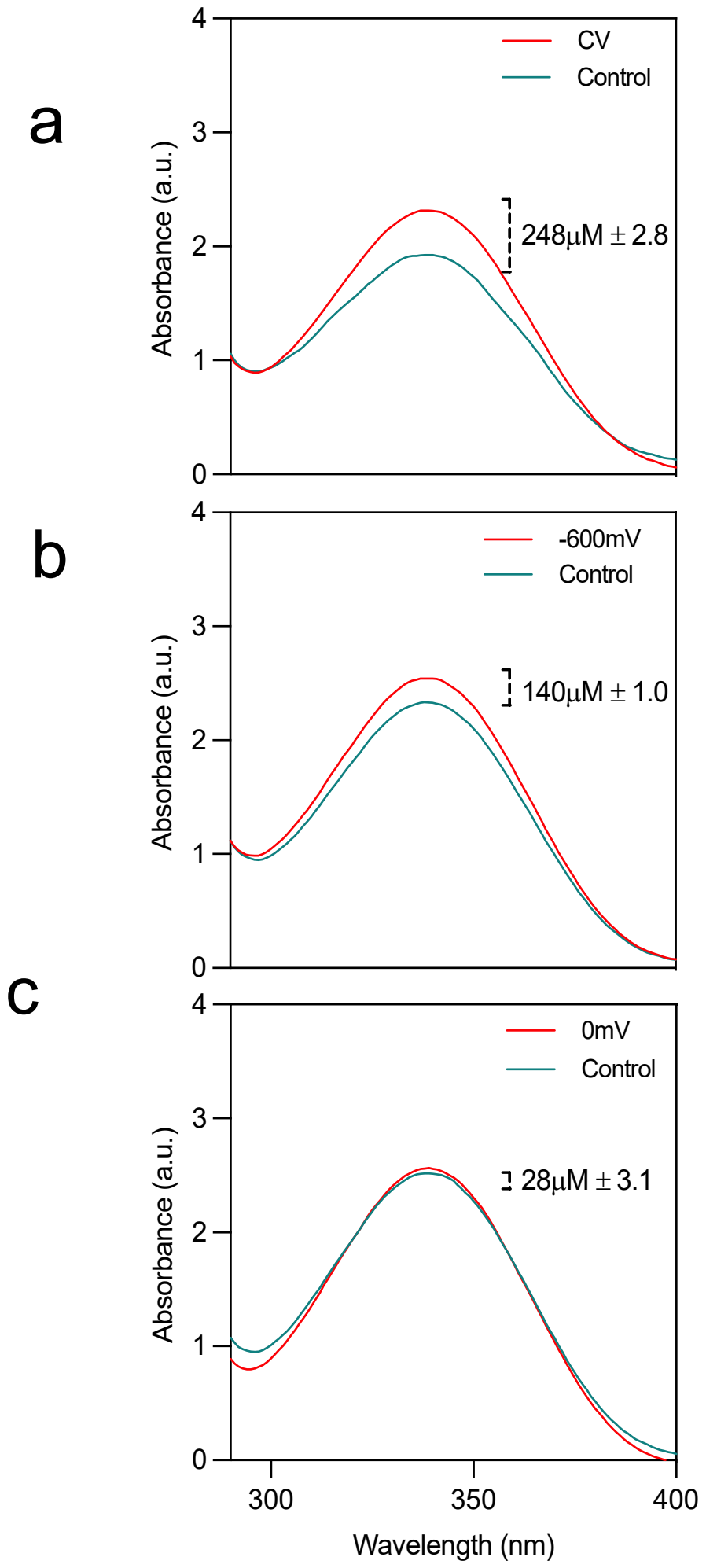

Supplementary Figure S11: Spectrophotometric NADPH Consumption Assay. NADPH consumption assay monitored using UV-vis for the reduction of remaining oxidised glutathione (GSSG) after a) cyclic voltammetry between 0 V to -0.85 V with 1 mM NADPH in the presence (CV) and absence (control) of GR in solution. Chronoamperometry using immobilised GR-C8-Au(111) held at b) – 0.6 V for 3 hours (-600mV) and open circuit potential (control) and c) 0 V for 3 hours (0mV) and open circuit potential (control), yielding GSH concentrations of 248 μM, 140 μM and 28 μM respectively.

1.6. CHRONOAMPEROMETRIC ELECTROCATALYSIS OF SOLUTION PHASE $P450_{CAM}$ AND SURFACE-ADSORBED P450CAM-C8-AU

Measurements were carried out using NOVA software (Metrohm Autolab, the Netherlands) in a three-electrode configuration: 10 mm Au(111) working electrode, a Pt counter electrode and a Ag/AgCl (3 M NaCl) reference electrode. The electrochemical cell content (200 μL) was extracted after the chronoamperometric electrocatalysis using ethyl acetate (2 x 100 μL) and analysed by Gas Chromatography Mass Spectrometry (Shimadzu, GCMS -QP2020 NX).

Interface	Components	Cell volume (μL)	Reaction time (h)	Cell potential (V)	Temperature (°C)
Solution-phase	$P450_{cam}$ (20 μM),<br>D-camphor (final conc. 10 mM),<br>phosphate buffer (0.1 M, pH 7)	200	2.5	0	22°C
Solution-phase	$P450_{cam}$ (20 μM),<br>D-camphor (final conc. 10 mM),<br>phosphate buffer (0.1 M, pH 7)	200	2.5	-0.75	22°C
P450-C8-Au	D-camphor (final conc. 1 mM),<br>phosphate buffer (0.1 M, pH 7)	200	7	0	22°C
P450-C8-Au	D-camphor (final conc. 1 mM),<br>phosphate buffer (0.1 M, pH 7)	200	7	-0.5	22°C

For solution-phase, the electrolyte contained $P450_{cam}$ (20 μM) and D-camphor (final conc. 10 mM) in phosphate buffer (0.1 M, pH 7) with a total volume of 200 μL. The electrochemical potential of the cell was ramped from 0 V to -0.75 V at 0.01 Vs^{-1} and then held at -0.75 V for 3 hours at room temperature ~22°C. Control experiments were carried using 0 V, and in the absence of $P450_{cam}$. For P450-C8-Au, the electrolyte contained D-camphor (1 mM) in 0.1 M phosphate (pH 7) with a total volume of 200 μL. The electrochemical potential of the cell was ramped from 0 V to -0.5 V at 0.01 Vs^{-1} and then held at -0.5 V for 7 hours at room temperature ~22°C.

The main m/z ion peaks for 5-exo-hydroxycamphor are 111, 125, 153, 168 (**Fig. S12**). The mass spectrum of a-e is shown for the retention time of 5-exo-hydroxycamphor, and only c and f indicate formation of product.

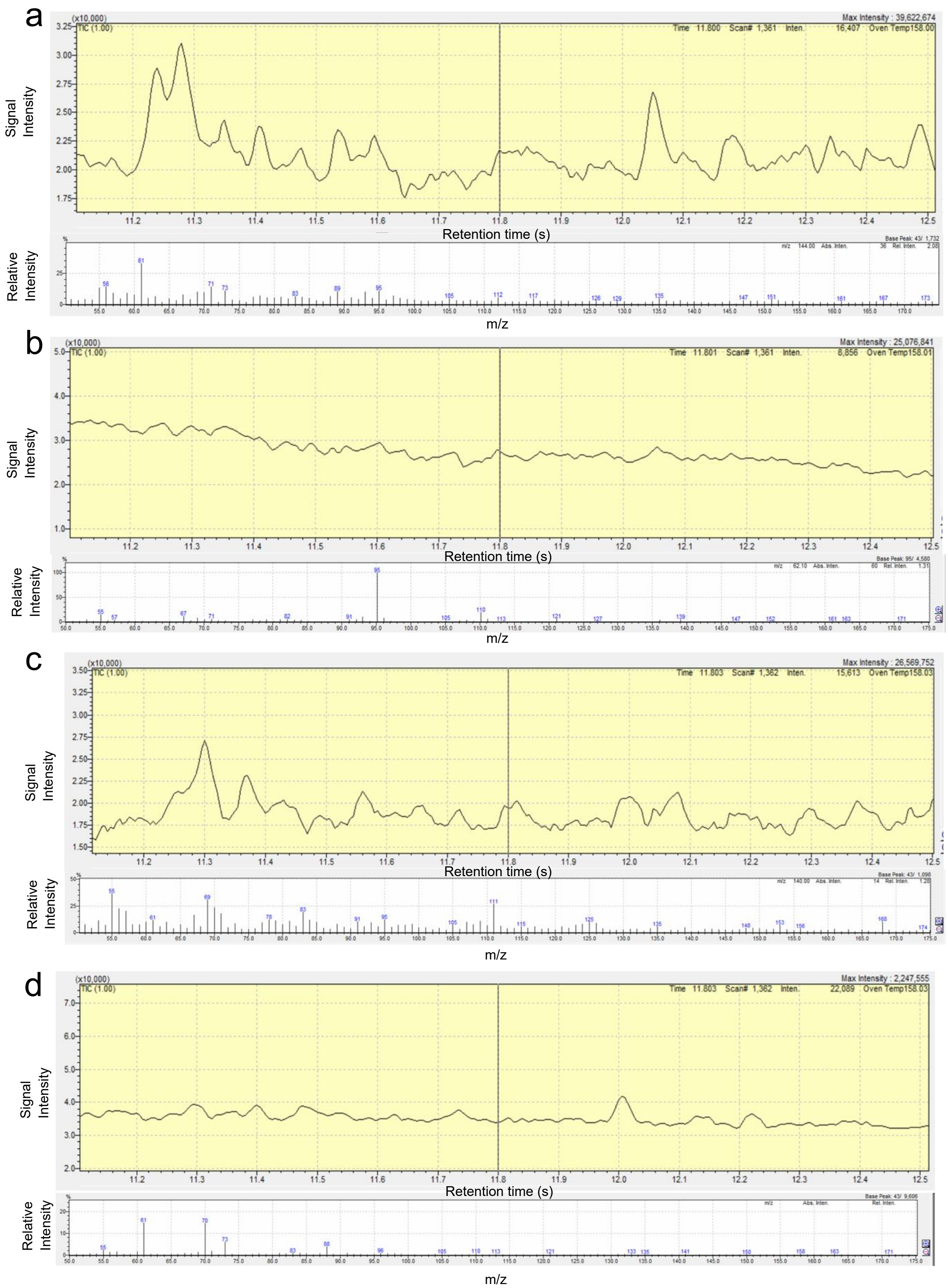

a
Max Intensity : 39,622,674
Time 11.800 Scan# 1,361 Inten. 16,407 Oven Temp158.00
Signal Intensity
Retention time (s)
Relative Intensity
m/z
b
Max Intensity : 25,076,841
Time 11.801 Scan# 1,361 Inten. 8,856 Oven Temp158.01
Signal Intensity
Retention time (s)
Relative Intensity
m/z
c
Max Intensity : 26,569,752
Time 11.803 Scan# 1,362 Inten. 15,613 Oven Temp158.03
Signal Intensity
Retention time (s)
Relative Intensity
m/z
d
Max Intensity : 2,247,555
Time 11.803 Scan# 1,362 Inten. 22,089 Oven Temp158.03
Signal Intensity
Retention time (s)
Relative Intensity
m/z

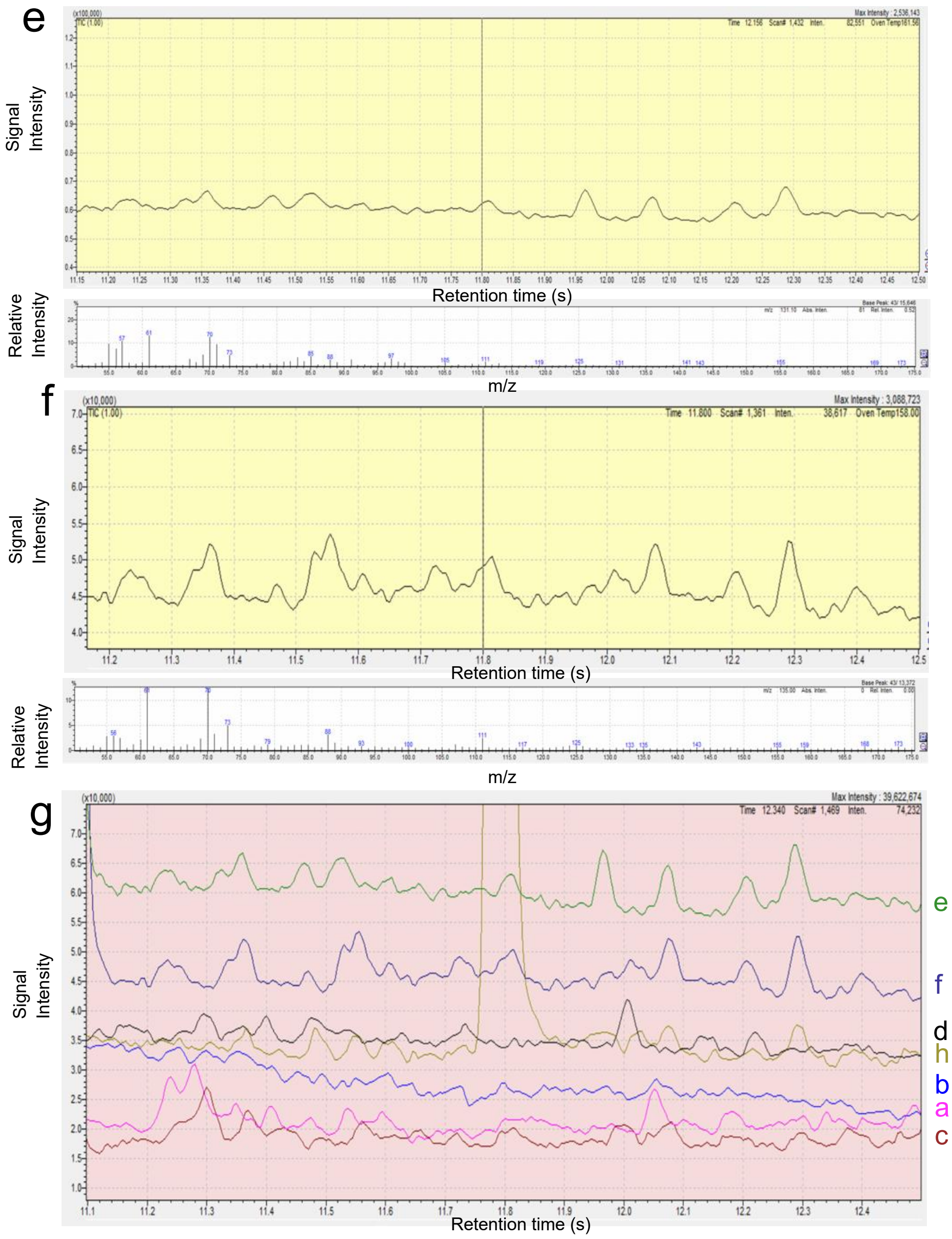

Supplementary Figure S12: Chronoamperometric electrocatalysis of $P450_{cam}$. GCMS analysis of solution-phase and surface-adsorbed chronoamperometry electrocatalysis. Each condition has its chromatogram and its corresponding mass spectrum in the elution region of 5-exo-hydroxycamphor (~11.8 s; m/z 168) (Fig S5). Solution-phase electrocatalysis of $P450_{cam}$ and D-camphor was carried out at a) -0.75 V in the absence of $P450_{cam}$ b) 0 V in the presence of $P450_{cam}$ and c) -0.75 V in the presence of $P450_{cam}$, chromatogram and MS spectrum indicate presence of 5-exo-hydroxycamphor product. Surface-adsorbed electrocatalysis of P450-C8-Au and D-camphor was carried out at d) -0.5 V in the absence of $P450_{cam}$ (C8-Au), e) 0 V and f) -0.5 V both in the presence of $P450_{cam}$. chromatogram f) and MS spectrum indicate formation of product 5-exo-hydroxycamphor. g) Overlay of all the conditions with *h* trace being the biochemical assay ($P450_{cam}$ (20 μM) incubated with camphor (50 μM), ferredoxin (FdX, 0.08 gL^{-1}), ferredoxin reductase (FdR, 0.08 U) and NADPH (1 mM) in phosphate buffer (0.1 M, pH 7) for 2.5 hrs at 25°C).

1.7. Calibration of nanoscale gap distance for the P450-C8-Au and GR-C8-Au interfaces

For both interfaces, the apiezon wax coated Au tip was allowed to approach the surfaces until a current setpoint of 250 pA was reached. After stabilisation of the tip-to-sample distance, the feedback loop was turned off and decay curves were collected for each interface. The curves were overlayed to calculate the average beta decay constant (Fig. S13).

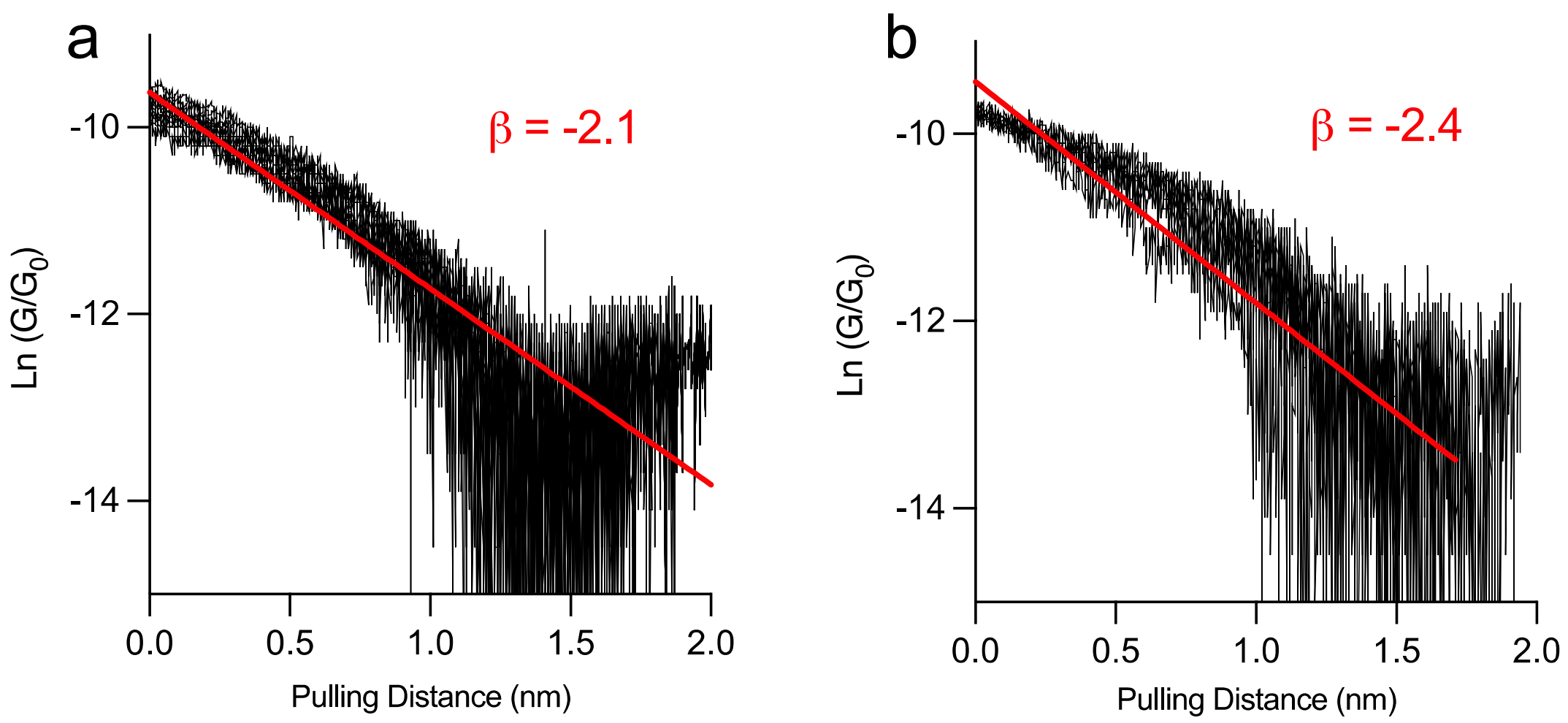

Supplementary Figure S13: Calibration of nanoscale gap distance in 0.1 M PB (pH 7) and 0.1 M Tris-KCl (pH 7), respectively. Average beta decays of 2.1 and 2.4, and a tip distance of 5.7 nm and 5.2 nm were obtained for a current setpoint of 40 pA for a) GR-C8-Au and b) P450-C8-Au, respectively.

1.8. AFM Imaging

Imaging of substrates were conducted on the Dimension Icon Atomic Force Microscope, operating on the Bruker Nanoscope 9.7 interface. AFM imaging was carried out in tapping mode in air using Tap300Ai-G silicon AFM probes (resonant frequency of 300kHz).

1.9. Height and diameter analysis of STM/AFM images

The mean heights were obtained from AFM images (**Fig. S15**) and the mean diameters were obtained from EC-STM images (**Fig. S16**) using Gwyddion software and compared to the crystallographic sizes (**Fig. S14**).

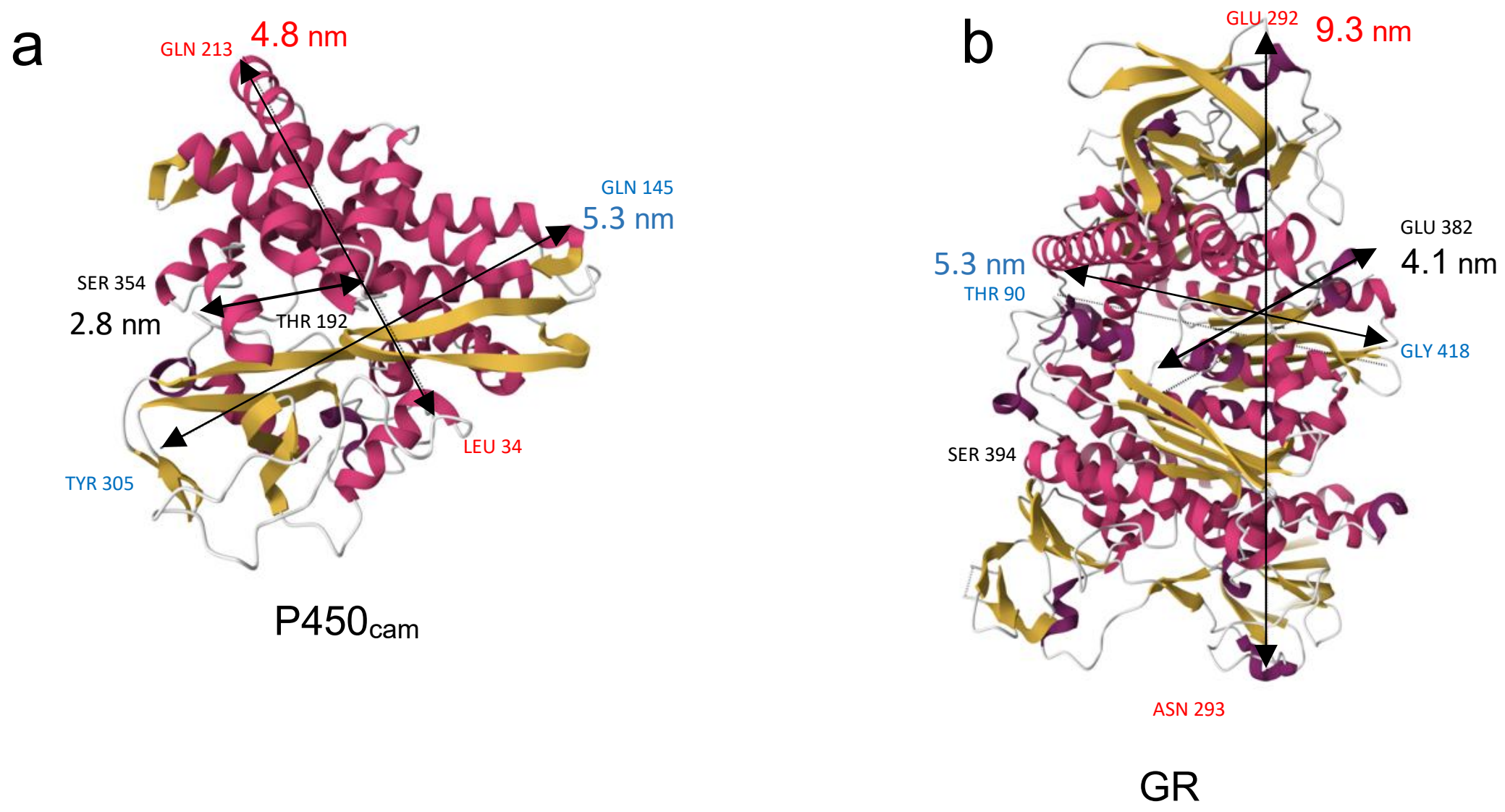

Supplementary Figure S14: Enzyme dimensions based on X-ray crystal structures. Residues that were selected to measure dimensions of the enzymes are indicated. a) $P450_{cam}$, width = 4.8 nm (between Gln213 and Leu34) length = 5.3 nm (between Tyr305 and Gln145), depth = 2.8 nm (between Ser354 and Thr192) and b) GR (monomer a and b), width = 5.3 nm (between Thr90(b) and Gly418(b)), length = 9.3 nm (between Glu292(b) and Asn293(a)), depth = 4.1 nm (between Ser394(a) and Glu283(b). $P450_{cam}$ (PDB: 2CPP) and GR (PDB: 2HQM) were both visualised and their dimensions measured on the RCSB interface.

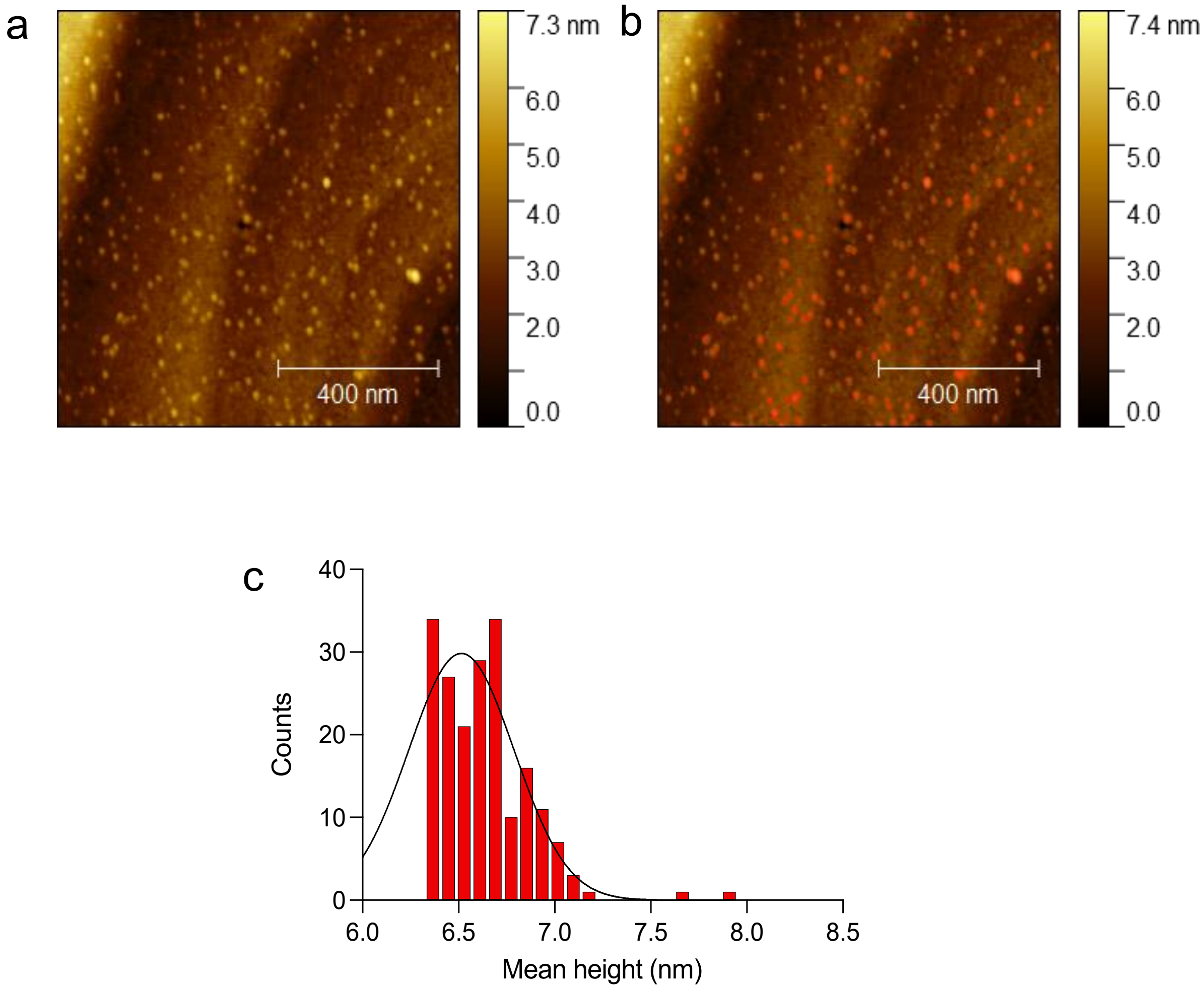

Supplementary Figure S15: Mean Height Analysis of GR-C8-Au using AFM Imaging. a) AFM images of GR immobilised on 1-octanethiol-Au with scan size 1μm. b) Red areas are masked enzyme particles that were considered to measure the mean height. c) Distribution of mean height obtaining an average of 6.5 nm.

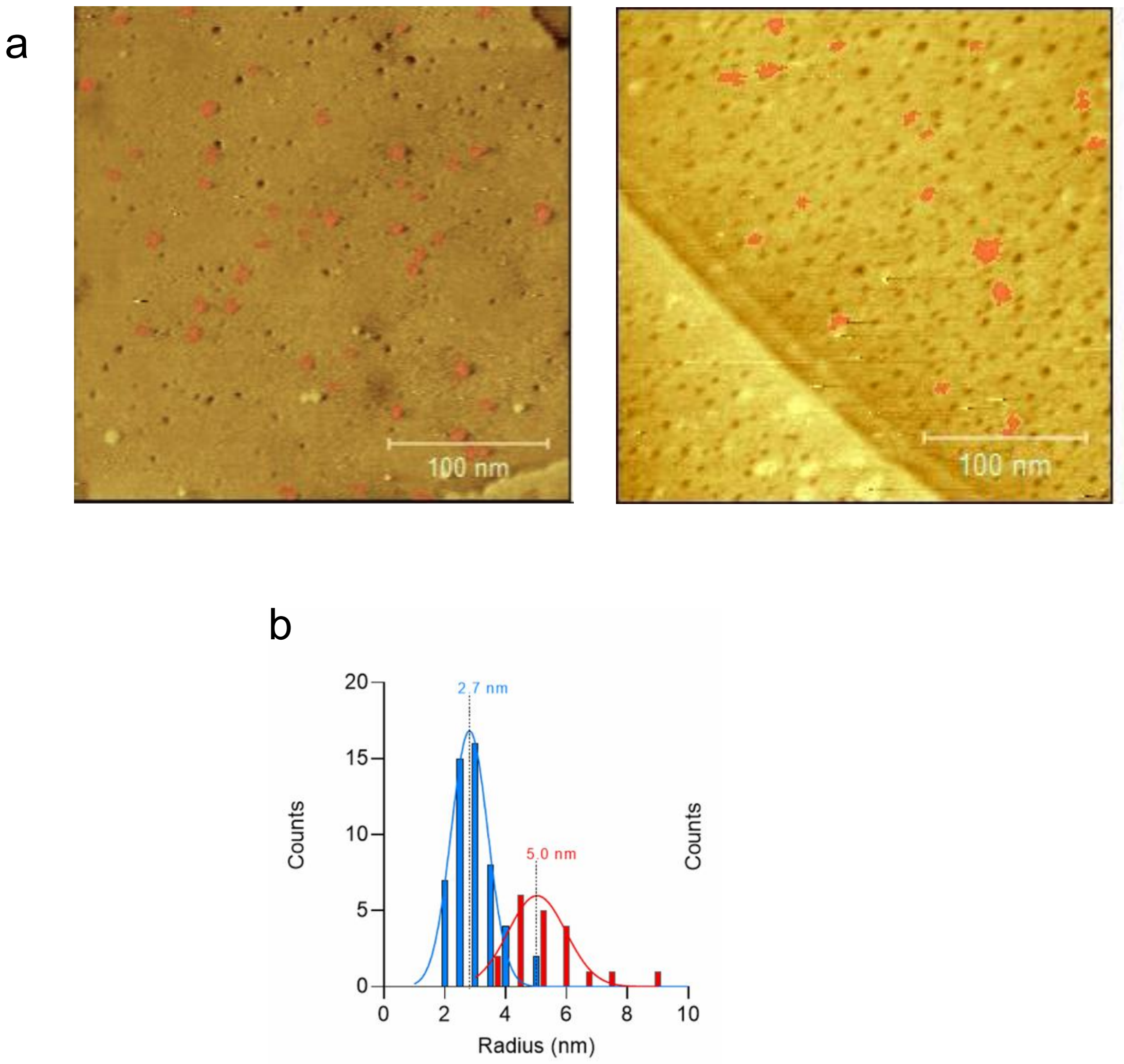

Supplementary Figure S16: Mean Diameter Analysis using EC-STM Imaging. a) EC-STM images held at an electrochemical potential of 0V, bias voltage +0.1 V and current setpoint 0.05 nA with Au(111) as the working electrode, Pt as the platinum electrode and Ag/AgCl (3M KCl) as the reference electrode. Red areas are masked enzymes particles on surface that were considered for the average height and diameter of $P450_{cam}$ (left) and GR (right). b) Mean distribution of radius obtaining 2.7 nm and 5.0 nm for $P450_{cam}$ and GR, respectively.

1.10. AFM SCRATCHING OF 1-OCTANETHIOL-AU

Scratching of substrates were conducted on the Dimension Icon Atomic Force Microscope (AFM), operating on the Bruker Nanoscope 9.7 interface in TM deflection mode in air. AFM images were analysed using Gwyddion software (**Fig. S17**).

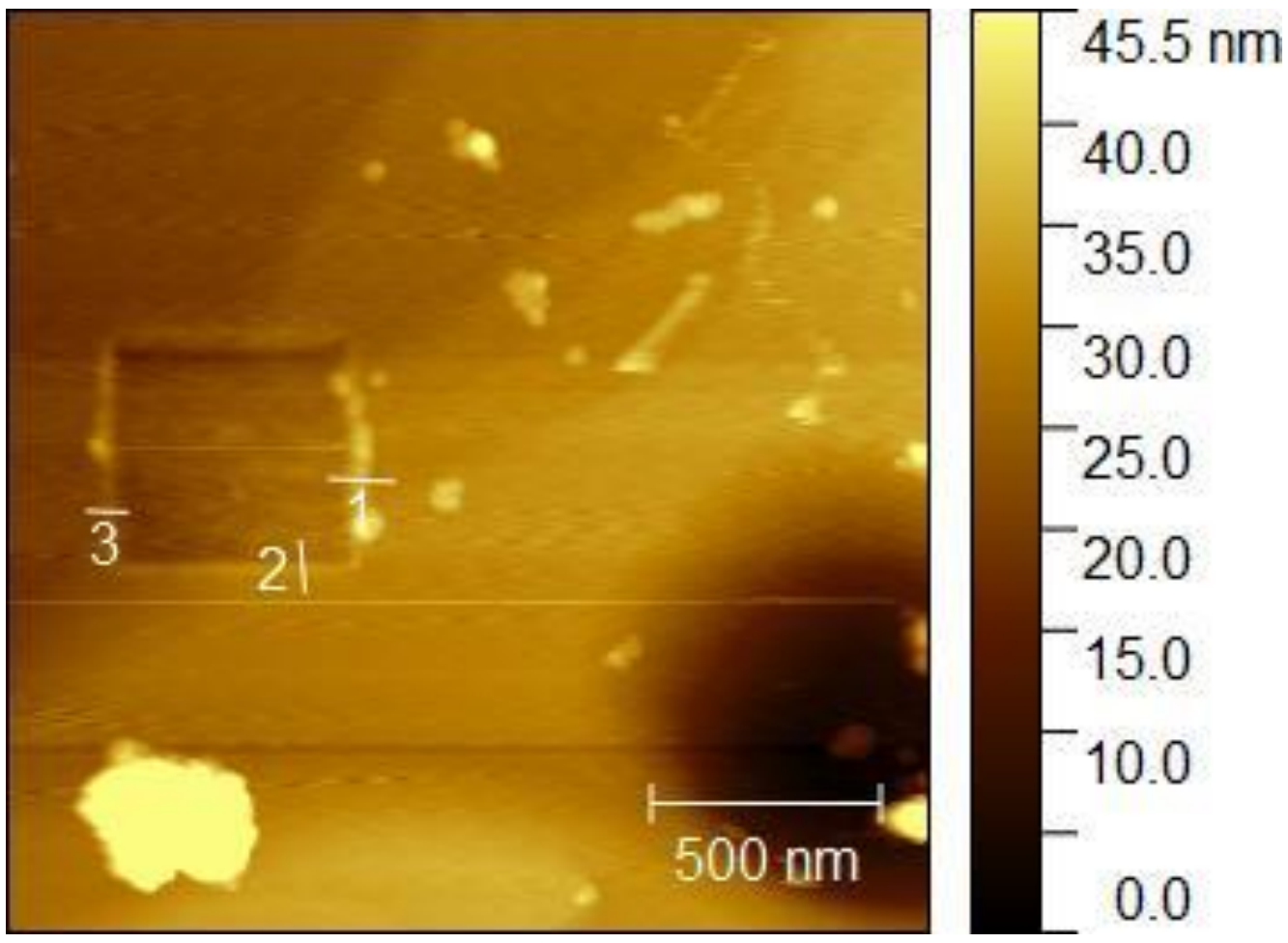

n	Δx [µm]	Δy [µm]	φ [deg]	R [µm]	Δz [nm]
1	0.140	0.006	-2.6	0.140	1.14
2	0.010	0.108	-85.0	0.109	0.84
3	-0.086	-0.003	177.9	0.086	0.91

Supplementary Figure S17: AFM Scratching of 1-octanethiol-Au. The average height of 1-octanethiol was ~ 1 nm.

1.11. Electrochemical desorption of 1-octanethiol

Measurements were carried out using NOVA software (Metrohm Autolab, the Netherlands) in a three-electrode configuration (**Fig. S18**): 10 mm Au(111) working electrode, a Pt counter electrode and a Ag/AgCl (3 M NaCl) reference electrode. The desorption of 1-octanethiol-Au was carried out in 0.05 M NaOH and the potential was swept from 0 V to -1.3 V at 0.1 Vs^{-1} after chronoamperometry (-0.6 V for 3 hours).

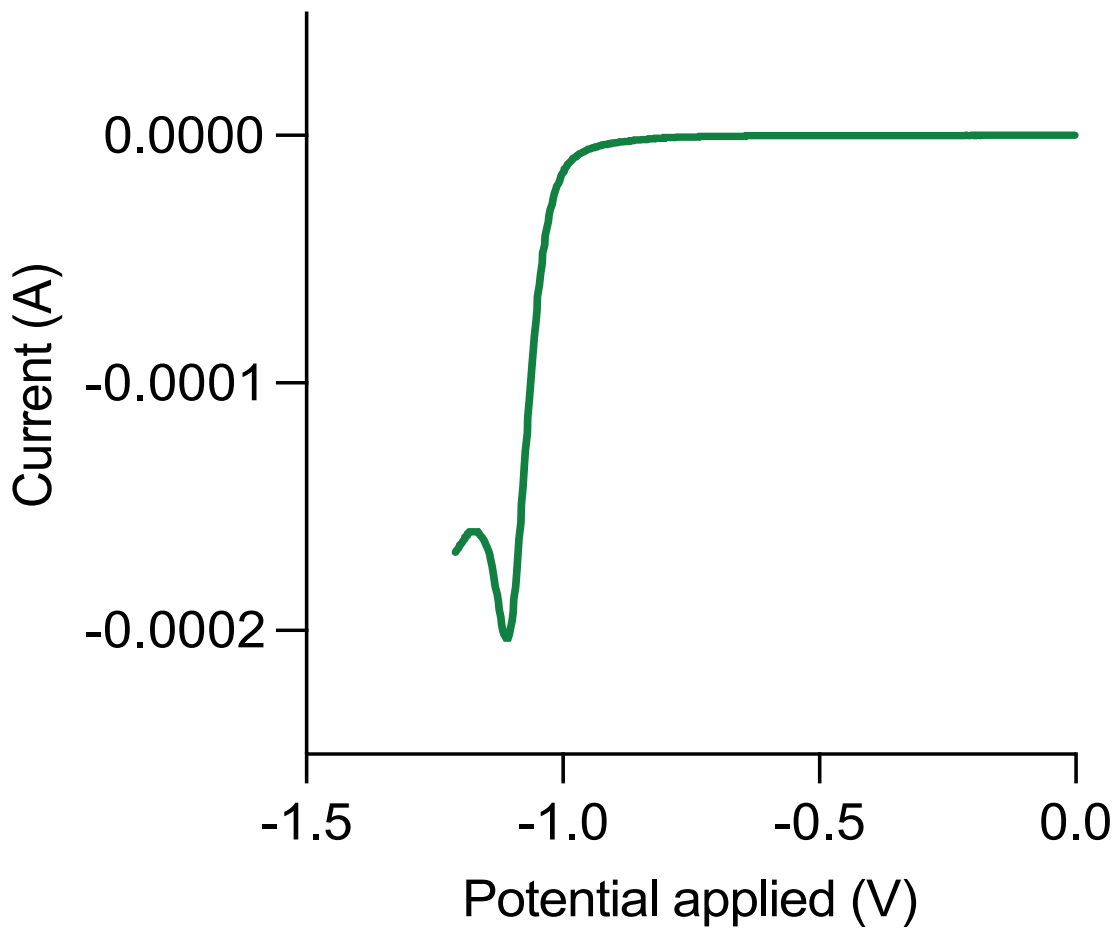

Supplementary Figure S18: Electrochemical Desorption of 1-octanethiol-Au (C8-Au). Desorption was obtained using Au as the working electrode, Pt as the counter electrode and a Ag/AgCl (3 M KCl) in 50 mM NaOH. Scan rate 0.1 Vs^{-1}.

2. Single enzyme conductance analysis

2.1 1D conductance histograms of surface-adsorbed P450$_{CAM}$ and GR of the silent traces under different catalytic conditions

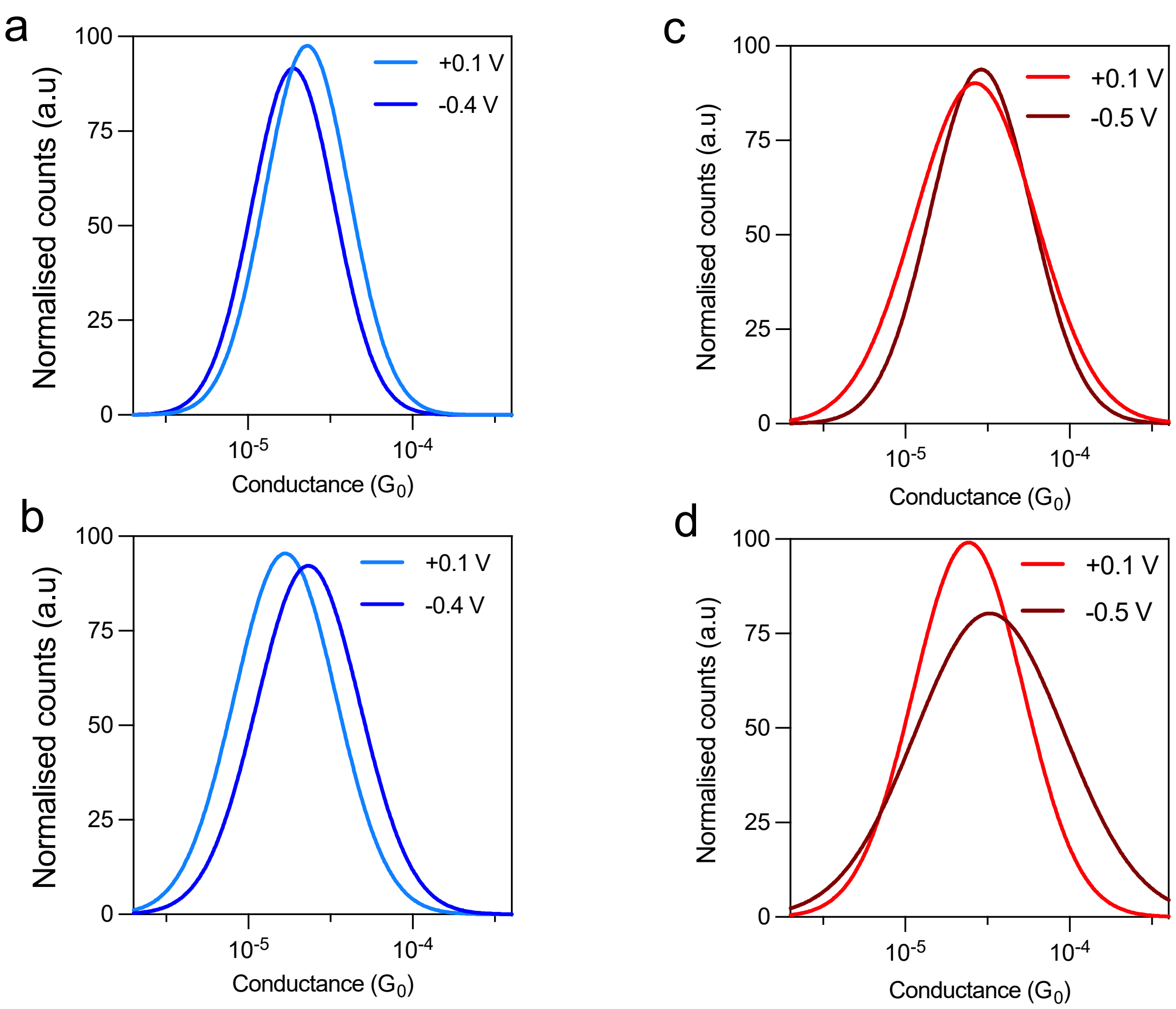

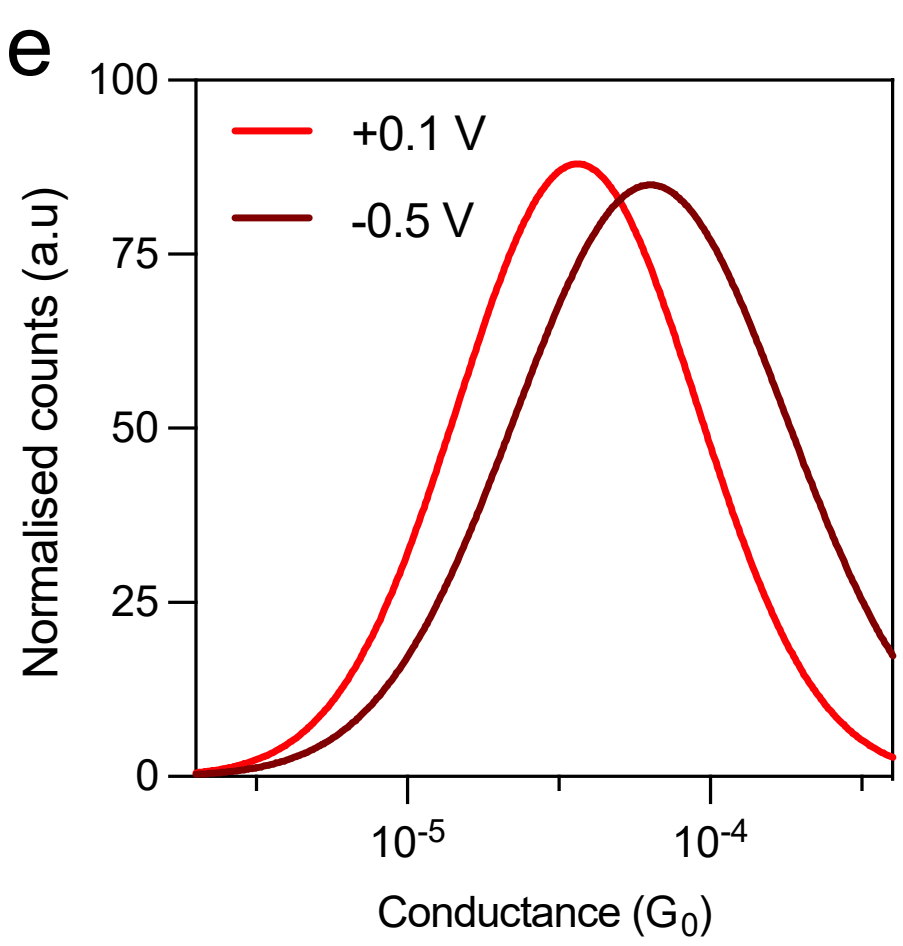

f

	+0.1 V	-0.4 V
**P450**	2.2×10^{-5}	1.8×10^{-5}
**P450 + camphor**	1.7×10^{-5}	2.4×10^{-5}
	**+0.1 V**	**-0.5 V**
**GR**	2.6×10^{-5}	2.9×10^{-5}
**GR + MV**	2.4×10^{-5}	3.3×10^{-5}
**GR + MV + GSSG**	3.6×10^{-5}	6.5×10^{-5}

Supplementary Figure S19: 1D Histograms of single-protein conductance traces for P450-C8-Au in a) absence of camphor and b) presence of camphor, and for GR-C8-Au in c) absence of MV and GSSG, d) presence of MV and e) presence of MV and GSSG. f) Table of average conductance values of P450-C8-Au and GR-C8-Au under each a-e conditions.

The 1D histograms are a collation of ~3200–4000 *silent traces* for surface-adsorbed P450$_{cam}$ **(Fig. S19 a-b)** and GR **(Fig. S19 c-e)** under two electrochemical conditions. Fig. S19 shows the similarities in the conductance values of the protein in the presence of substrate and/or mediator and thus the binding of the substrate/mediator does not significantly influence the protein conductance.

2.2 STM APPARENT HEIGHTS OF P450$_{CAM}$ UNDER THE DIFFERENT CATALYTIC CONDITIONS

STM images of the surface-adsorbed P450$_{cam}$ were captured within the electrochemical potential range of -0.2 V – 0.45 V. Whilst Fig. 1e shows the average of the enzyme height as a function of the electrochemical potential, we have further in-detail analysed the individual heights of the P450$_{cam}$ molecules to see the distribution. We observe two distinct populations of the observed STM apparent heights with average lower (**Fig. S20,** blue curve) and higher values (**Fig. S20,** red curve) within the two clusters of 0.44 (±0.05) nm and 0.69 (±0.03) nm, respectively for both off- and in-redox resonance scenarios. At -0.2 V, the redox energy state of *heme* is off-resonance resulting in 93% of the proteins to be at the low apparent height thus lowering the average protein height. Comparatively, at -0.35 V when the redox energy state of *heme* is able to come into resonance, 61% of the proteins remain at low apparent height

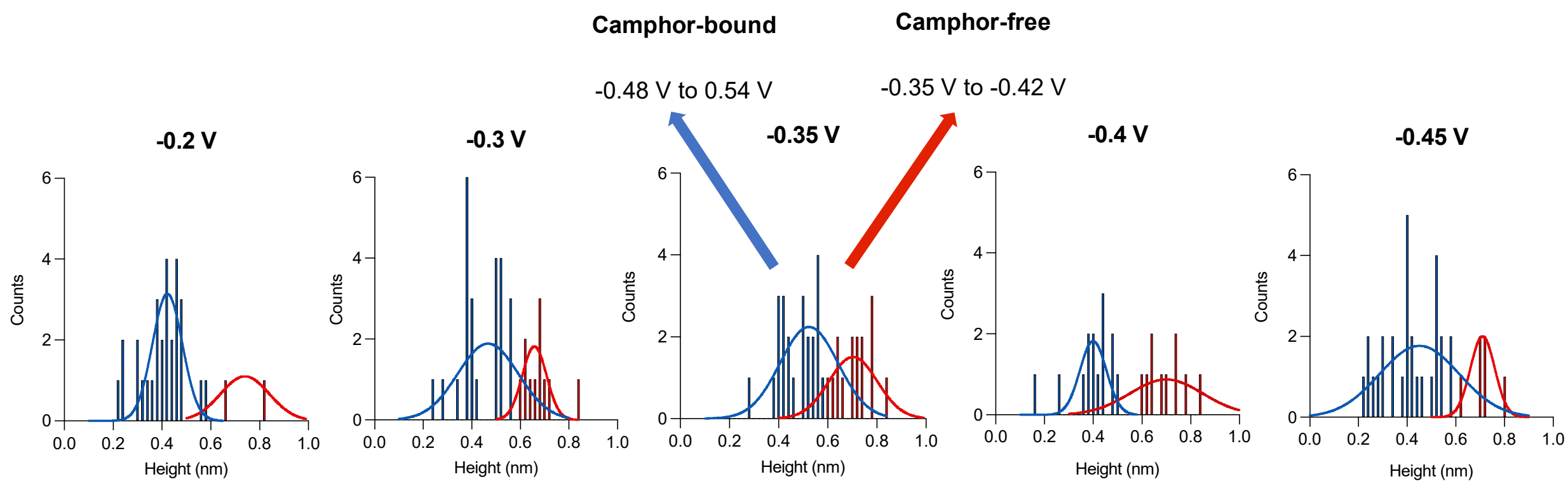

	Percentage of P450 molecules (%)	
Potential (V)	Lower Current-Height Distribution	Higher Current-Height Distribution
-0.20	93	7
-0.30	69	31
-0.35	61	39
-0.40	58	42
-0.45	82	18

Supplementary Figure S20: Percentage of individual P450$_{cam}$ molecules with a low or high STM apparent height distribution. Top: Apparent height bimodal distribution of individual P450$_{cam}$ molecules on the P450$_{cam}$-C8-Au interface at potentials between -0.2 V and -0.45 V, showing an average low apparent height of 0.44 (±0.05) nm for an inactive enzymatic state (red distribution) and a high apparent height of 0.69 (±0.03) nm for active enzymatic state (blue distribution). Bottom: percentage of individual P450$_{cam}$ molecules with a lower and higher current-height distribution. All images were obtained using 0.1 M phosphate buffer, with gold working electrode, Pt counter electrode and Ag/AgCl (3 M KCl). Bias voltage = +0.1 V, current setpoint = 1 nA.

value whilst 39% increases to the higher distribution. At more negative potentials below -0.35 V, the *heme* redox state would otherwise be off-resonance but is momentarily brought into resonance due to the generation of subtle, short-lived oxidation steps by the catalytic events. Thus, this higher distribution remains broad >-0.35 V as a result of the induced enzymatic reaction, though with fewer proteins. At very negative potentials (-0.45 V), the *heme* oxidised state will be no longer captured in-resonance with the corresponding decrease again in STM apparent height.

3. CATEGORISATION AND ANALYSIS OF BLINKING TRACES

3.1. AUTOMATIC DATA SORTING

The obtained blinks or traces were categorised as *silent* blink, where only one level of conductance is observed, or as *switching* blink where two levels of conductance fluctuations can be observed. A total number of ~8000 single traces were analysed using a self-writing algorithm with Python 3.11. The raw

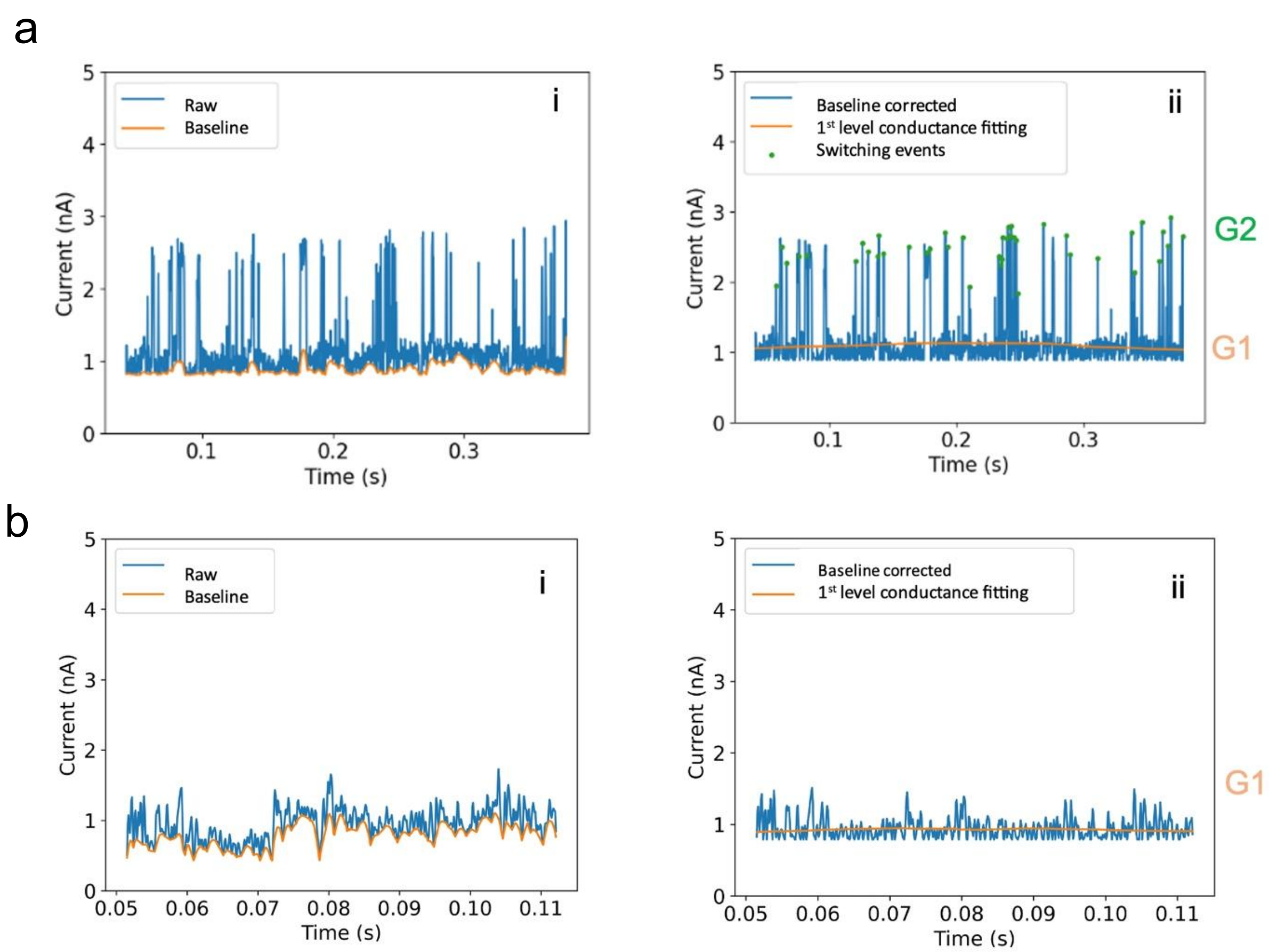

Supplementary Figure S21: Categorisation of blinks using an algorithm on Python 3.11, where (A) represents a switching trace and (B) is a silent trace. i) raw data (blue) with a fitted baseline to correct STM tip drift (orange), ii) baseline corrected data (blue) with the first level of conductance fitted (orange). The green dots indicate the switching events.

data were baseline corrected to align the first level of conductance, as the current through the protein changes markedly over time due to thermal and/or mechanical drifts. As a result, the value of baseline-corrected data is the following: raw data - baseline + mean(baseline). The mean (baseline) is to keep the conductance the same throughout all raw data. The first level of conductance in the baseline-corrected data was fitted with the Savitzky-Golay filter under zero-order polynomial. Single traces that contain switching events were selected by calculating the distance between the fitted first-level conductance G1 (**Fig. S21**, orange line) and the peak maximum of the spikes G2 (**Fig. S21**, green dots). The optimised conductance difference was set as G2 $\geq$ 2.5*G1 for P450 and G2 $\geq$ 2.8*G1 for GR, as determined from the average conductance measurements of the two proteins (**Fig. 4c-d**). The switching events were automatically detected.

3.2. FREQUENCY ANALYSIS

To dig into the origin of the observed two-levels of conductance fluctuation, we first used a machine-learning Gaussian Mixture Model (GMM), to classify the two levels of current.[7] We chose this initial distribution probabilistic model because the observed fluctuation may adhere to Gaussian distributions in the conductance as suggested by the deconvolution of **Figs. 4c-d**. GMMs consider the entire analysed dataset as a mixture of multiple Gaussian distributions. Each of the distributions is associated with a finite "weight" that describes the importance of each component in the mixture.[8] Thus, the probability density function of a GMM is a weighted sum of the probability density functions of each individual Gaussian component. Unlike traditional clustering methods, which assign each data point to a single cluster, the GMM is based on soft or "fuzzy" clustering.[9] Fuzzy clustering allows the probabilistic assignment of data elements to clusters, but instead of a (hard) assignment where a data point belongs exclusively to one cluster, fuzzy clustering assigns probabilities to each element that are linked to its likelihood of belonging to each cluster. In the context of our GMM, the clustering likelihood relies on the probability density function of the model. For a given element, the GMM calculates its probability density of being generated by each Gaussian component in the mixture. These probability densities thus provide fuzzy clustering with the degree of membership of each element for each cluster. The GMM clustering of the monitored current's data points resulted in into two distinct components for both the GR and P450 systems. The populations of the two cluster assignments, unequivocally assigned to the high conductance (HC) and low conductance (LC) current levels, follow a clear Gaussian distribution (see **Fig. S22**).

To further elucidate the underlying sequential nature of the fluctuations, far beyond the empirical classification of the GMM, we used a machine learning-based Hidden Markov Model (HMM).[10] We opted for the application of HMM due to the stochastic nature inherent in bistate phenomenology.[11,12,13] HMM is a suitable method for modelling sequential data in which observables are generated by a process that transitions between hidden states, that are not directly distinguishable by the observer.[14] HMM considers the transition between states to be probabilistic, where the probability of transitioning from one state to another depends exclusively on the current state (Markov property). This is consistent with the stochastic nature of processes that exhibit a dependence of future states on current states, regardless of the sequence of states that preceded them.[15] In this research we employed a HMM-based processing and analysis methodology, as detailed in our previous publication on this topic.[11] This HMM process provided a cluster assignment equivalent to the GMM that identified the LC and HC current levels for each protein (see confusion matrix in **Fig. S23**). This classification equivalence supports the notion that the observed switching behaviour between the two current levels is not random, but governed by a predictable, state-dependent Markovian process. In other words, it confirms the stochastic nature of the switching between the discrete current states for both proteins.

The HMM provides a dynamic understanding of the switching behaviour, as it not only identifies the different conductance levels but also captures the temporal sequence in which these states occur. On this basis, we carried out an exhaustive frequency analysis of the fluctuation events.

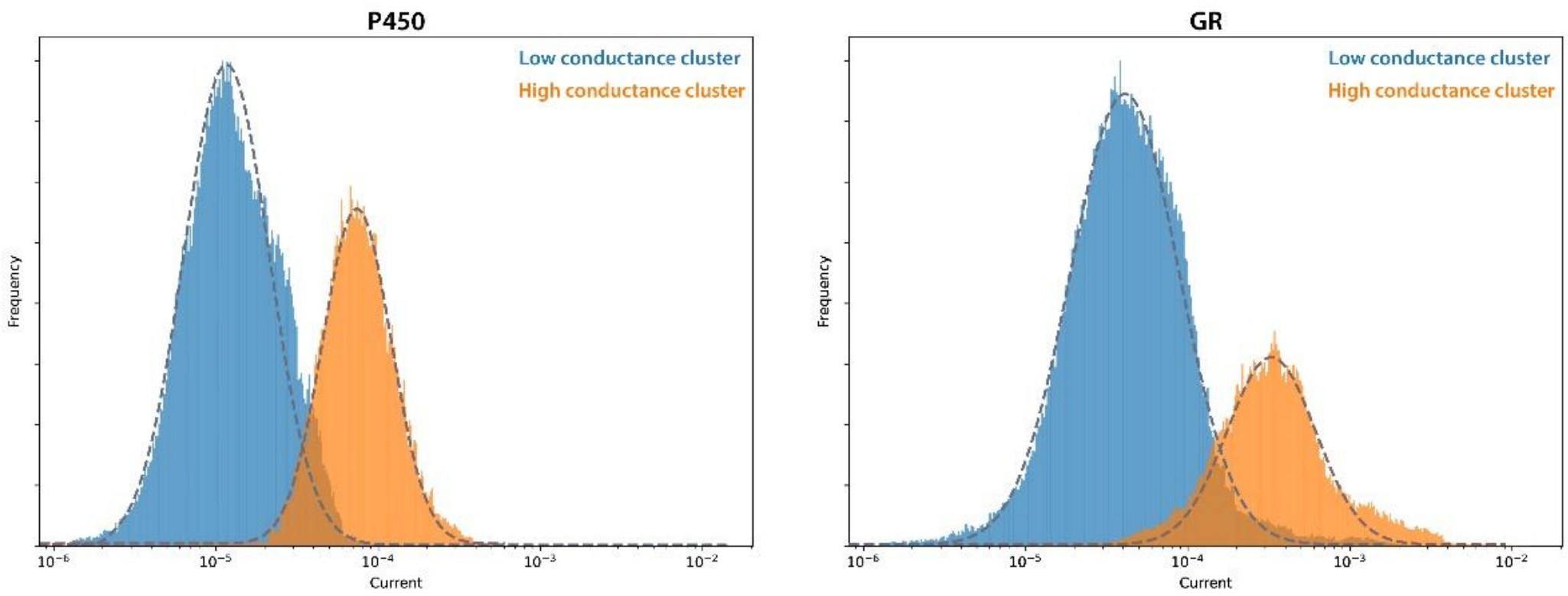

Supplementary Figure S22. Semi-log histograms for both P450 (left) and GR (right) proteins constructed from the populations of the two conductance levels identified by the Gaussian Mixture Model (GMM). Note the clear Gaussian distributions for low (blue) and high (orange) conductance clusters in both cases. Dashed grey traces follow Gaussian fittings.

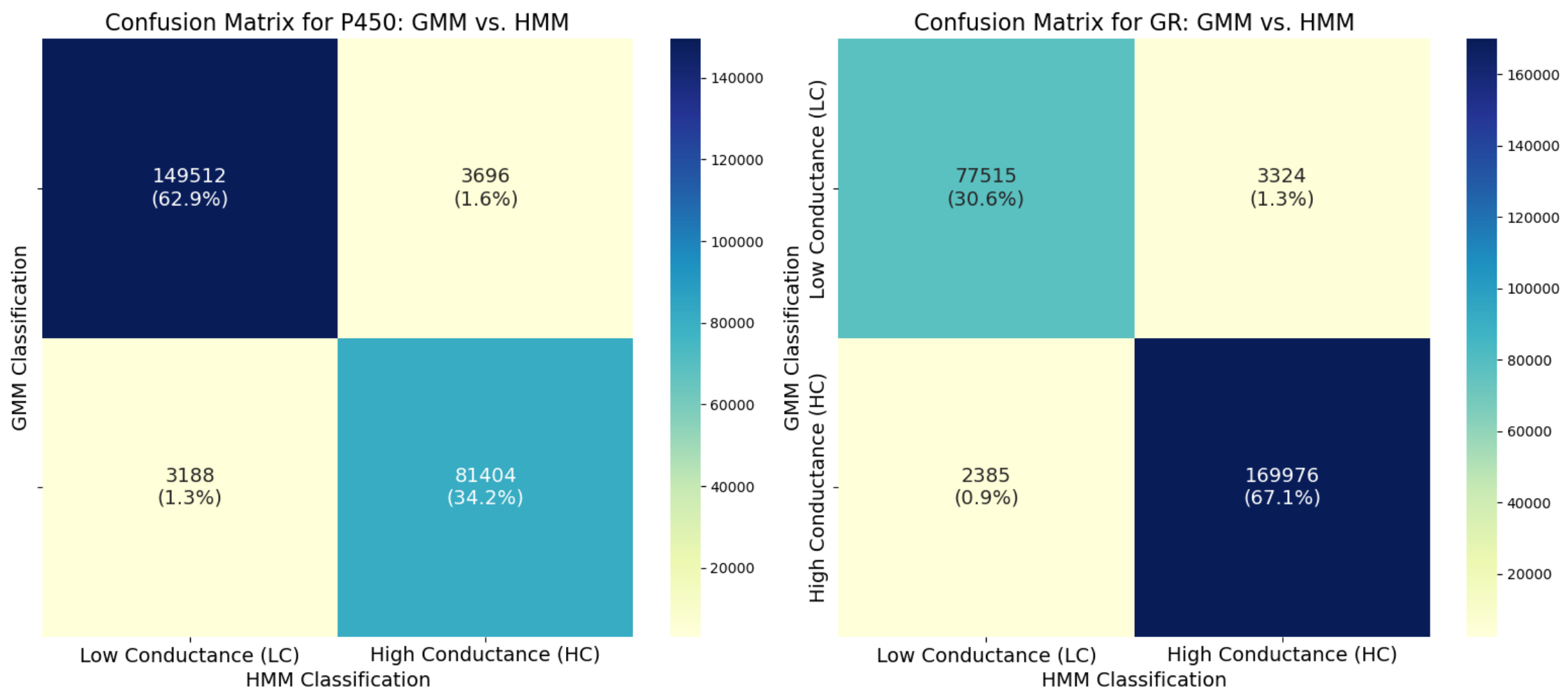

Supplementary Figure S23. Confusion matrices describing the GMM vs. HMM classifications obtained for both P450 (left) and GR (right) proteins. Each cell shows the number of classified data points, indicating the agreement between GMM and HMM classifications, with the corresponding percentage with respect to the total dataset. The diagonal cells represent the cases of agreement between GMM and HMM: the top left diagonal cell shows the number of data points classified as LC by both methods, while the bottom right diagonal cell shows the number of observations classified as HC by both methods. Off-diagonal cells represent discrepancies: the top right cell shows cases where the GMM identified LC while the HMM identified HC, and the bottom left cell shows cases where the GMM identified HC while the HMM identified LC. The percentages refer to the proportion of each classification relative to the total data set.

4. Enzyme catalytic cycles and protein electrostatics.

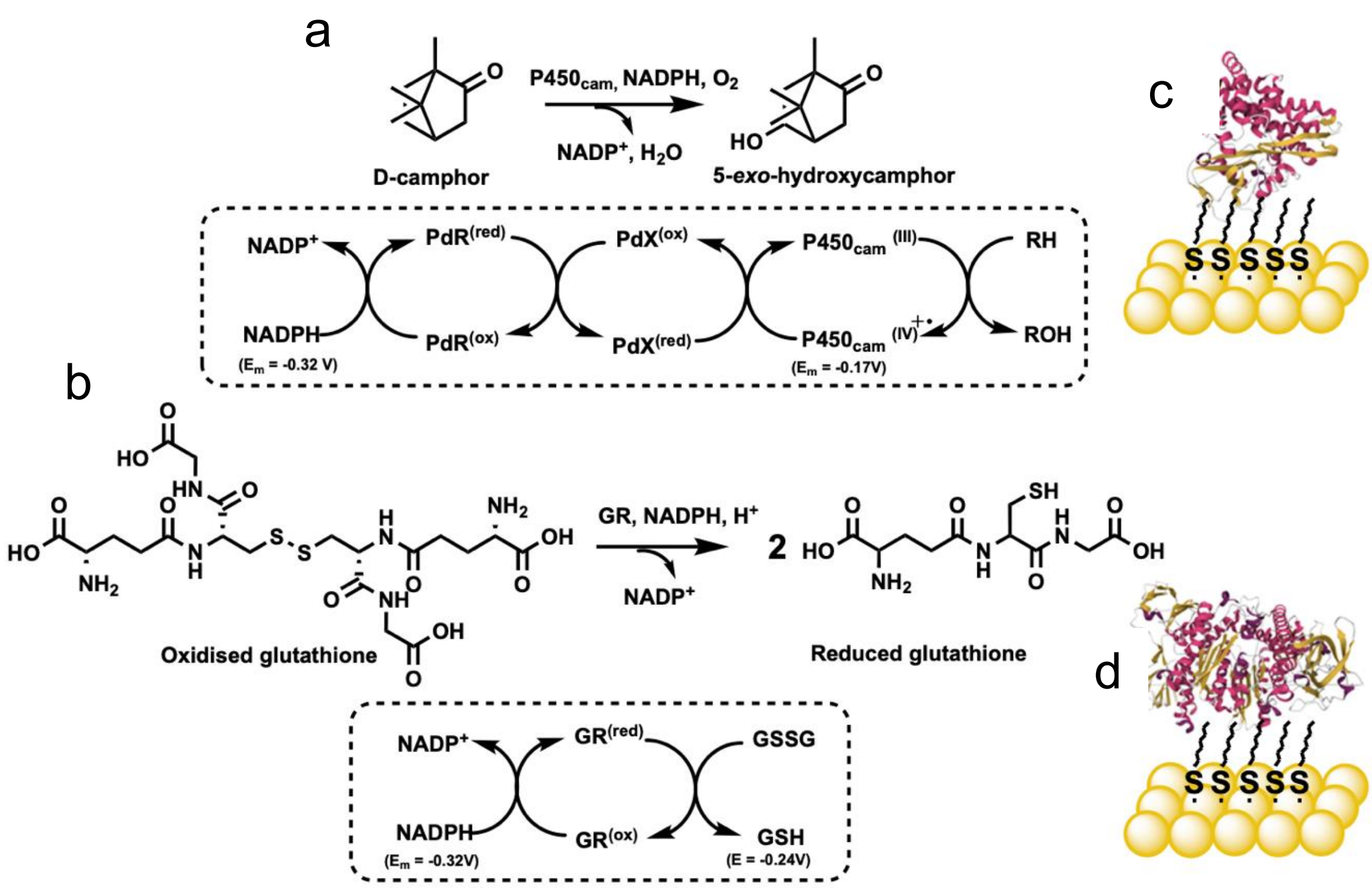

Supplementary Figure S.24. **Natural redox cycle** of (a) $P450_{cam}$ for the hydroxylation of camphor to 5-*exo*-hydroxycamphor begins with the donation of electrons from NADPH to electron transfer partners, putidaredoxin reductase (PdX) and putidaredoxin (PdX), before reducing the *heme* iron centre $Fe(IV)^{\bullet+}$ to Fe(II) and in turn hydroxylating D-camphor to 5-*exo*-hydroxycamphor and (b) glutathione reductase (GR) for the reduction of oxidised glutathione (GSSG) to reduced glutathione (GSH) through the addition of two electrons donated by the cofactor NADPH. The catalytic cycle of glutathione reductase begins with NADPH donating its electrons to the cofactor FAD in oxidised GR, then the transfer of these electrons to GSSG breaking its disulfide bond to produce two molecules of GSH. Schematic diagram illustrating the enzyme-electrode interface where (c) $P450_{cam}$ and (d) GR were adsorbed on 1-octanethiol, a monothiol self-assembled monolayer functionalised on gold electrodes. The reported mid-point potential of NADPH, camphor-bound $P450_{cam}$ and GSSG/GSH couple has been reported to be -0.32 V[18], -0.17 V[19] and -0.24 V[20], respectively, *vs* NHE.

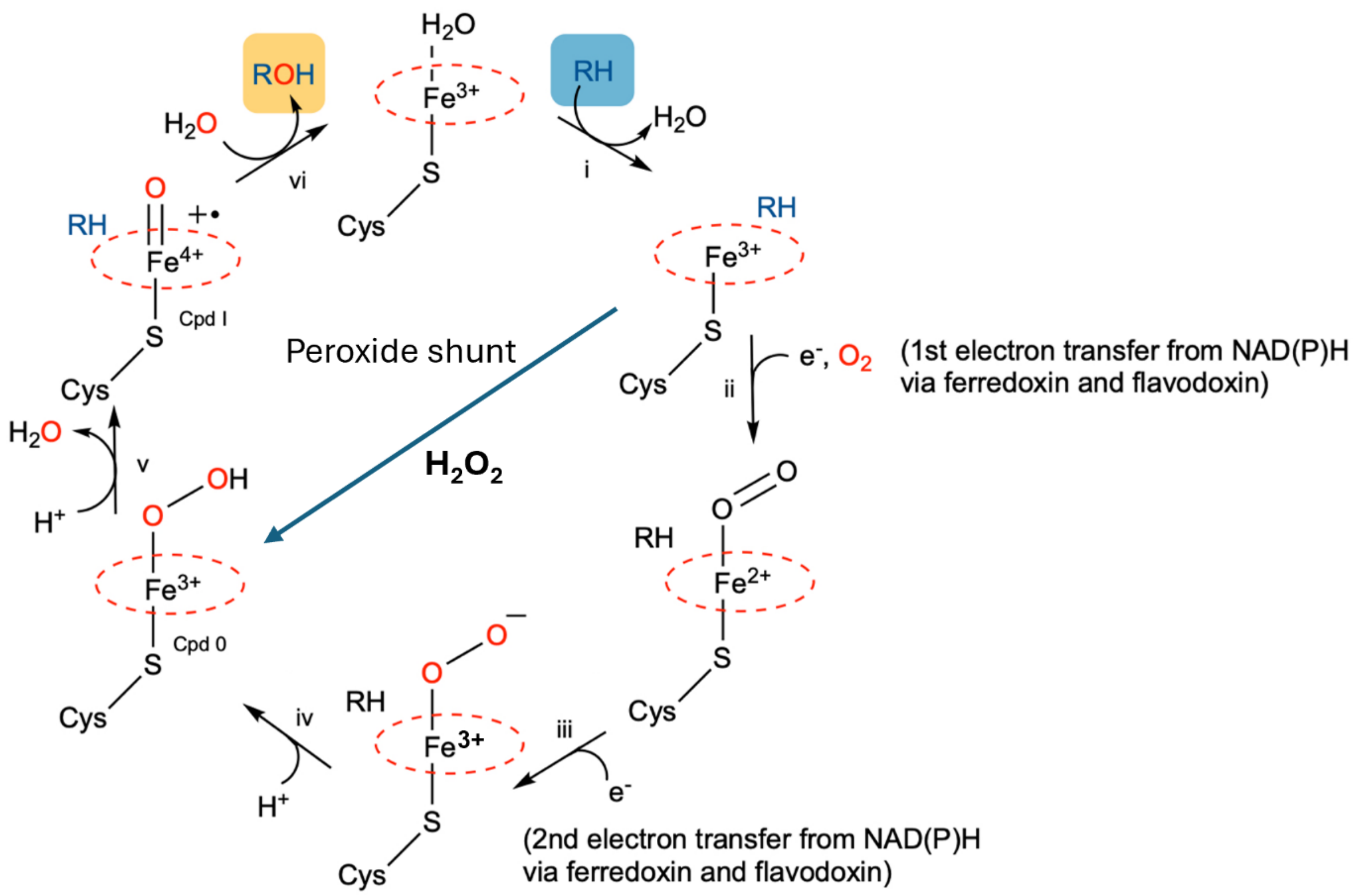

Supplementary Fig. S25. Catalytic cycle of P450$_{cam}$[21]. The catalytic cycle begins with i) substrate (RH) binding: the substrate camphor binds to the iron *heme* centre of the six-coordinate complex, kicking out a water molecule through coordination with the iron atom that induces a change in the Fe^{3+} state from a low spin to high spin, five coordinate complex. ii) reduction: the enzyme is reduced by an electron donor such as NADPH via electron transfer proteins (Fig S24), which generates a high spin Fe^{2+} complex and results in oxygen binding. molecular oxygen bound to ferrous *heme* results in electron transfer from the *heme* iron to oxygen to generate ferric iron and oxygen radical. iii) second reduction: electron transfer from NADPH and generates a peroxy intermediate. iv) protonation results in the hydroperoxo intermediate Compound 0 (Cpd 0). vi) The hydroperoxide intermediate is unstable and quickly undergoes homolytic cleavage, releasing a water molecule and generating an iron-oxygen species $Fe^{(IV/V)}$=O that is stabilised by a porphyrin radical, and known as, Compound I (Cpd I). The active oxidant Cpd I abstracts a hydrogen atom from the C-H bond of the substrate generating a substrate carbon centred radical. v) substrate oxidation: the Fe-ligated O atom combines with the substrate radical to form the hydroxylated product via a so-called oxygen-rebound mechanism. The product (ROH) is then released and the enzyme returns to its low spin state. Note: Cpd 0 can also be formed via a peroxide shunt mechanism whereby the high-spin substrate bound intermediate binds hydrogen peroxide directly with loss of H^+. In biochemical reactions, H_2O_2 can be added to solution directly or formed *in situ* via oxygen reduction.

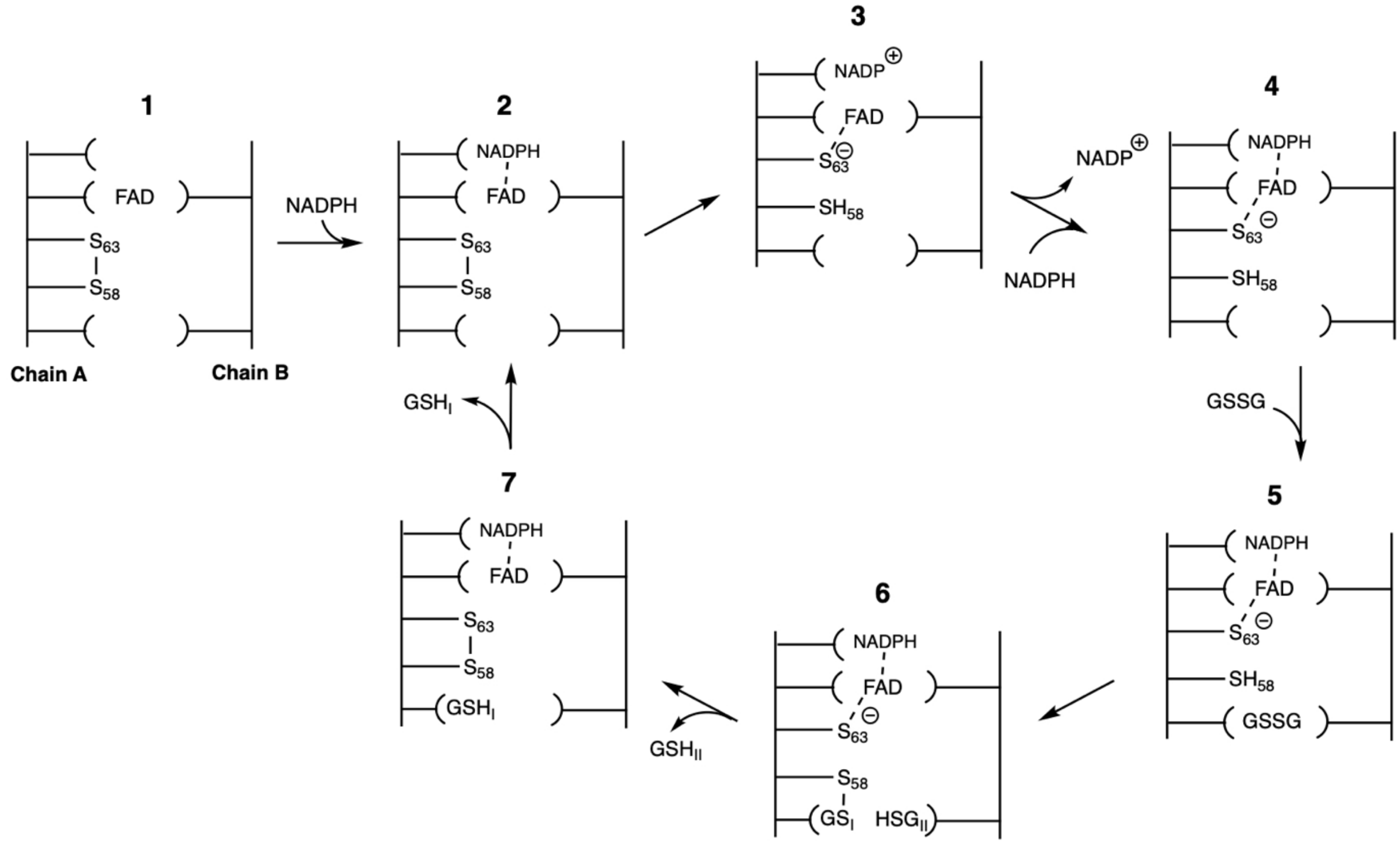

Supplementary Fig. S26. Catalytic cycle of GR[22]. Dotted lines indicate charge-transfer complexes between NADPH, FAD, and the sulfur atom of Cys63. 1. Native state of the enzyme 2. Cycle starts with NADPH binding to oxidised GR. An electron is donated from NADPH to FAD 3. Reduced FAD reduces Cys58-Cys63 disulfide bond to form a covalent intermediate with Cys63 4. Second molecule of NADPH binds to GR 5. GSSG binds to GR 6. Cys58 attacks Glutathione I (GSH_I where the second electron is donated) 7. Glutathione II (GSH_{II} leaves) 8. Cys58 and Cys63 reform the disulfide bond and GSH_I leaves returning to step 2.

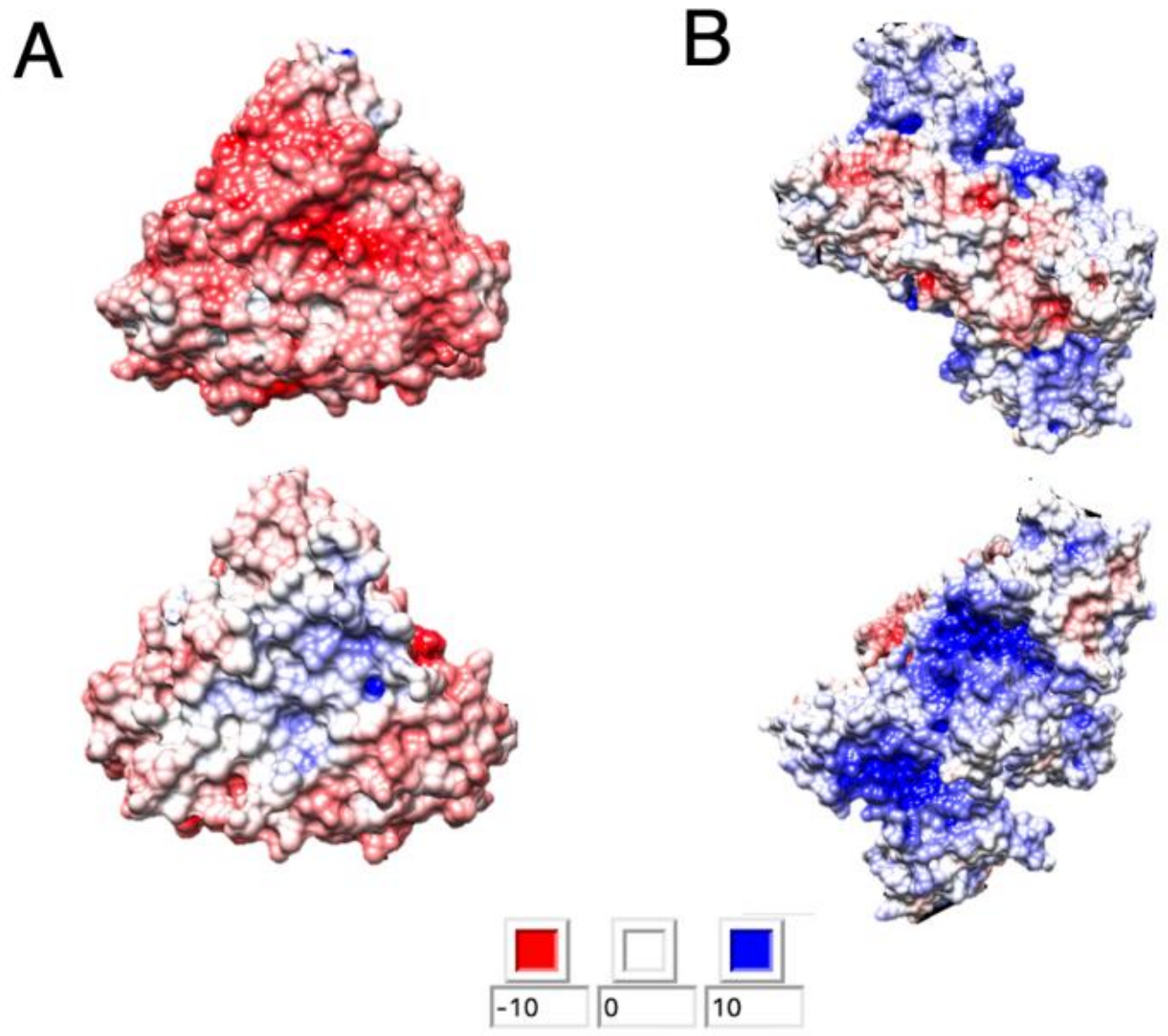

Supplementary Fig. S27. Protein surface electrostatics. Surface electrostatic potentials of (A) $P450_{cam}$ and (B) GR at pH 7.

5. STEADY-STATE KINETICS

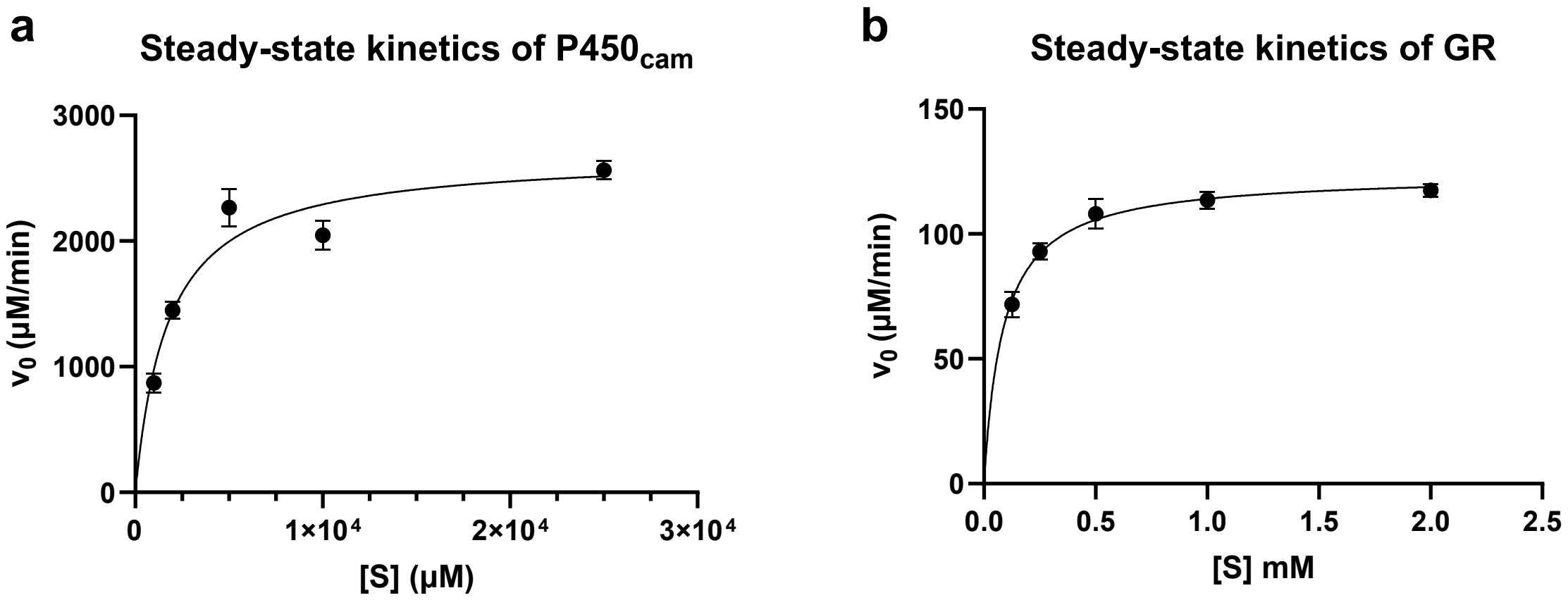

Supplementary Fig. S28. Steady-state kinetics of $p450_{cam}$ and GR. a. For $p450_{cam}$: Initial velocities (v_0) were measured at 25 ˚C in 100mM phosphate buffer pH 7.0 with shaking at 800 rpm for 1 minute. Assays contained 1 µM $P450_{cam}$, *Spinacia oleracea* ferredoxin (Fd) and ferredoxin–$NADP^+$ reductase (FNR) at a molar ratio of P450:Fd:FNR = 1:1.2:0.02. Camphor was supplied at 1–25 mM (1,000–25,000 µM). Concentrations below 1 mM were excluded because camphor was fully consumed during the initial-rate window and did not yield reliable v_0 measurements. Buffer, enzyme and camphor were pre-incubated for 10 min, followed by Fd and FNR and further incubation for 5 min before initialising turnover by the addition of 200 µM NADPH. Substrate consumption was quantified by HPLC, and peak areas were converted to concentrations using an external calibration curve of camphor. Each point represents the mean ± SD of three replicate measurements at each substrate concentration (n = 3). The solid line corresponds to a nonlinear least-squares fit to the Michaelis–Menten equation $\upsilon = \frac{V_{max}[S]}{K_M+[S]}$, performed in GraphPad Prism 10.5.0, yielding V_{max} = 2689 ± 130 µM · min^{-1} (95% CI 2447-2957 µM · min^{-1} and K_M = 1765 ± 325 µM (95% CI 1195-2468 µM); with $[E]_0$= 1 µM, this corresponds to k_{cat} ≈ 2689 min^{-1}. **b.** For GR: Initial velocities were measured at 25 °C in 100 mM Tris–HCl buffer pH 8.0, containing 0.2 mM NADPH. Glutathione reductase (yeast GR; 118 kDa homodimer) was used at a final concentration of 0.001 mg mL^{-1} (corresponding to 0.0085 µM dimer). Reactions were initiated by the addition of GSSG at the indicated concentrations (0.125–2 mM). NADPH consumption was monitored at 340 nm, and rates were obtained from the linear region of the absorbance traces using ε_{340} = 6.22 mM^{-1} cm^{-1}. Each point represents the mean ± SD of three replicate reaction traces (n = 3). The solid line corresponds to a nonlinear least-squares fit to the Michaelis–Menten equation $\upsilon = \frac{V_{max}[S]}{K_M+[S]}$, yielding V_{max} = 123.9 ± 4.3 µM min^{-1} (95% CI 119.6–128.4 µM min^{-1}) and K_M = 86 ± 16 µM (95% CI 71–103 µM). Using $[E]_0$= 0.0085 µM GR dimer, the corresponding catalytic turnover number is k_{cat} ≈ 1.46 × 10^4 min^{-1} per dimer (≈7.3 × 10^3 min^{-1} per monomer). These values are fully consistent with previously reported steady-state parameters for yeast GR under similar assay conditions.

References:

1. Tan, C. Y. *et al.* Immobilization of a Bacterial Cytochrome P450 Monooxygenase System on a Solid Support. *Angewandte Chemie* **128**, 15226–15230 (2016).

2. Schenkman, B. J. & Ingela, J. *Spectral Analyses of Cytochromes P450.Pdf*. *Paper Knowledge . Toward a Media History of Documents* (1998).

3. Chapman, S. K., Reid, G. A., Aliverti, A., Curti, B. & Vanoni, M. A. *Identifying and Quantitating FAD and FMN Identifying and Quantitating FAD and FMN in Simple and in Iron-Sulfur-Containing Flavoproteins*. (1999).

4. Johnson, E. O. D. & Wong, L. L. Partial fusion of a cytochrome P450 system by carboxy-terminal attachment of putidaredoxin reductase to P450cam (CYP101A1). *Catal Sci Technol* **6**, 7549–7560 (2016).

5. White, R. E., McCarthy, M.-B., Egeberg, K. D. & Sligar, S. G. Regioselectivity in the cytochromes P-450: Control by protein constraints and by chemical reactivities. *Arch Biochem Biophys* **228**, 493–502 (1984).

6. Veskoukis, A. S., Margaritelis, N. V., Kyparos, A., Paschalis, V. & Nikolaidis, M. G. Spectrophotometric assays for measuring redox biomarkers in blood and tissues: the NADPH network. *Redox Report* vol. 23 47–56 Preprint at https://doi.org/10.1080/13510002.2017.1392695 (2018).

7. Stauffer, C. & Grimson, W. E. L. Adaptive background mixture models for real-time tracking. *Proceedings. 1999 IEEE Computer Society Conference on Computer Vision and Pattern Recognition (Cat. No PR00149)* 246–252 (1999) doi:10.1109/CVPR.1999.784637.

8. Plataniotis, K. N. & Hatzinakos, D. Gaussian Mixtures and Their Applications to Signal Processing. in *Advanced Signal Processing Handbook* (CRC Press, 2001).

9. Ju, Z. & Liu, H. Fuzzy Gaussian Mixture Models. *Pattern Recognit* **45**, 1146–1158 (2012).

10. Rabiner, L. R. A tutorial on hidden Markov models and selected applications in speech recognition. *Proceedings of the IEEE* **77**, 257–286 (1989).

11. Domke, K. F. & Aragonès, A. C. Playing catch and release with single molecules: mechanistic insights into plasmon-controlled nanogaps. *Nanoscale* **15**, 497–506 (2022).

12. Singh, D., Punia, B. & Chaudhury, S. Theoretical Tools to Quantify Stochastic Fluctuations in Single-Molecule Catalysis by Enzymes and Nanoparticles. *ACS Omega* **7**, 47587–47600 (2022).

13. Su, D. *et al.* Stochastic Binding Dynamics of a Photoswitchable Single Supramolecular Complex. *Advanced Science* **9**, (2022).

14. Markov. A. A. The theory of algorithms (in Russian). *The Academy of Sciences of the U.S.S.R* **42**, 3–375 (1975).

15. Rocha, M. & Ferreira, P. G. Hidden Markov Models. in *Bioinformatics Algorithms* 255–273 (Elsevier, 2018). doi:10.1016/B978-0-12-812520-5.00012-2.

16. Koomson, D. A., Nicholson, J. H., Brogan, A. P. S. & Aldous, L. Re-assessing viologens for modern bio-electrocatalysis. *Chem Sci* **15**, 9325–9332 (2024).

17. Kazlauskaite, J., Westlake, Andrew. C. G., Wong, L.-L. & Hill, H. A. O. Direct electrochemistry of cytochrome P450cam. *Chemical Communications* 2189–2190 (1996).

18. Roome, P. W. & Peterson, J. A. The reduction of putidaredoxin reductase by reduced pyridine nucleotides. *Arch Biochem Biophys* **266**, 32–40 (1988).

19. Gunsalus, I. C., Lipscomb, J. D. & Meeks, J. R. Cytochrome P450cam Substrate and effector interactions. *Ann N Y Acad Sci* **212**, 107–121 (1973).

20. Schafer, F. Q. & Buettner, G. R. Redox environment of the cell as viewed through the redox state of the glutathione disulfide/glutathione couple. *Free Radic Biol Med* **30**, 1191–1212 (2001).

21. Belcher, J. *et al.* Structure and Biochemical Properties of the Alkene Producing Cytochrome P450 OleTJE (CYP152L1) from the Jeotgalicoccus sp. 8456 Bacterium. *Journal of Biological Chemistry* **289**, 6535–6550 (2014).

22. Berkholz, D. S., Faber, H. R., Savvides, S. N. & Karplus, P. A. Catalytic Cycle of Human Glutathione Reductase Near 1 Å Resolution. *J Mol Biol* **382**, 371–384 (2008).
